# Supplementary figures and images for: Emergence of Dip2-mediated specific DAG-based PKC signalling axis in eukaryotes
Source: eLife. 2025 May 6;14:RP104011. doi: 10.7554/eLife.104011 (PMC12055004; doi:10.7554/eLife.104011)

Figure 1-source data 1

## Control

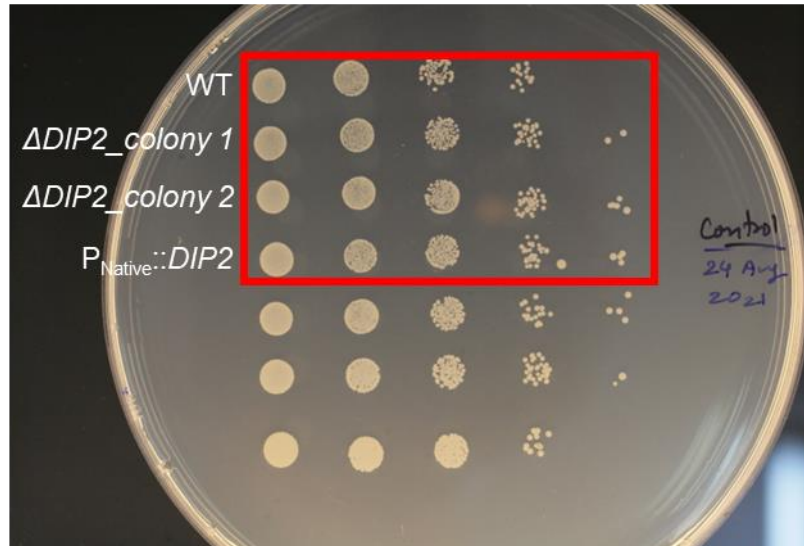

## CW stress (CR)

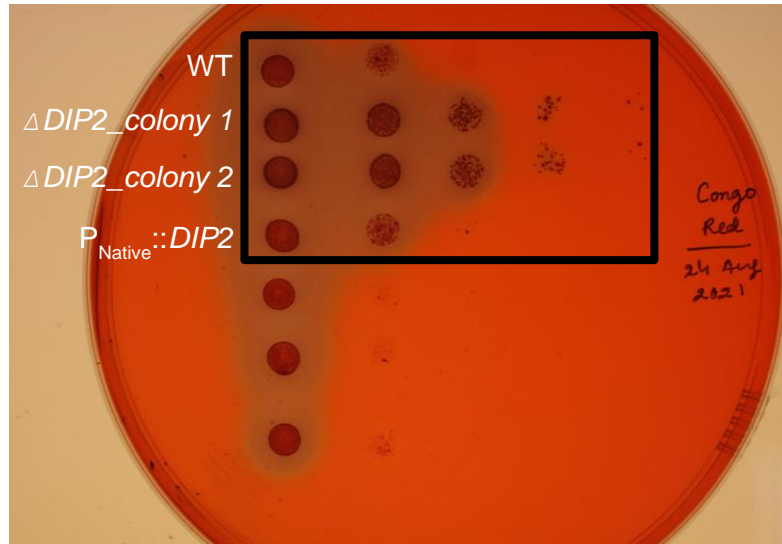

## CW stress (CR)

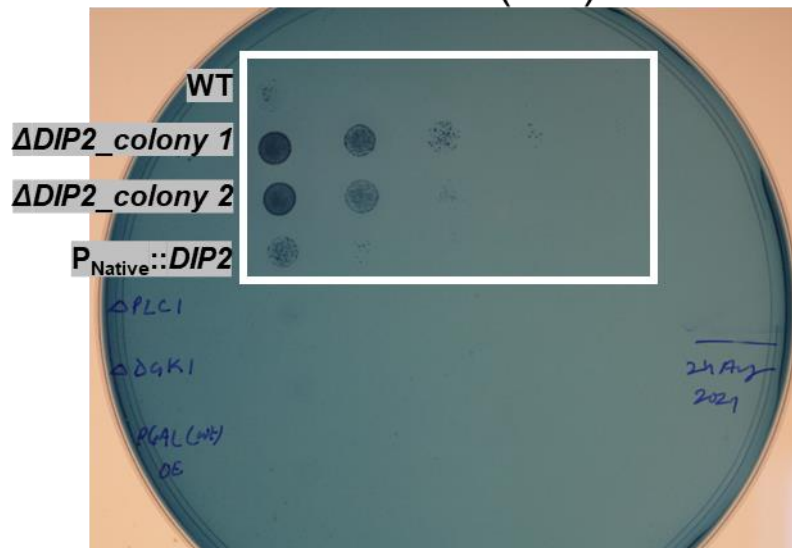

Supplement: Figure 1—source data 1. — PDF file containing original spot assay plate images for Figure 1B, indicating the relevant spots and treatments. [file elife-104011-fig1-data1.zip › Figure 1-source data 1/Figure 1-source data 1.pdf]

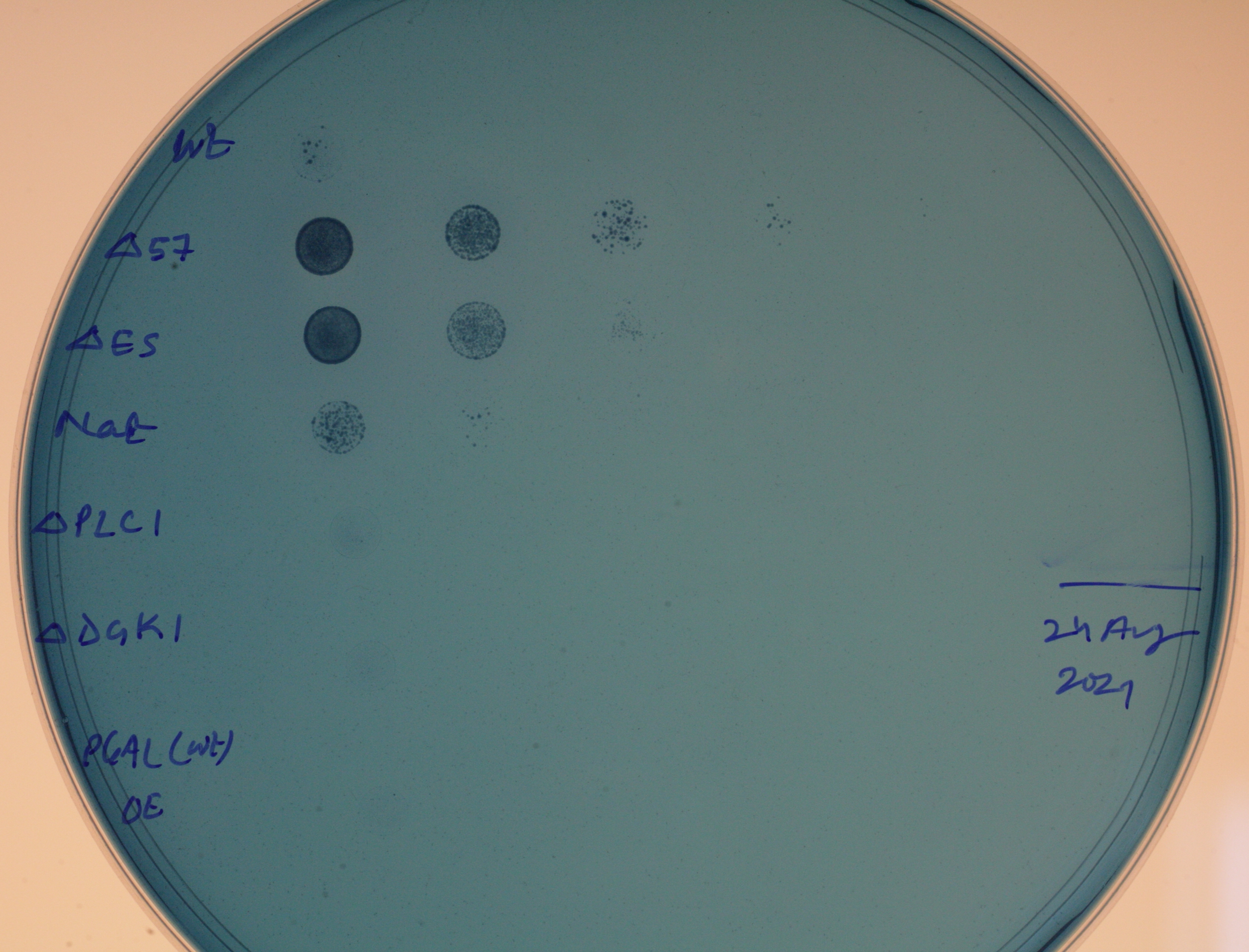

Supplement: Figure 1—source data 2. — Original files for spot assay plate images displayed in Figure 1B. [file elife-104011-fig1-data2.zip › Figure 1-source data 2/Related to Fig 1B CFW.tif]

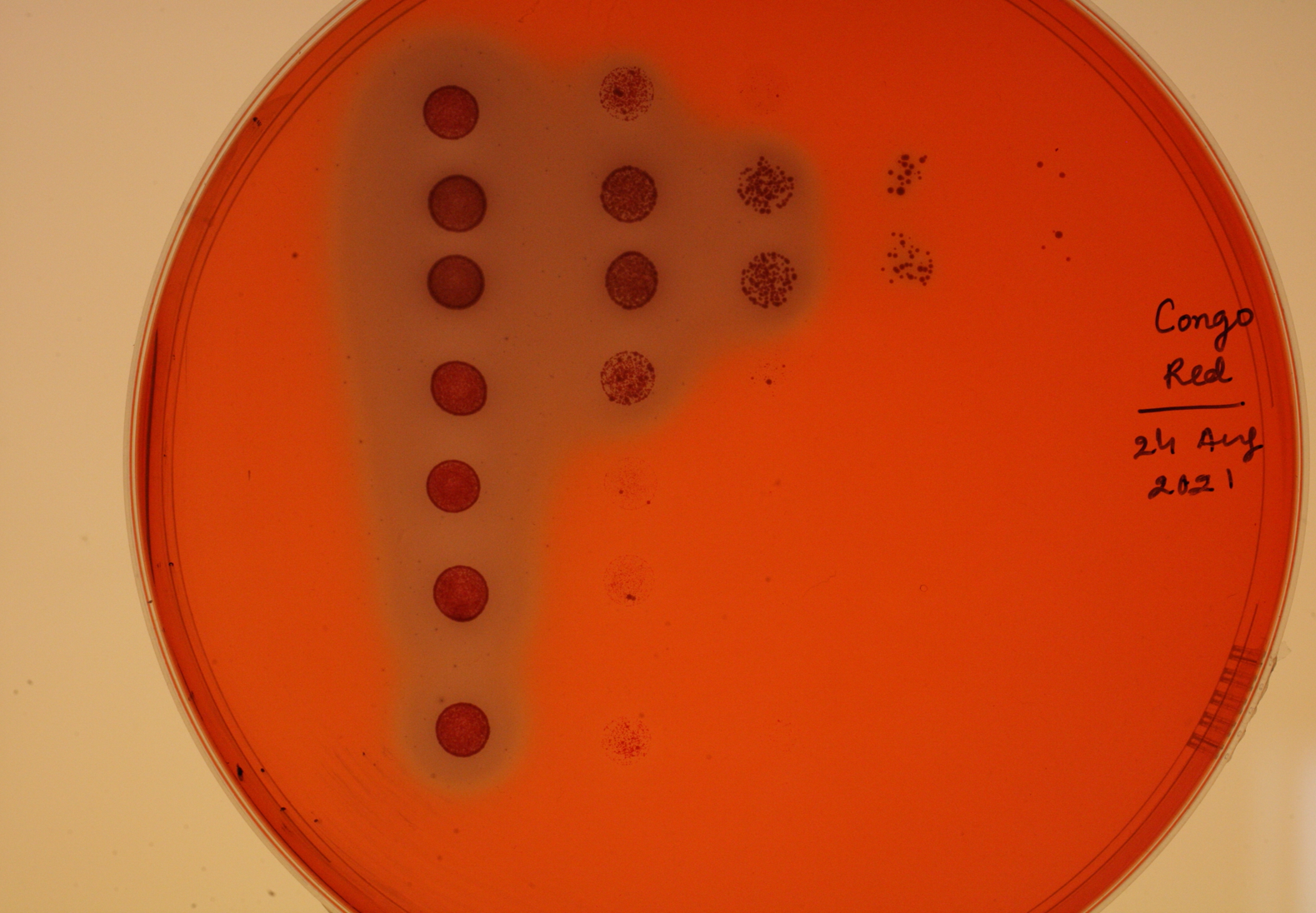

Supplement: Figure 1—source data 2. — Original files for spot assay plate images displayed in Figure 1B. [file elife-104011-fig1-data2.zip › Figure 1-source data 2/Related to Fig 1B Congo red.tif]

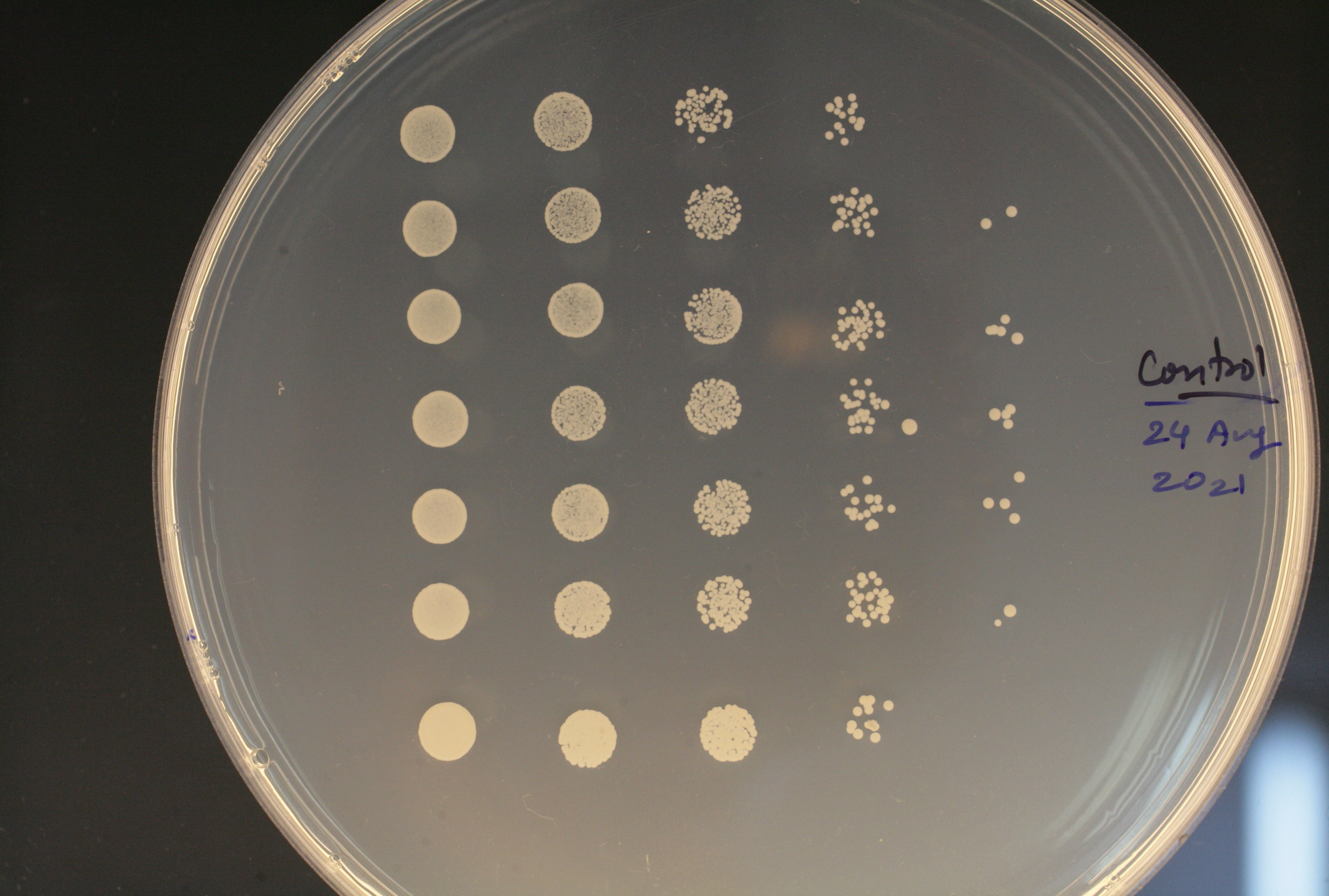

Supplement: Figure 1—source data 2. — Original files for spot assay plate images displayed in Figure 1B. [file elife-104011-fig1-data2.zip › Figure 1-source data 2/Related to Fig 1B control.tif]

Figure 1-source data 4

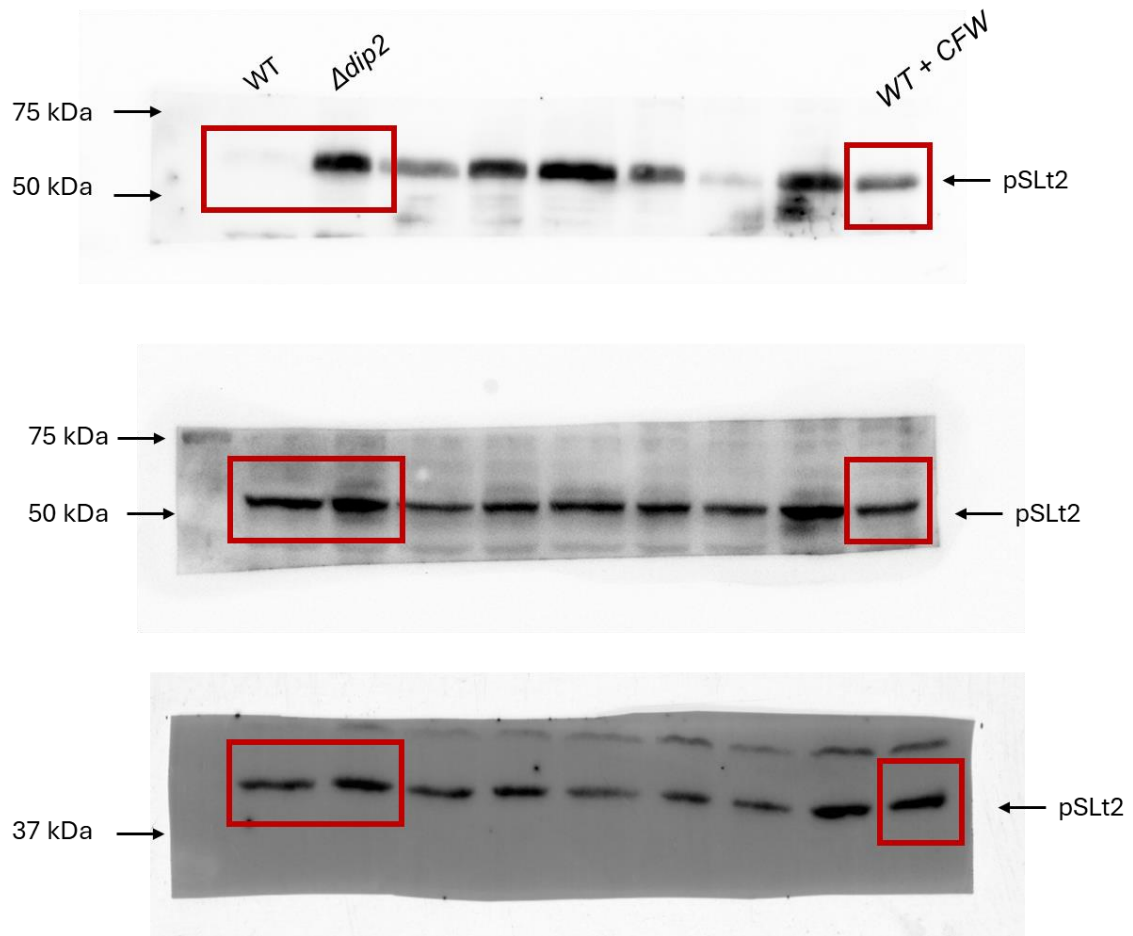

Supplement: Figure 1—source data 4. — PDF file containing original western blots for Figure 1D, indicating the relevant bands and treatments. [file elife-104011-fig1-data4.zip › Figure 1-source data 4/Figure 1-source data 4.pdf]

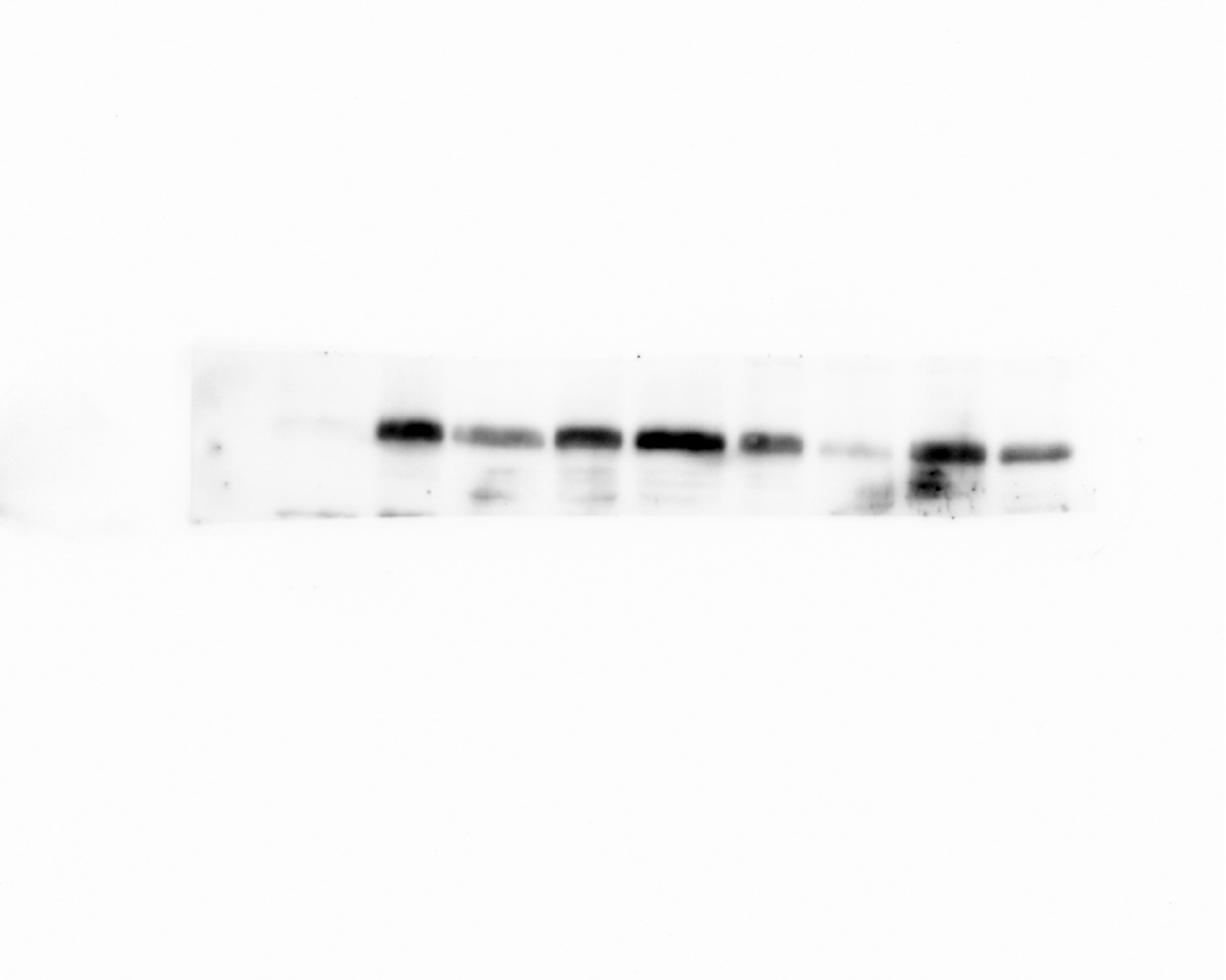

Supplement: Figure 1—source data 5. — Original files for western blot analysis displayed in Figure 1D. [file elife-104011-fig1-data5.zip › Figure 1-source data 5/Related to Fig 1D pSlt2.tif]

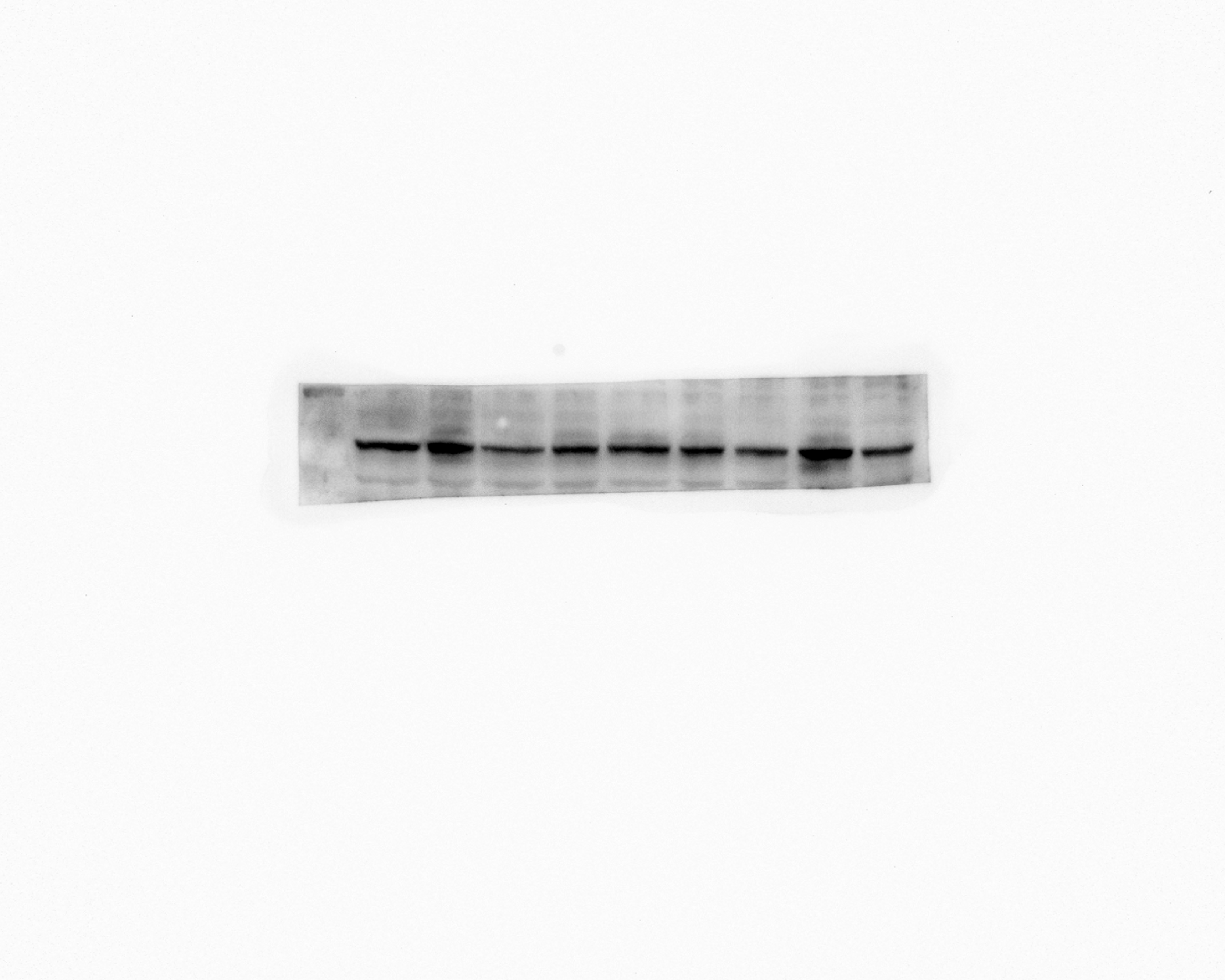

Supplement: Figure 1—source data 5. — Original files for western blot analysis displayed in Figure 1D. [file elife-104011-fig1-data5.zip › Figure 1-source data 5/Related to Fig 1D Slt2.tif]

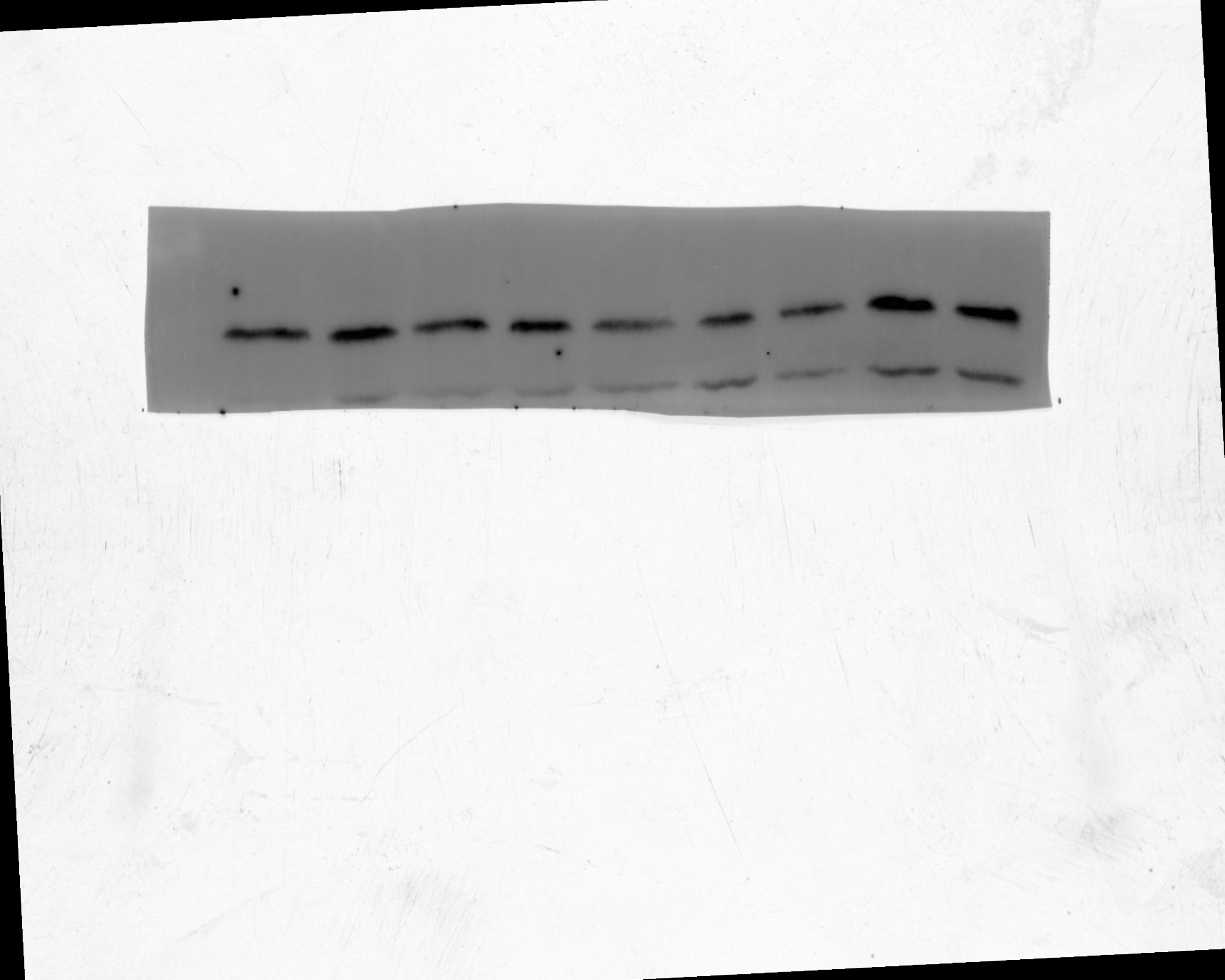

Supplement: Figure 1—source data 5. — Original files for western blot analysis displayed in Figure 1D. [file elife-104011-fig1-data5.zip › Figure 1-source data 5/Related to Fig 1D Tpi1.tif]

Figure 1-source data 6

Control

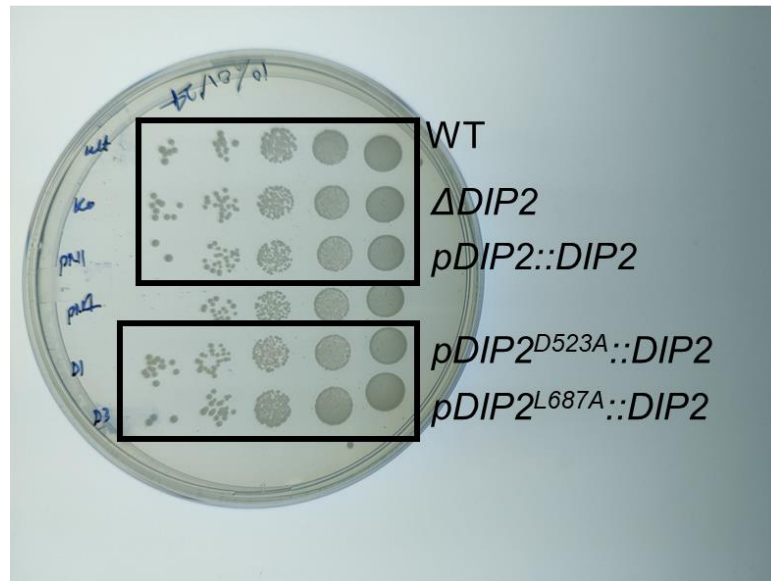

CW stress (CR)

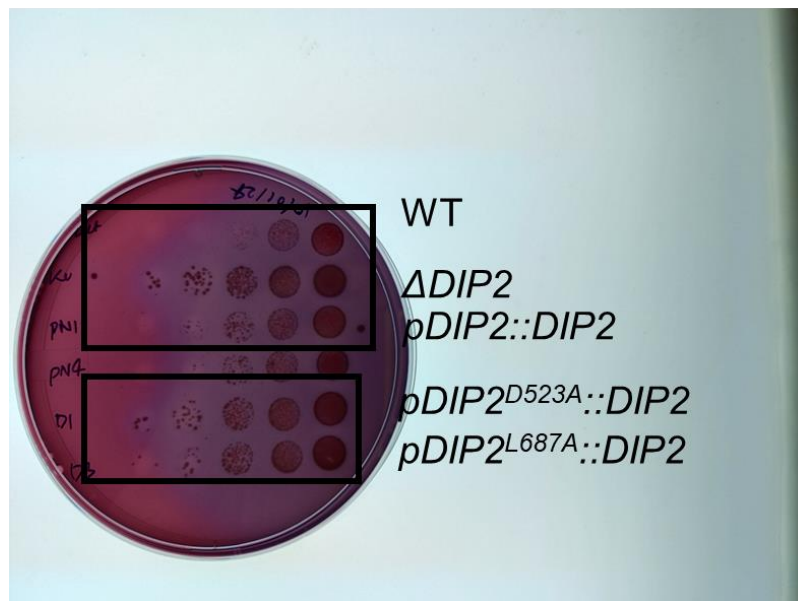

Supplement: Figure 1—source data 6. — PDF file containing original spot assay plate images for Figure 1E, indicating the relevant spots and treatments. [file elife-104011-fig1-data6.zip › Figure 1-source data 6/Figure 1-source data 6.pdf]

Figure 1-source data 8

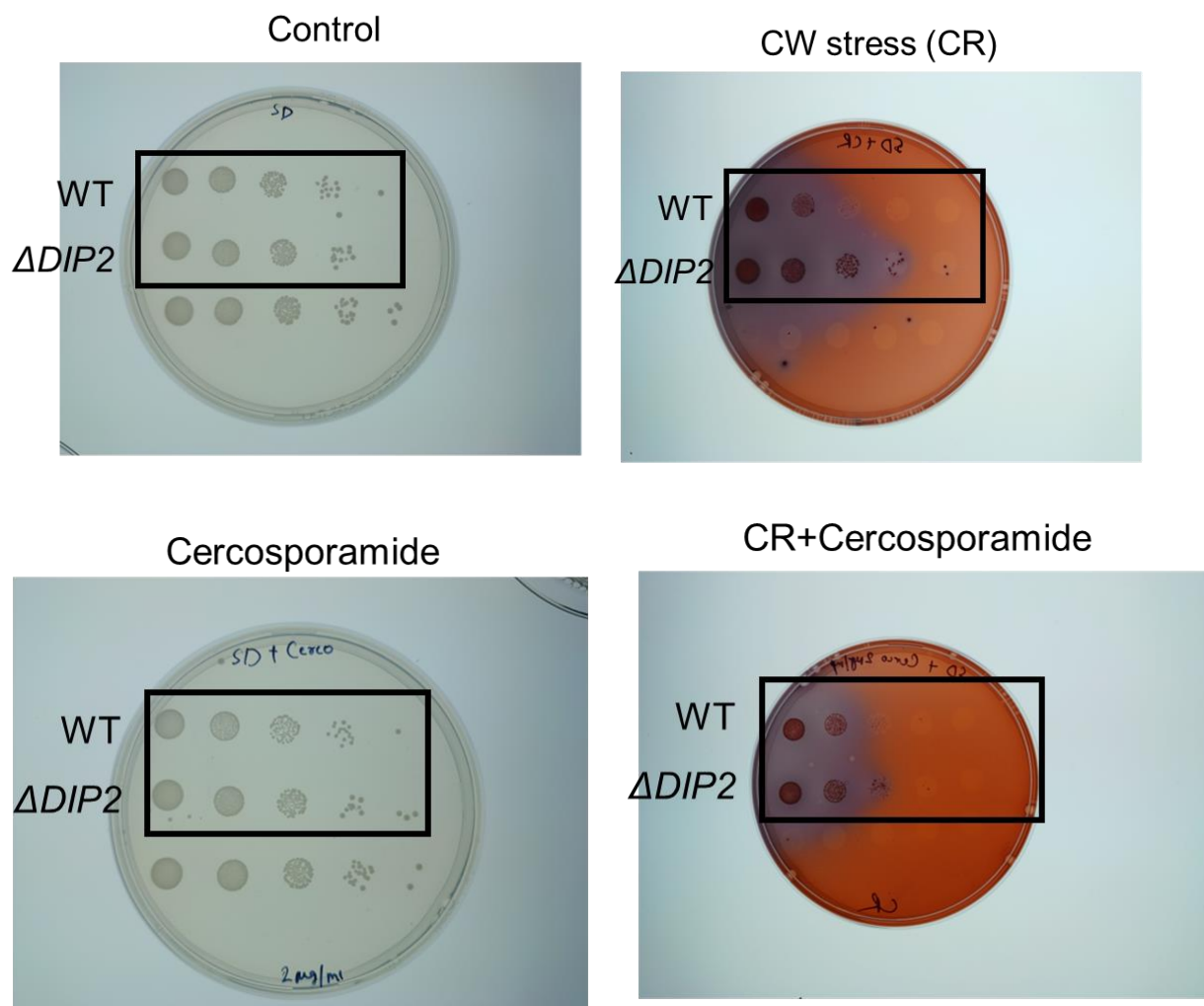

Supplement: Figure 1—source data 8. — PDF file containing original spot assay plate images for Figure 1F, indicating the relevant spots and treatments. [file elife-104011-fig1-data8.zip › Figure 1-source data 8/Related to Fig 1F.pdf]

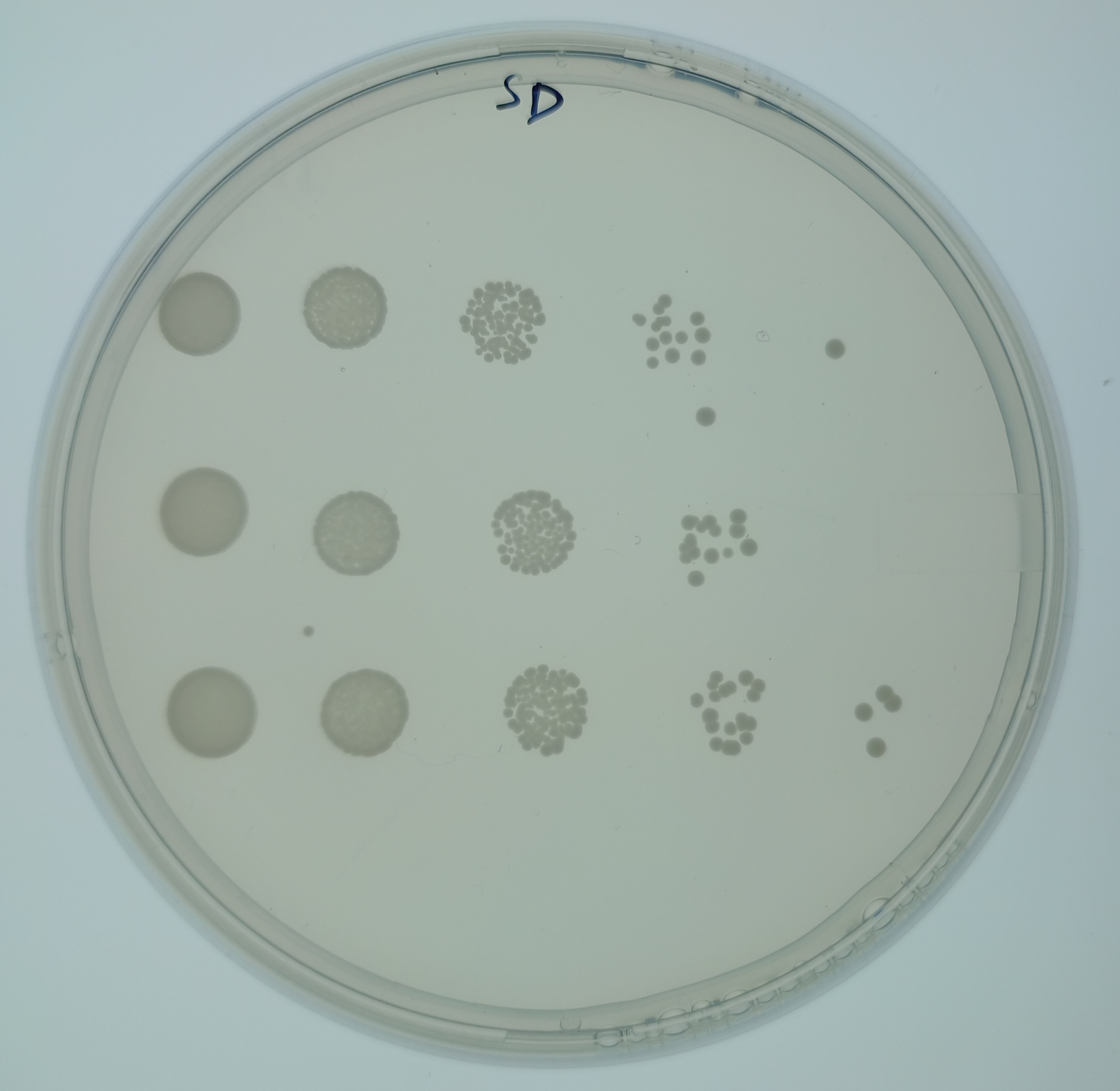

Supplement: Figure 1—source data 9. — Original files for spot assay plate images displayed in Figure 1F. [file elife-104011-fig1-data9.zip › Figure 1-source data 9/Related_to_Fig_1F_Control.jpg]

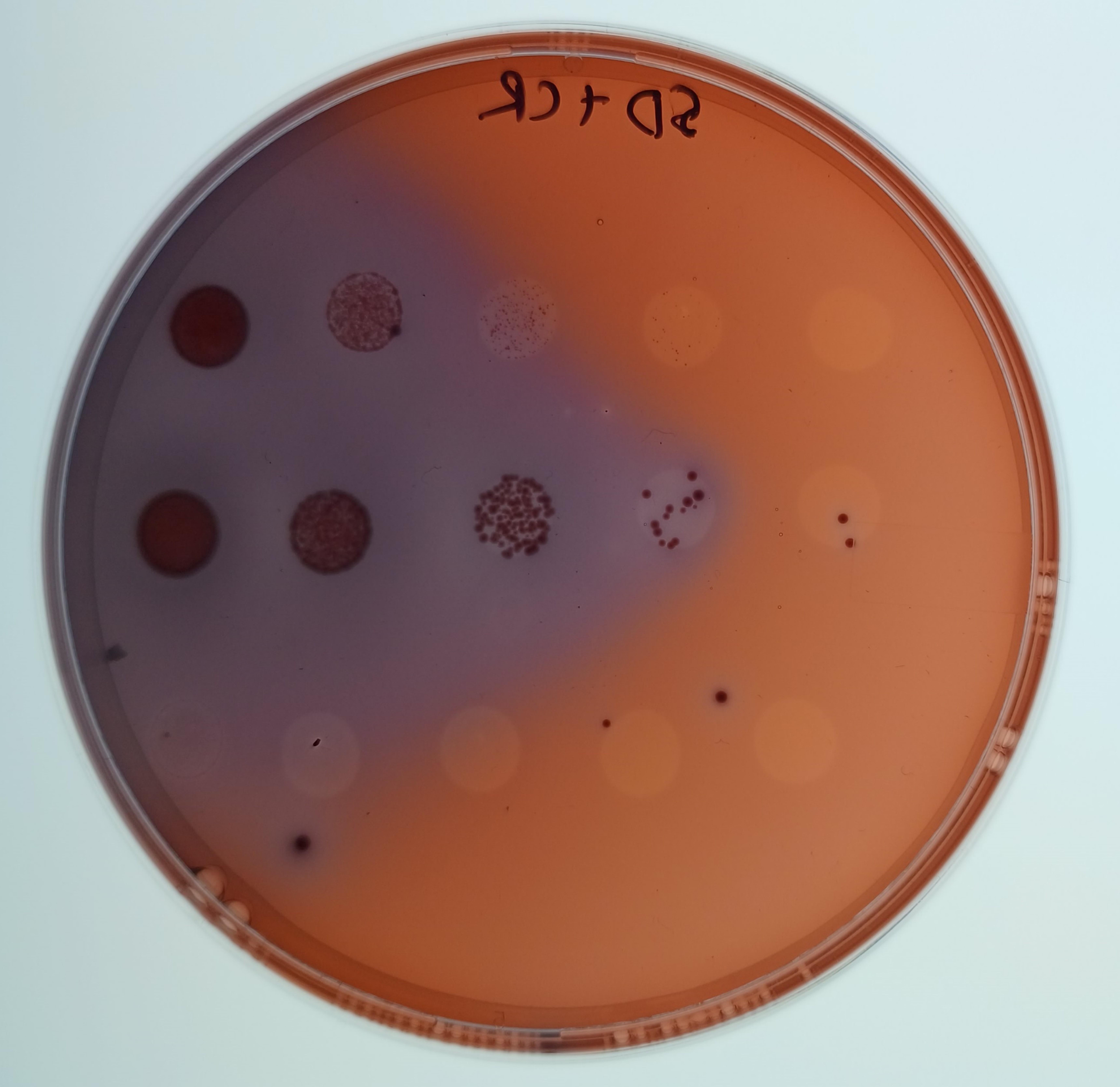

Supplement: Figure 1—source data 9. — Original files for spot assay plate images displayed in Figure 1F. [file elife-104011-fig1-data9.zip › Figure 1-source data 9/Related_to_Fig_1F_CR.jpg]

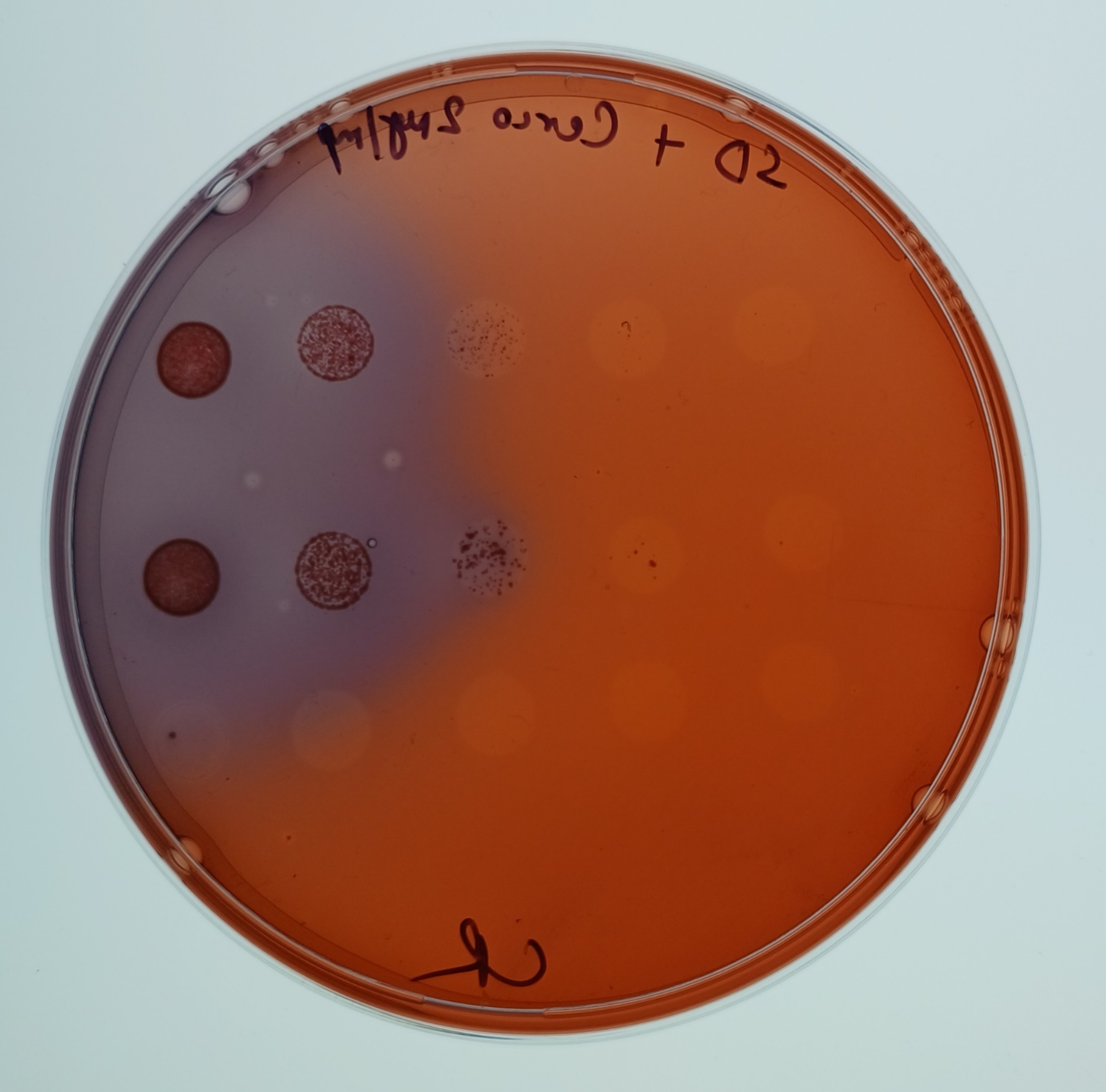

Supplement: Figure 1—source data 9. — Original files for spot assay plate images displayed in Figure 1F. [file elife-104011-fig1-data9.zip › Figure 1-source data 9/Related_to_Fig_1F_CR_cerco.jpg]

Figure 1-source data 10

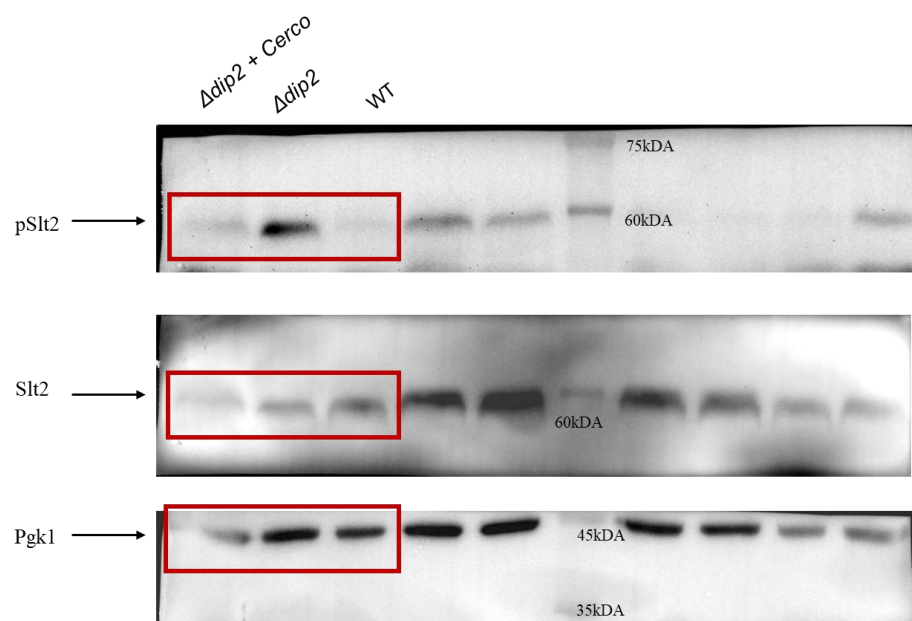

Supplement: Figure 1—source data 10. — PDF file containing original western blots for Figure 1G, indicating the relevant bands and treatments. [file elife-104011-fig1-data10.zip › Figure 1-source data 10/Figure 1-source data 10.pdf]

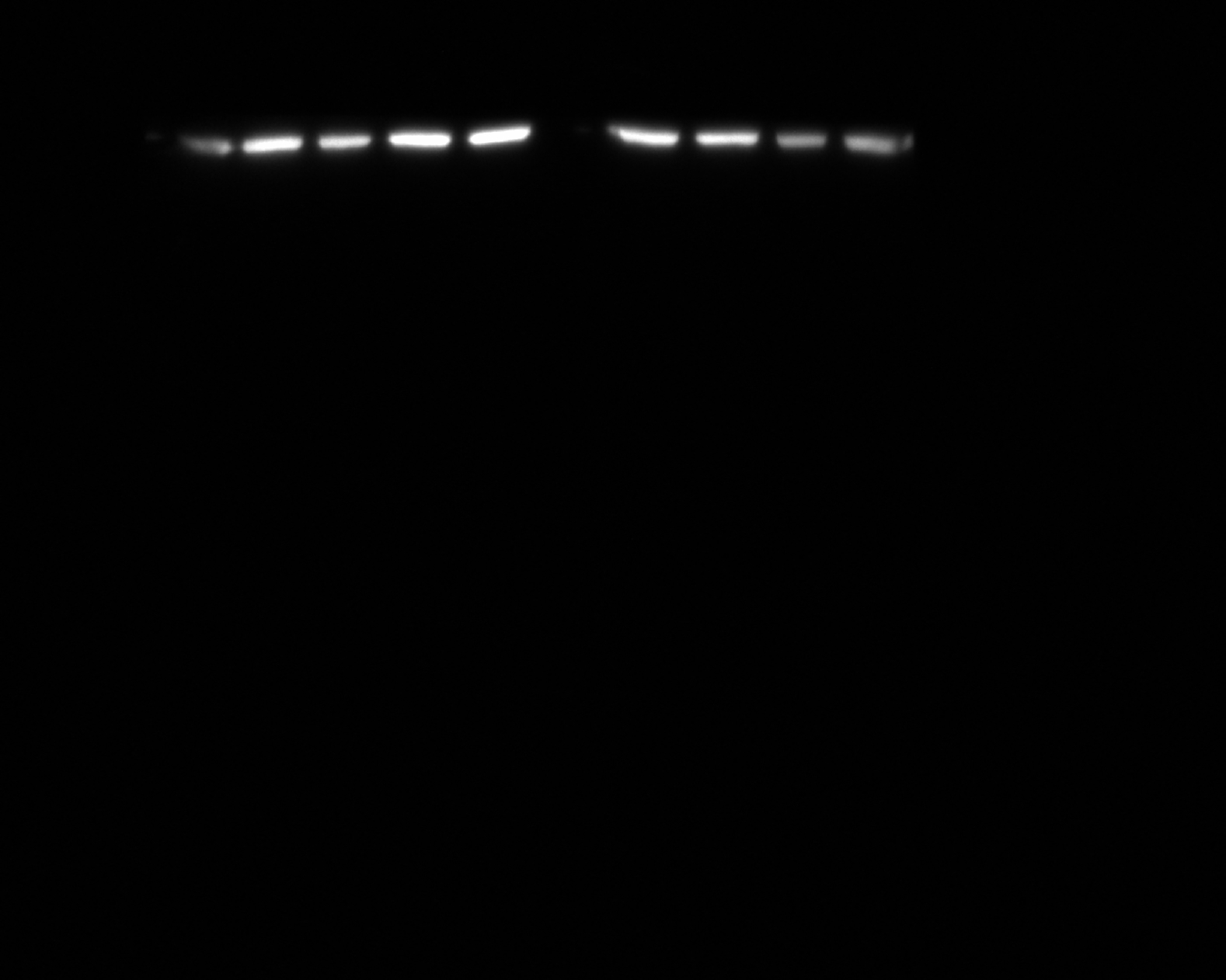

Supplement: Figure 1—source data 11. — Original files for western blot analysis displayed in Figure 1G. [file elife-104011-fig1-data11.zip › Figure 1-source data 11/Related to Fig 1G Pgk1.xlsx.jpg]

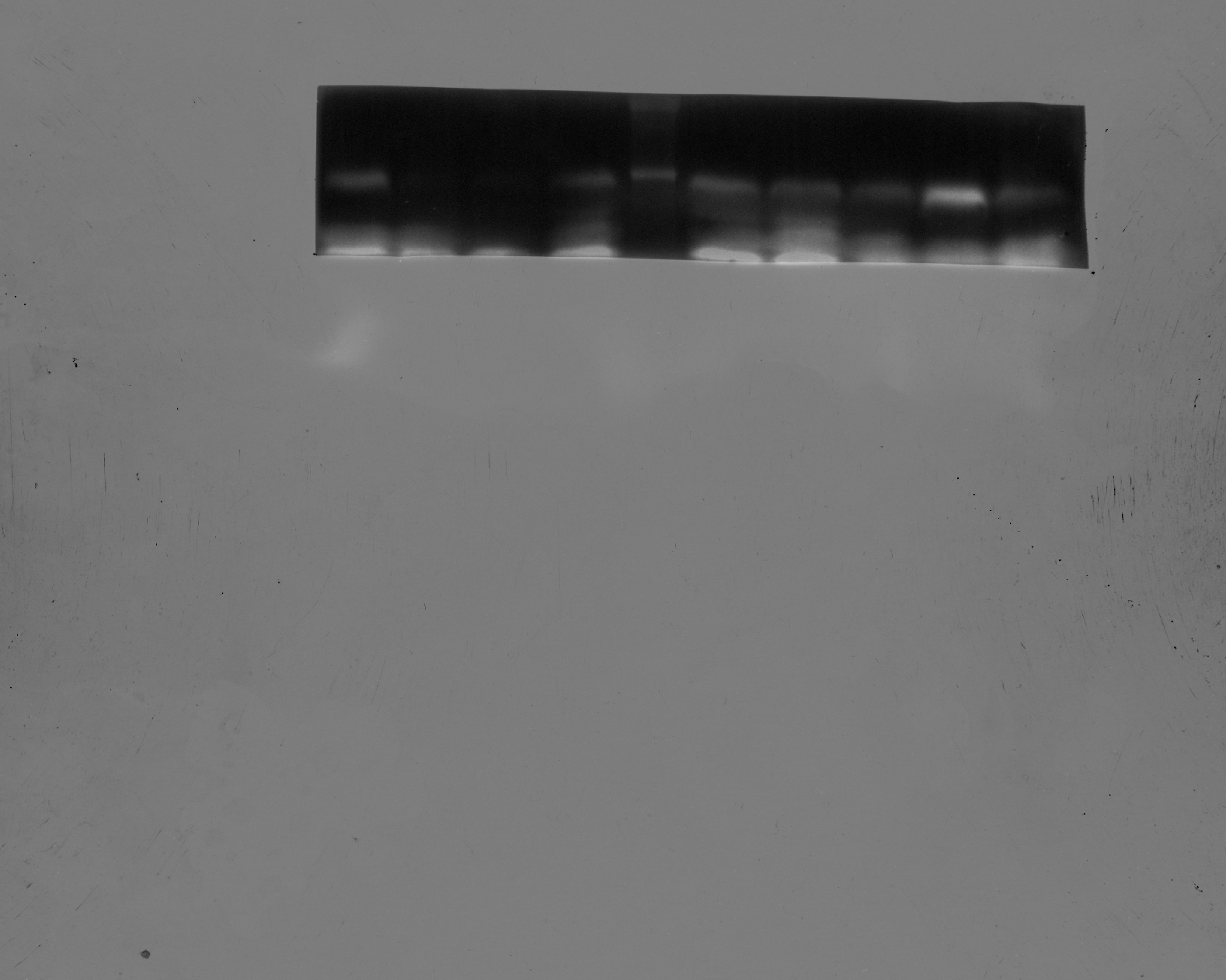

Supplement: Figure 1—source data 11. — Original files for western blot analysis displayed in Figure 1G. [file elife-104011-fig1-data11.zip › Figure 1-source data 11/Related to Fig 1G pSlt2.xlsx.jpg]

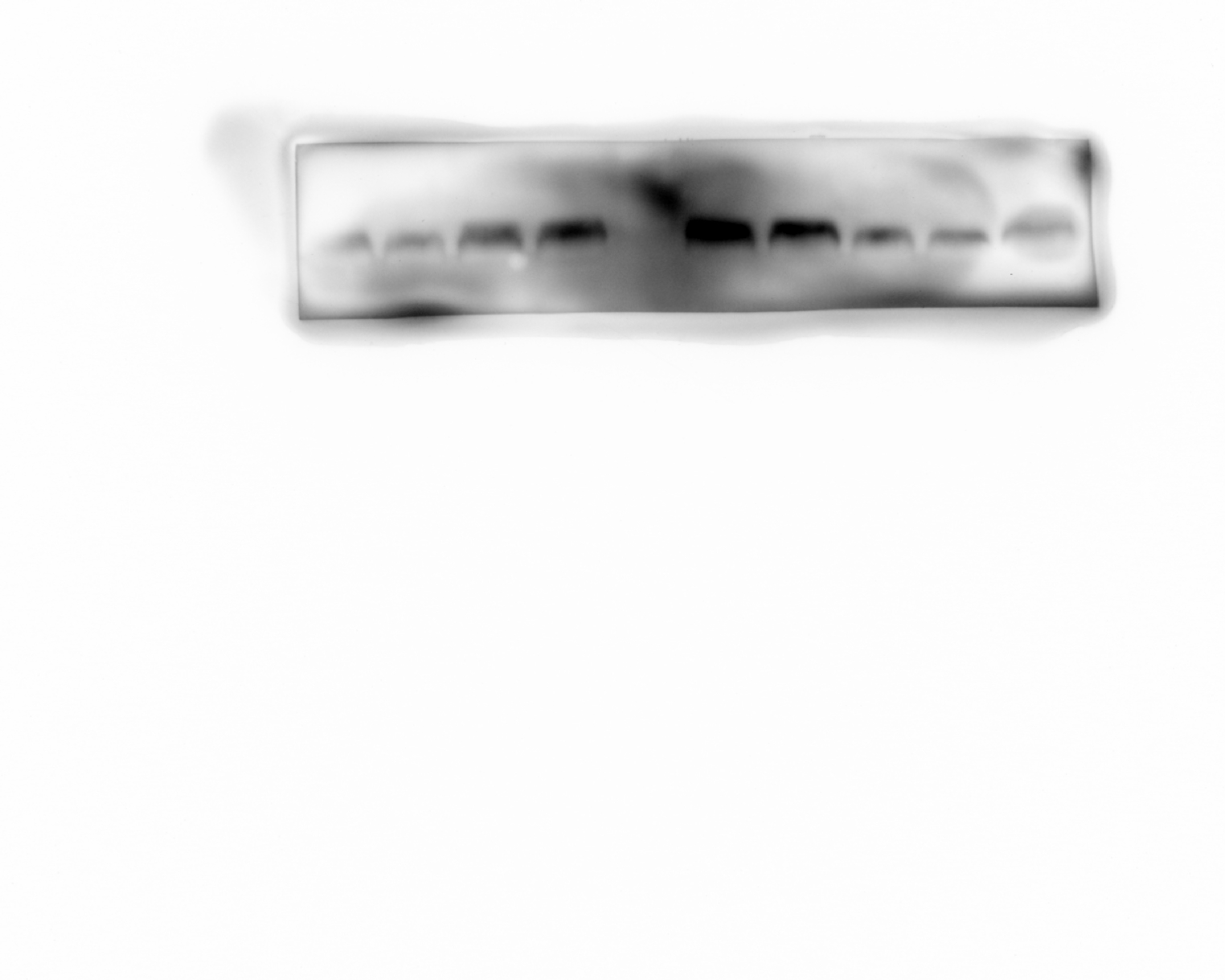

Supplement: Figure 1—source data 11. — Original files for western blot analysis displayed in Figure 1G. [file elife-104011-fig1-data11.zip › Figure 1-source data 11/Related to Fig 1G Slt2.xlsx.jpg]

Figure 1. figure supplement 1

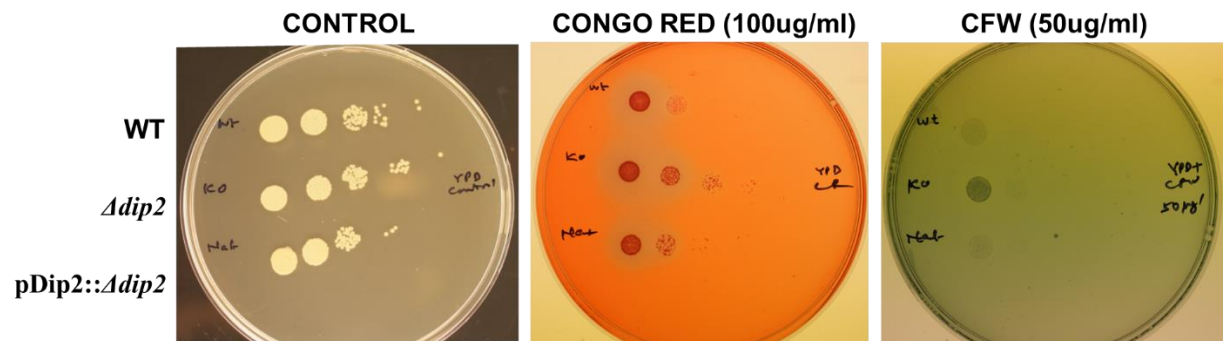

Supplement: Figure 1—figure supplement 1—source data 1. [file elife-104011-fig1-figsupp1-data1.zip › Figure 1. figure supplement 1-Source data 1/Figure 1. figure supplement 1.pdf]

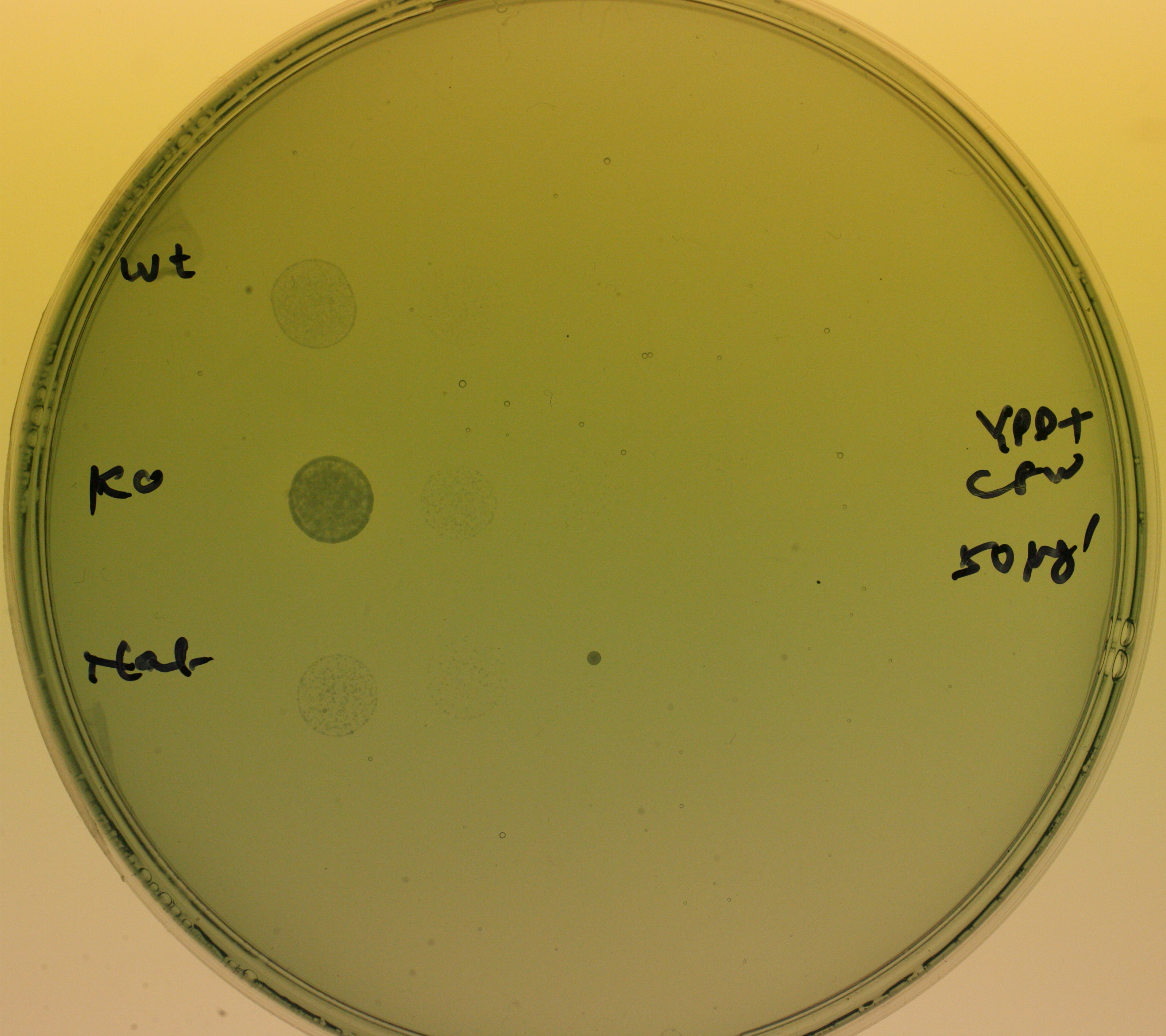

Supplement: Figure 1—figure supplement 1—source data 2. [file elife-104011-fig1-figsupp1-data2.zip › Figure 1. figure supplement 1-Source data 2/Related to Figure 1. figure supplement 1 CFW.tif]

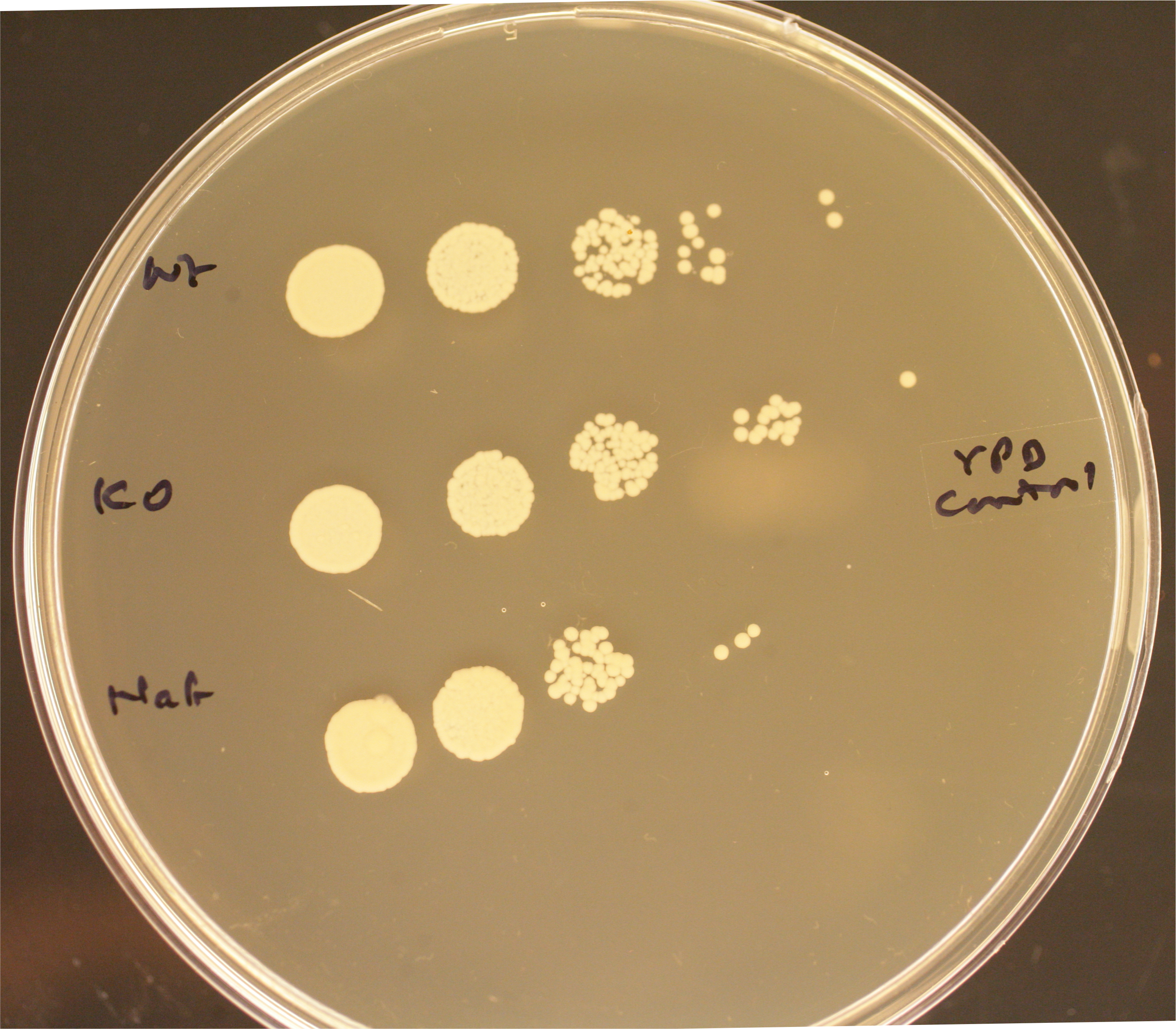

Supplement: Figure 1—figure supplement 1—source data 2. [file elife-104011-fig1-figsupp1-data2.zip › Figure 1. figure supplement 1-Source data 2/Related to Figure 1. figure supplement 1 Control.tif]

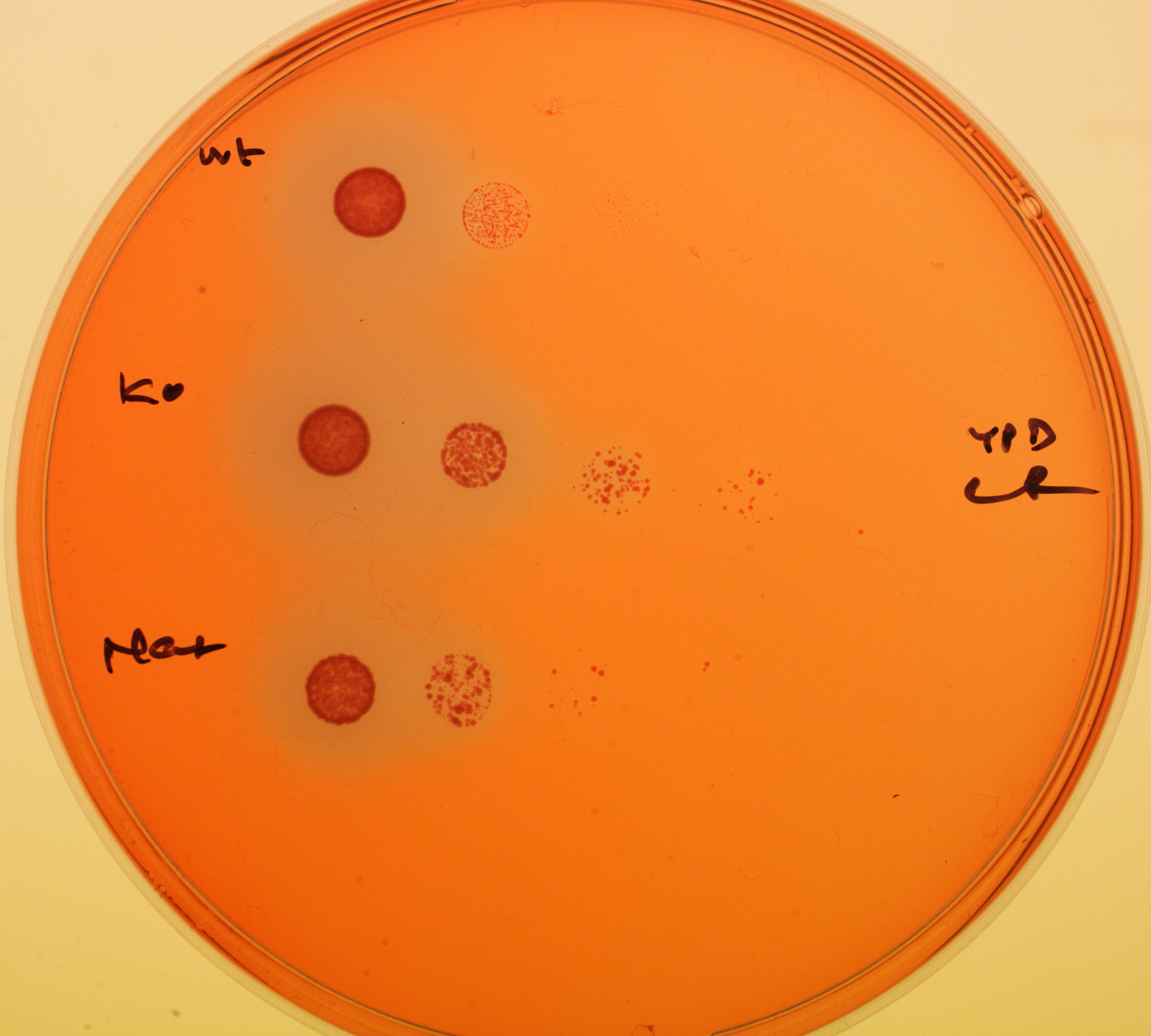

Supplement: Figure 1—figure supplement 1—source data 2. [file elife-104011-fig1-figsupp1-data2.zip › Figure 1. figure supplement 1-Source data 2/Related to Figure 1. figure supplement 1 CR.tif]

**Figure 1. figure supplement 2**

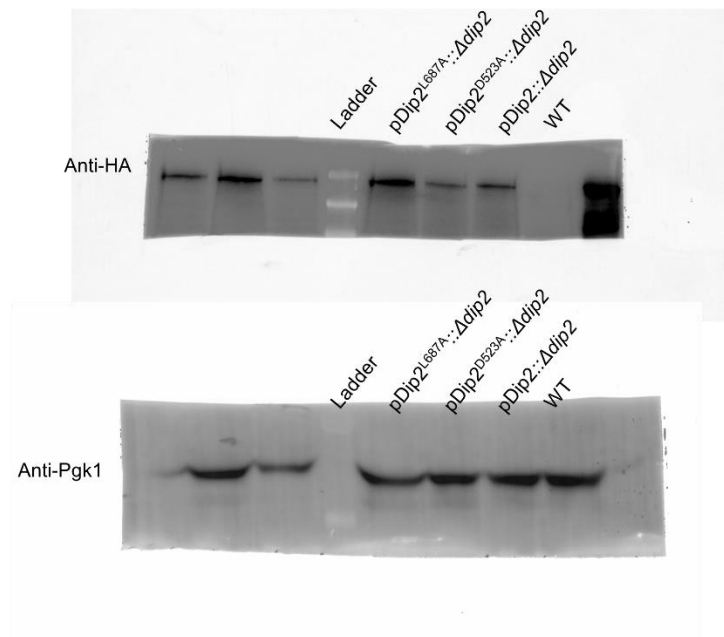

Supplement: Figure 1—figure supplement 2—source data 1. [file elife-104011-fig1-figsupp2-data1.zip › Figure 1. figure supplement 2-Source data 1/Source data Figure 1. figure supplement 2A.pdf]

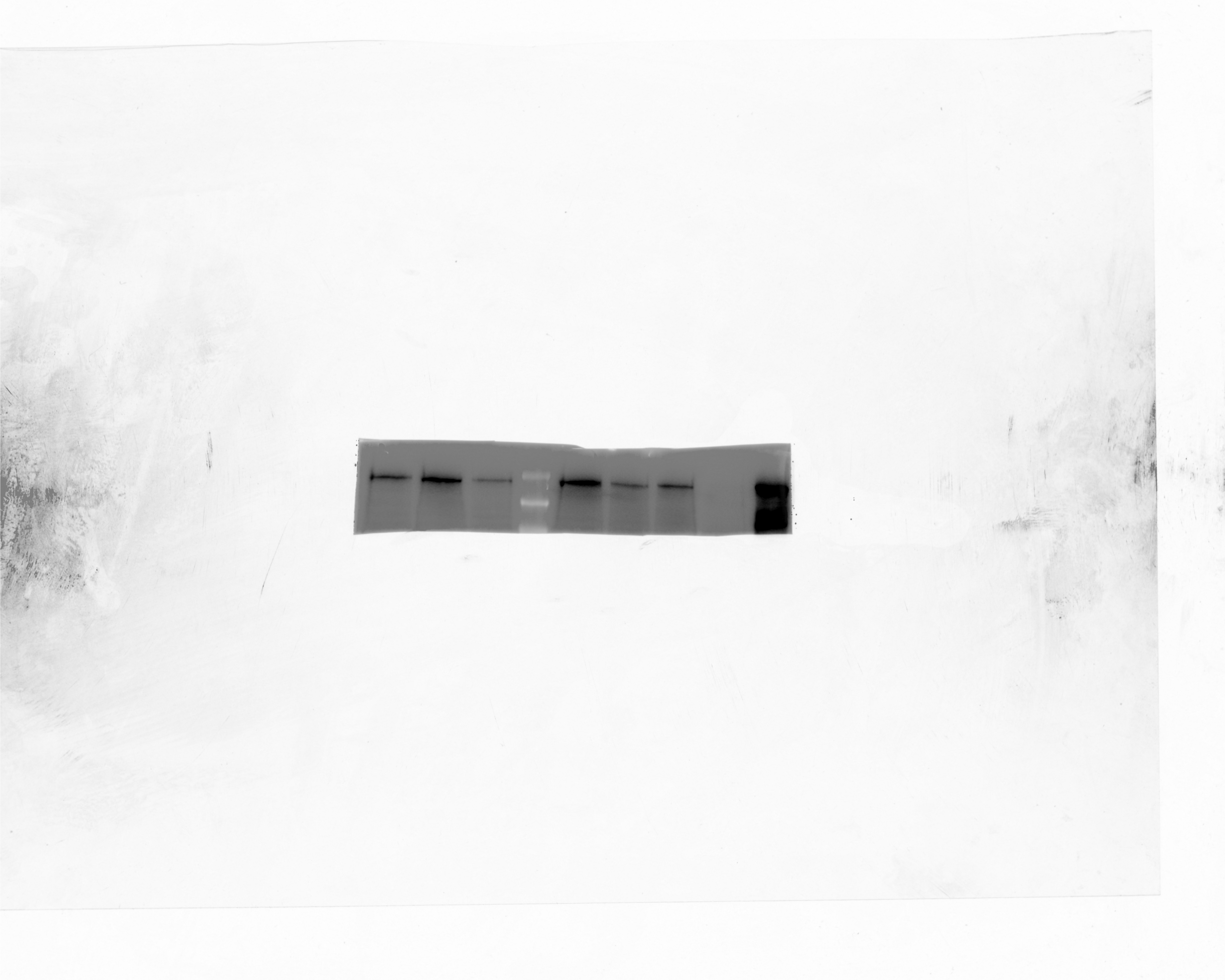

Supplement: Figure 1—figure supplement 2—source data 2. [file elife-104011-fig1-figsupp2-data2.zip › Figure 1. figure supplement 2-Source data 2/Source data Figure 1. figure supplement 2A HA.tif]

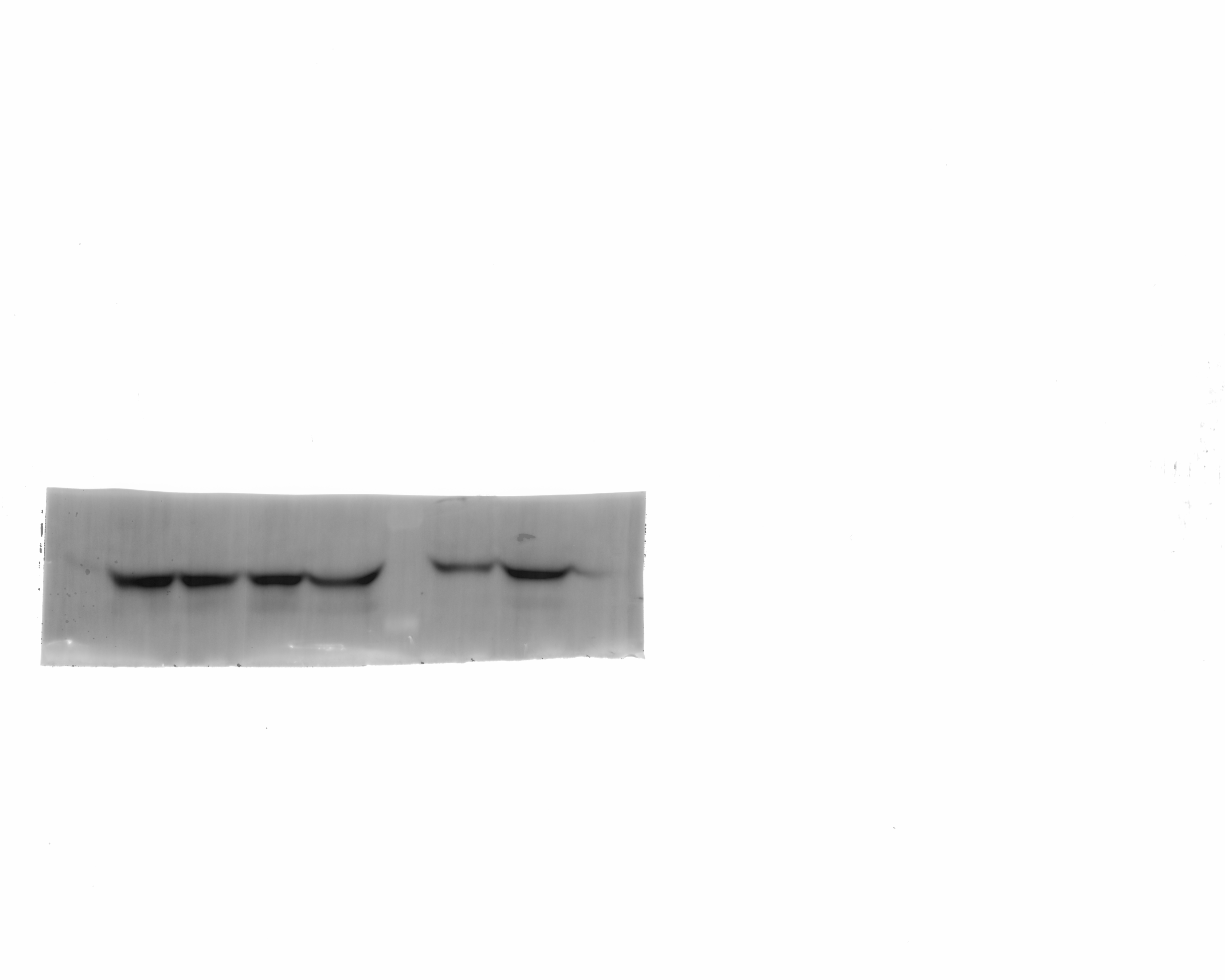

Supplement: Figure 1—figure supplement 2—source data 2. [file elife-104011-fig1-figsupp2-data2.zip › Figure 1. figure supplement 2-Source data 2/Source data Figure 1. figure supplement 2A Pgk1.tif]

Fig 1 Fig Supplement 2B

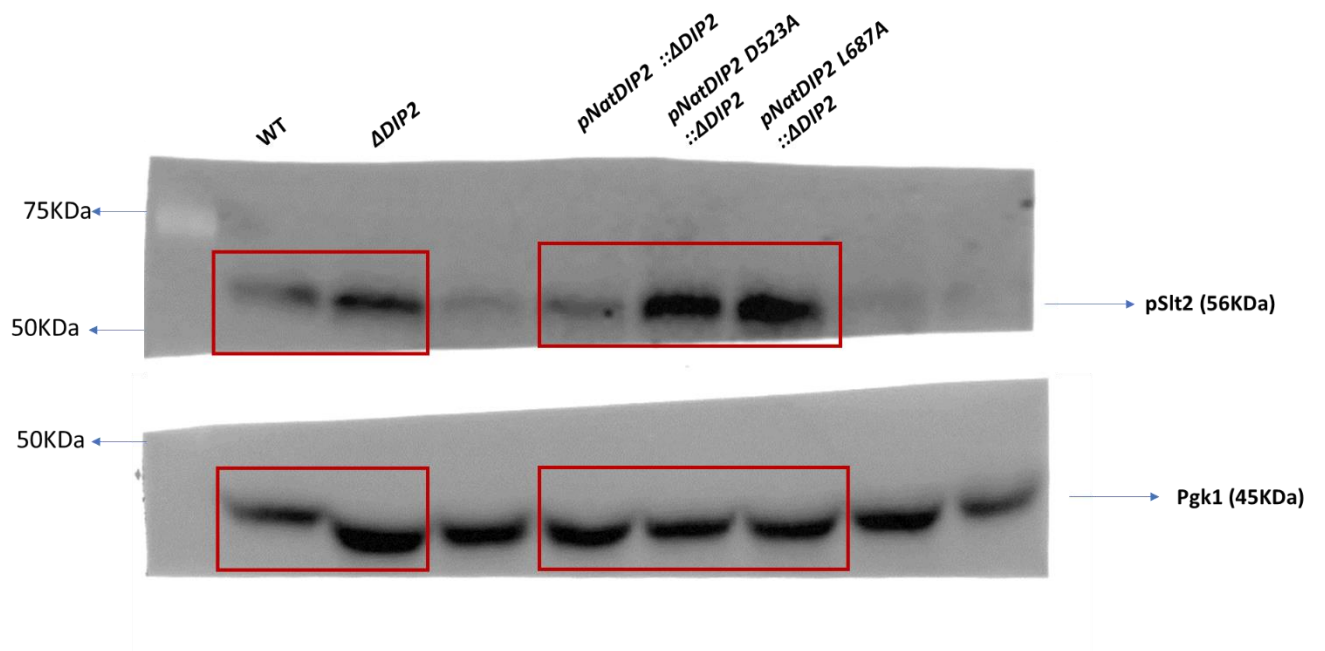

Whole blot was cut into 2 parts and probed for pSlt2 (M.W 56KDa) and Pgk1 (M.W. 45KDa)

Supplement: Figure 1—figure supplement 2—source data 3. [file elife-104011-fig1-figsupp2-data3.zip › Figure 1. figure supplement 2-Source data 3/Related to Figure 1. figure supplement 2B.pdf]

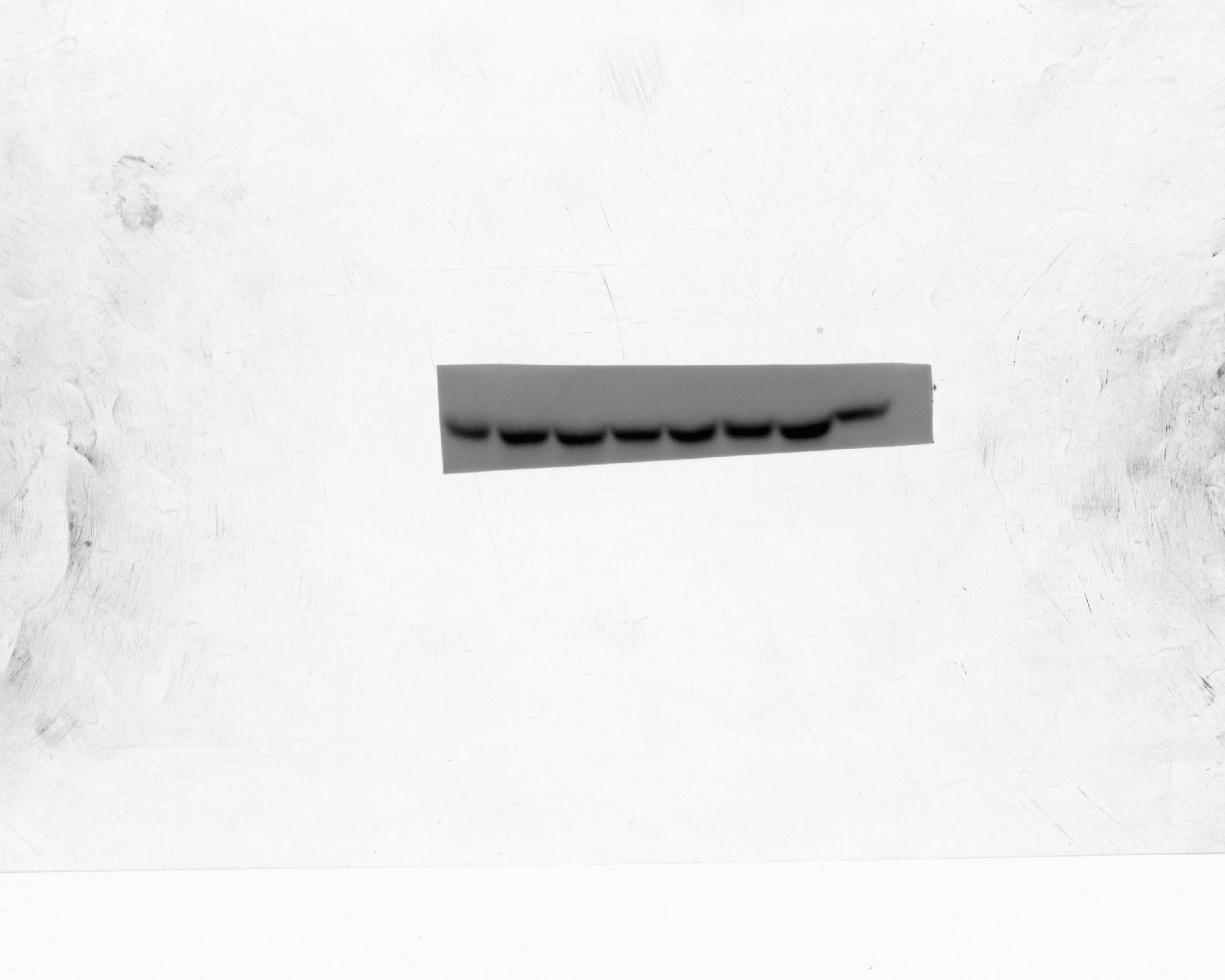

Supplement: Figure 1—figure supplement 2—source data 4. [file elife-104011-fig1-figsupp2-data4.zip › Figure 1. figure supplement 2-Source data 4/Related to Figure 1. figure supplement 2B Pgk1.tif]

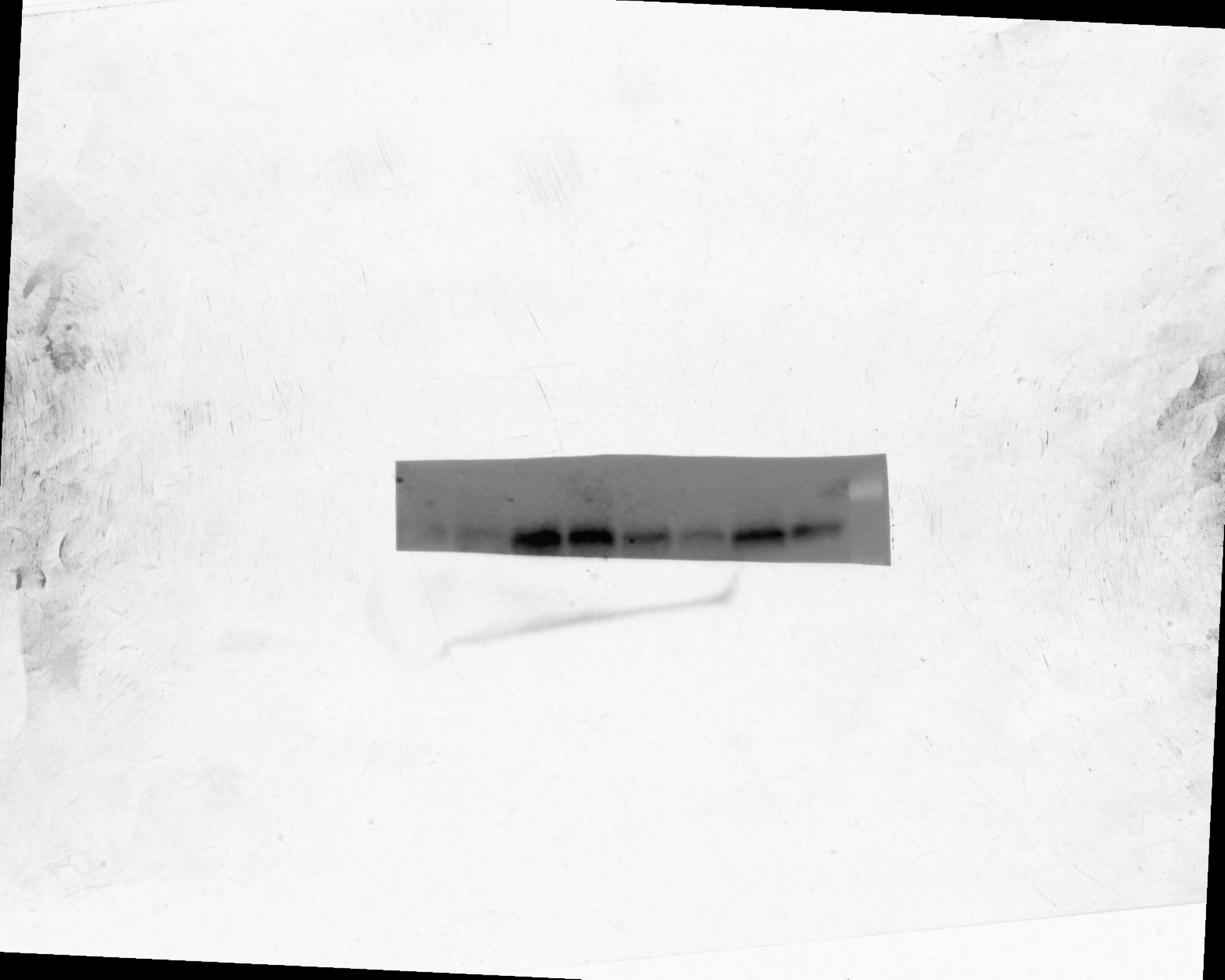

Supplement: Figure 1—figure supplement 2—source data 4. [file elife-104011-fig1-figsupp2-data4.zip › Figure 1. figure supplement 2-Source data 4/Related to Figure 1. figure supplement 2B pSlt2.tif]

Figure 2-source data 2

Figure 2C

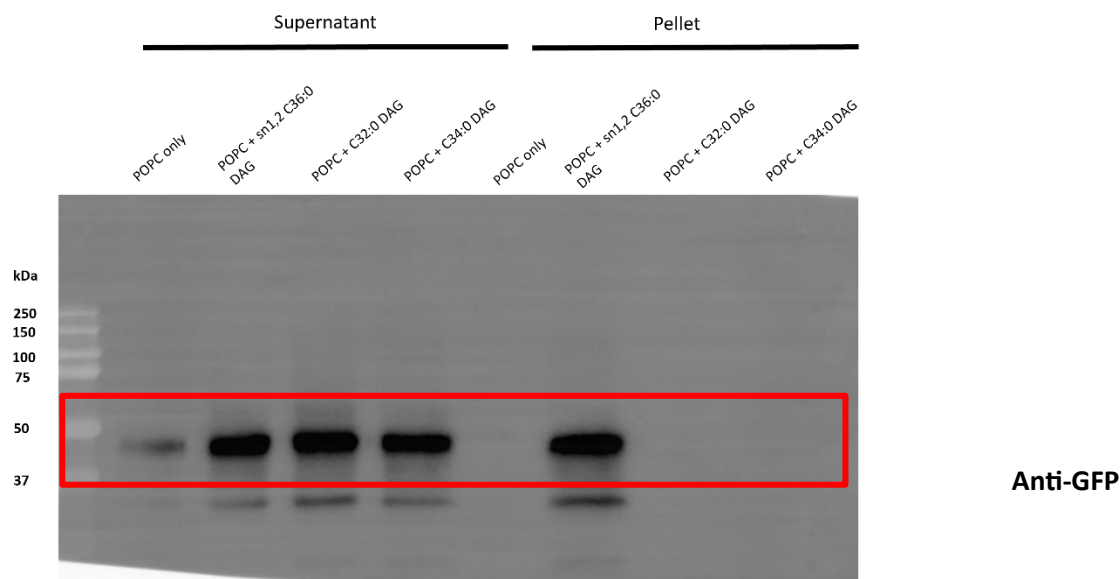

Figure 2D

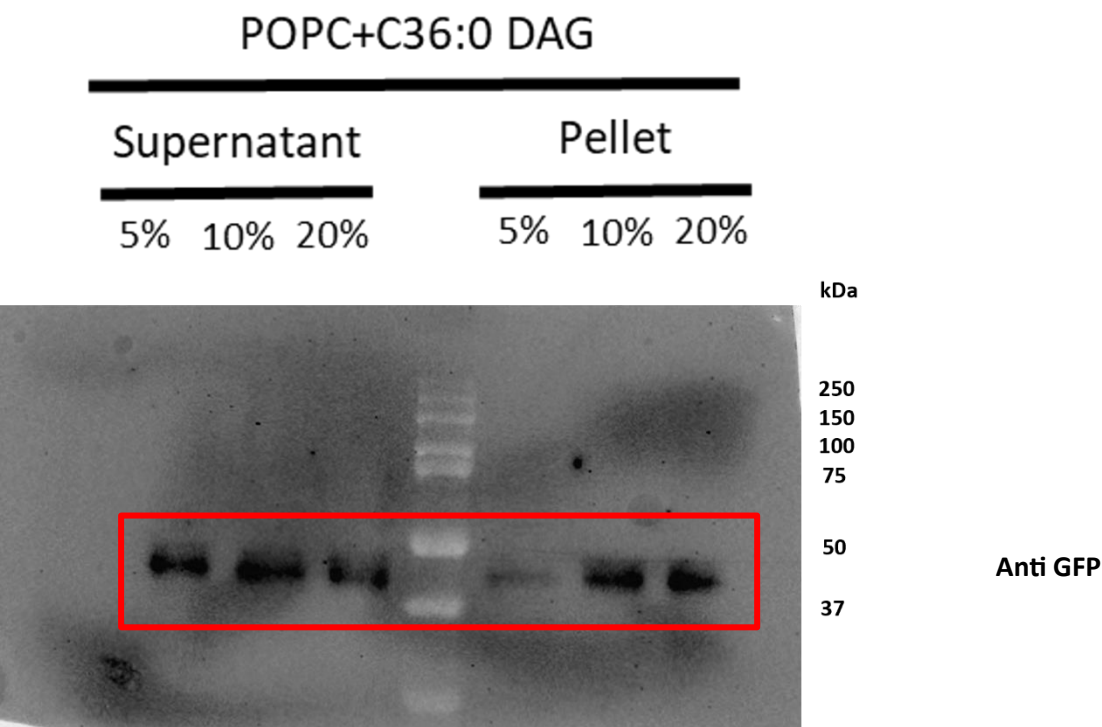

**Figure 2E**

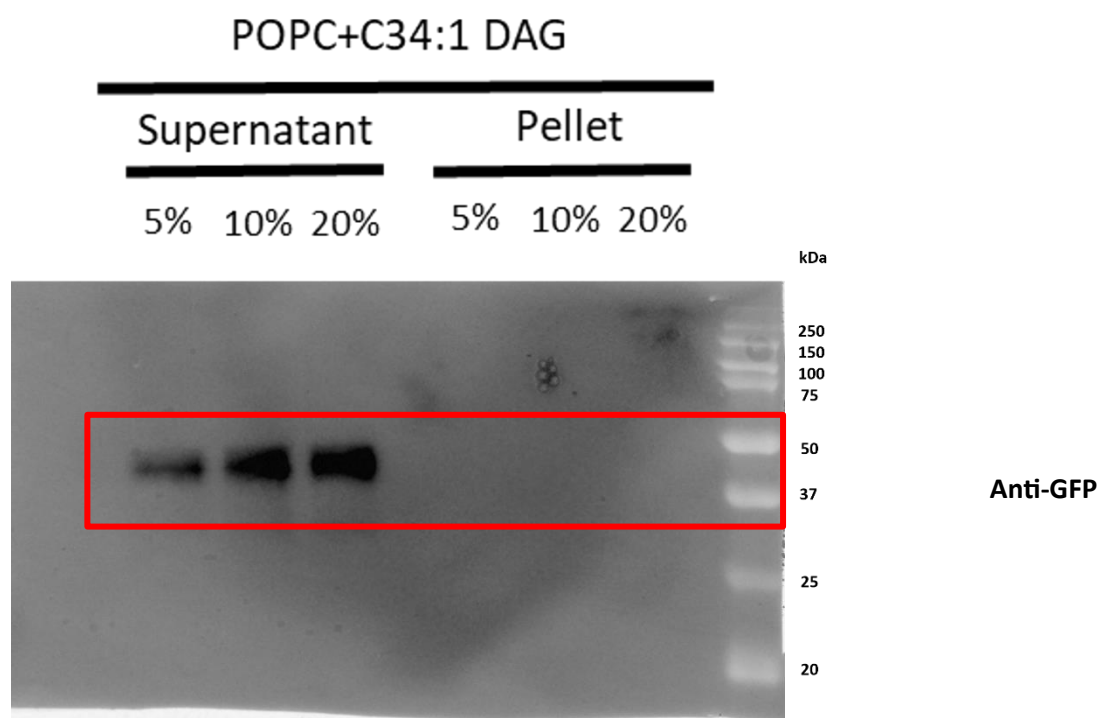

Supplement: Figure 2—source data 2. — PDF file containing original western blots for Figure 2C–E, indicating the relevant bands. [file elife-104011-fig2-data2.zip › Figure 2-source data 2/Figure 2-source data 2.pdf]

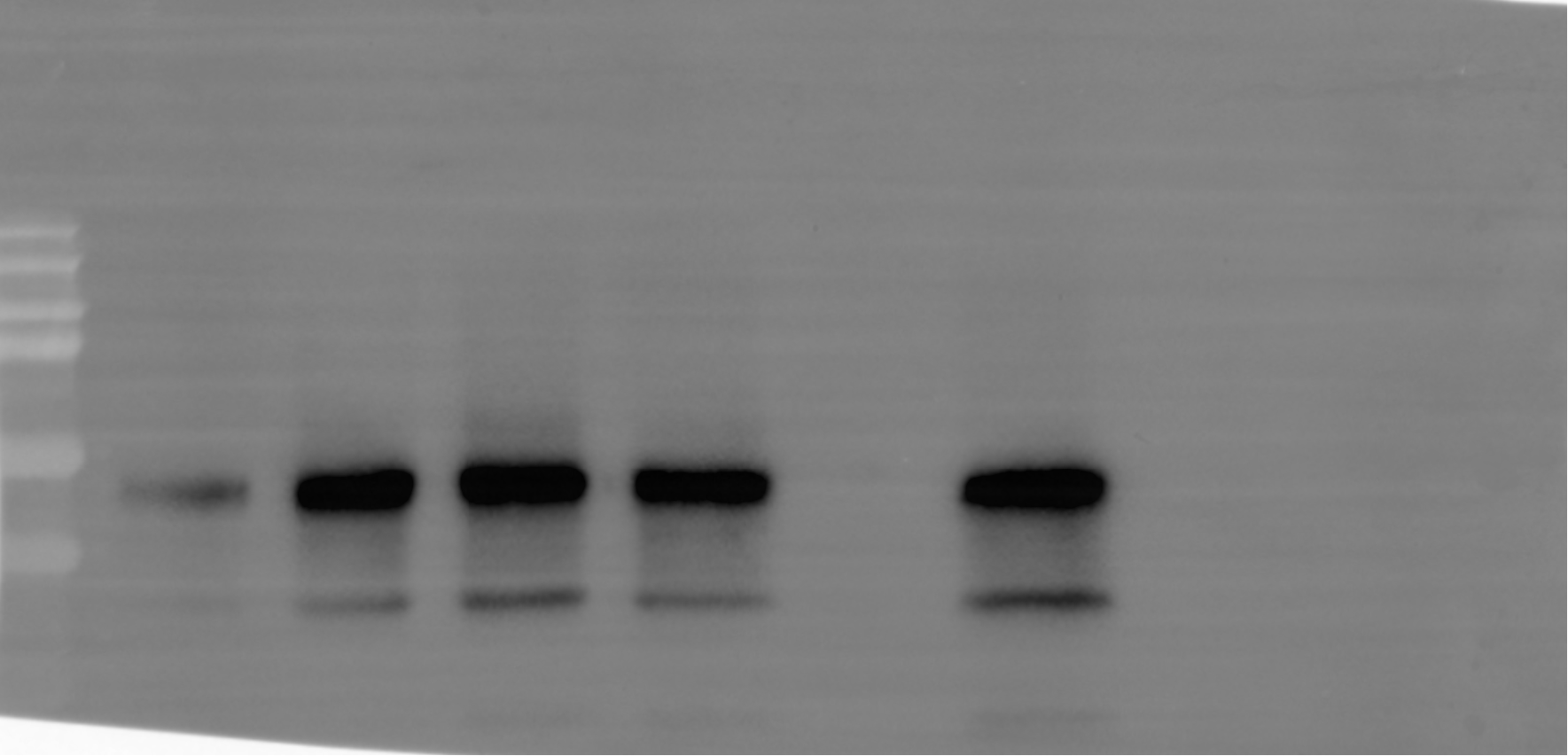

Supplement: Figure 2—source data 3. — Original files for western blot analysis displayed in Figure 2C–E. [file elife-104011-fig2-data3.zip › Figure 2-source data 3/Related to Fig 2C.tif]

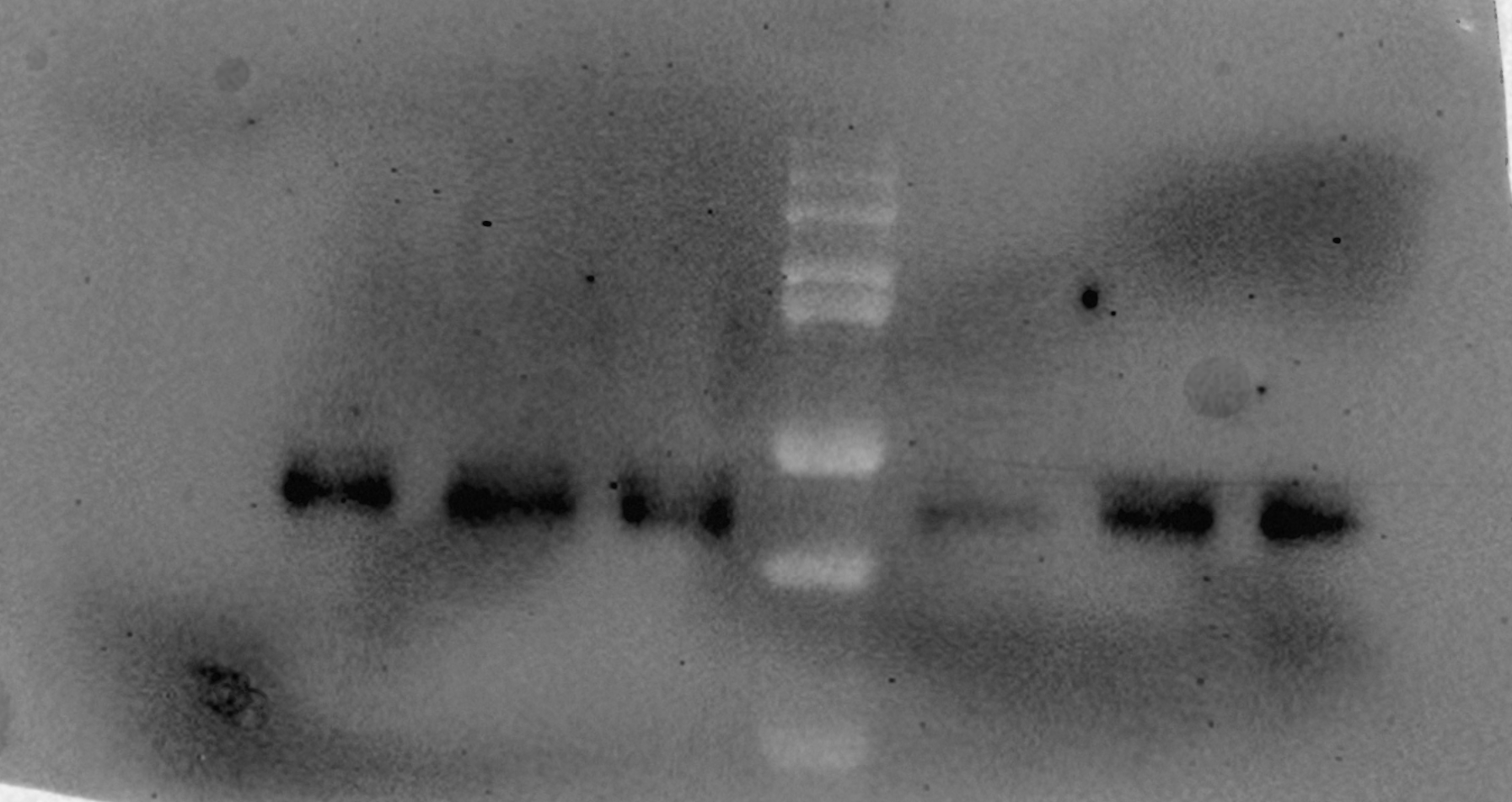

Supplement: Figure 2—source data 3. — Original files for western blot analysis displayed in Figure 2C–E. [file elife-104011-fig2-data3.zip › Figure 2-source data 3/Related to Fig 2D.tif]

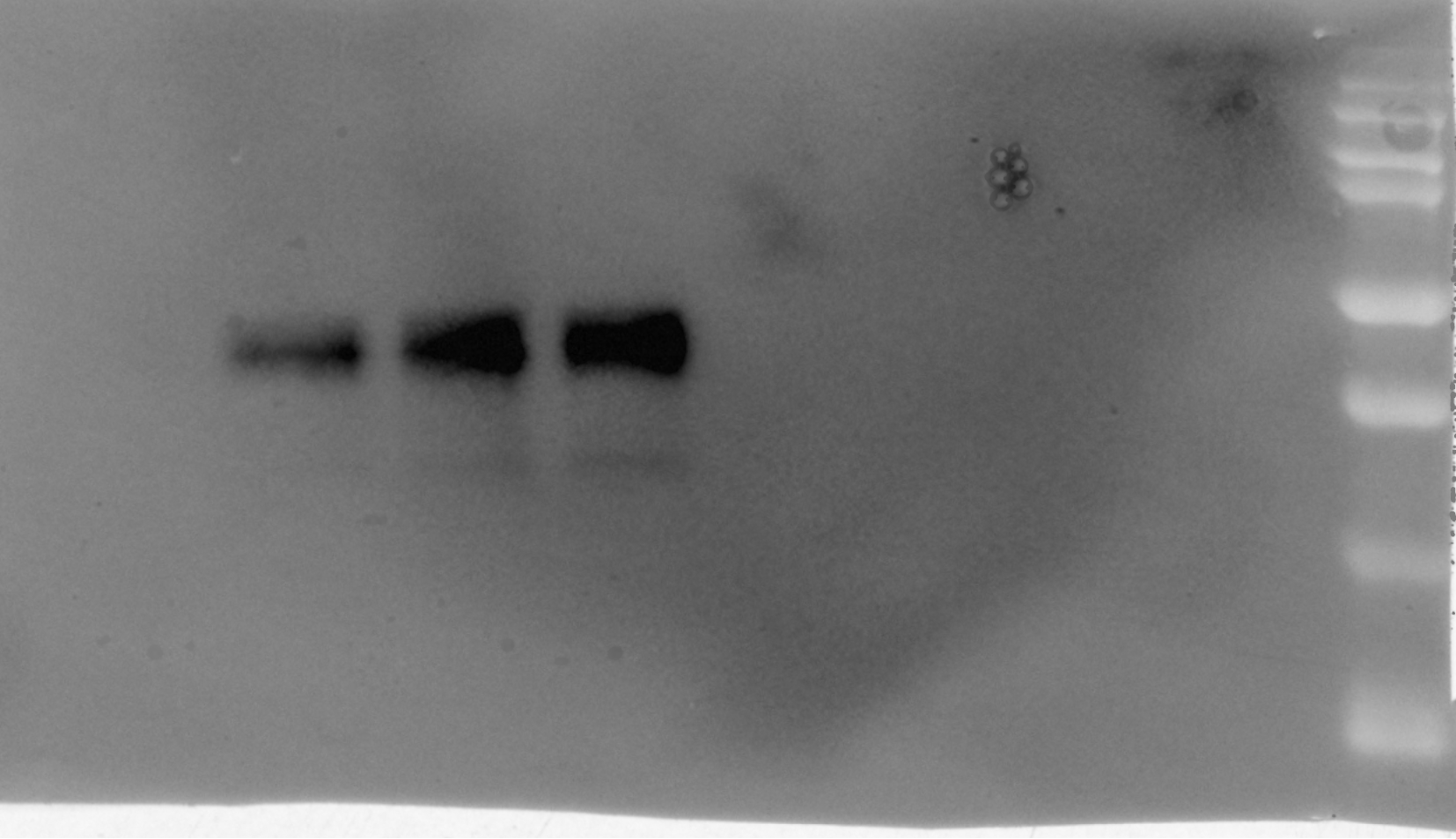

Supplement: Figure 2—source data 3. — Original files for western blot analysis displayed in Figure 2C–E. [file elife-104011-fig2-data3.zip › Figure 2-source data 3/Related to Fig 2E.tif]

**Figure 2. figure supplement 1 A**

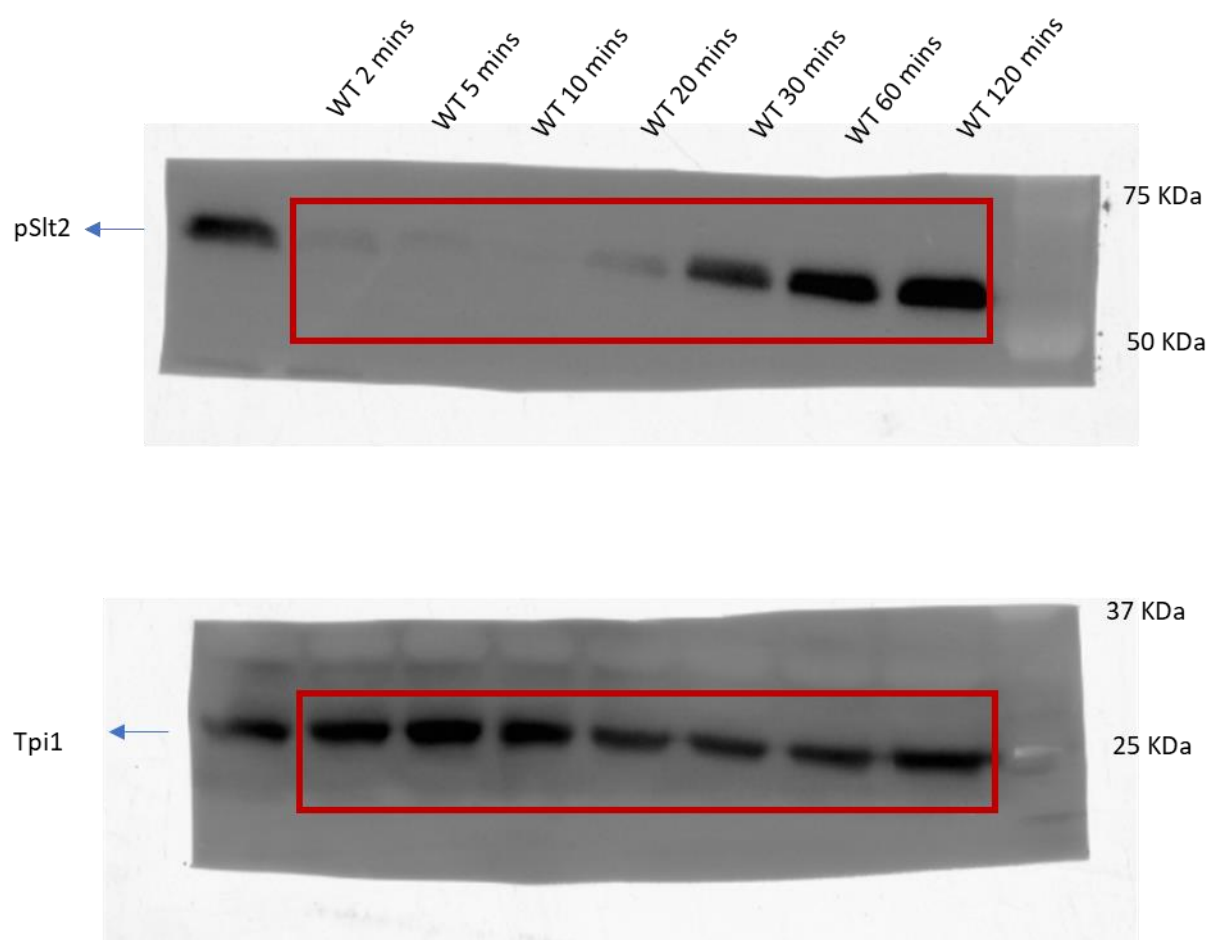

Supplement: Figure 2—figure supplement 1—source data 1. [file elife-104011-fig2-figsupp1-data1.zip › Figure 2. figure supplement 1 - Source data 1/Related to Source data Figure 2. figure supplement 1A.pdf]

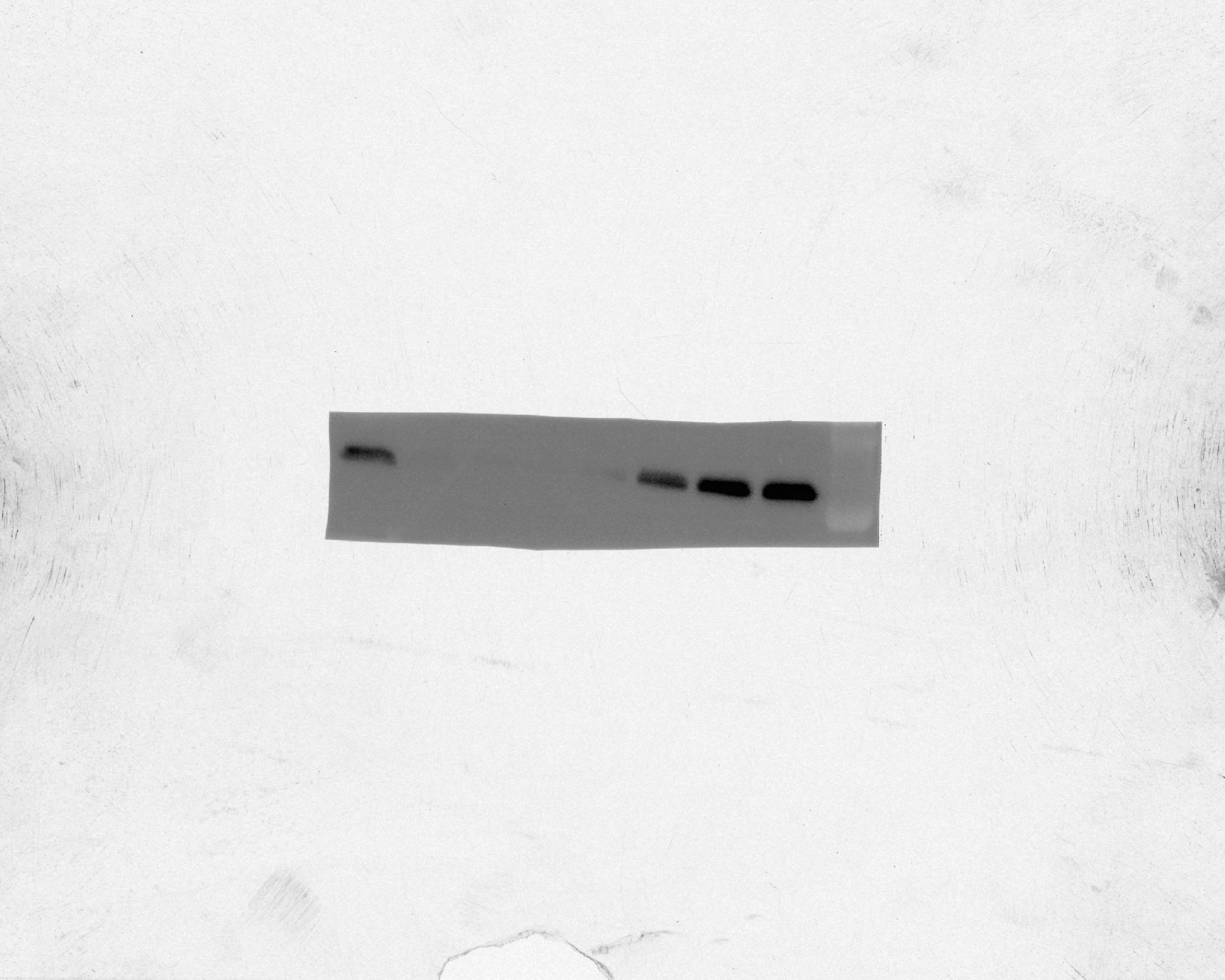

Supplement: Figure 2—figure supplement 1—source data 2. [file elife-104011-fig2-figsupp1-data2.zip › Figure 2. figure supplement 1-Source data 2/Related to Source data Figure 2. figure supplement 1A pSlt2.tif]

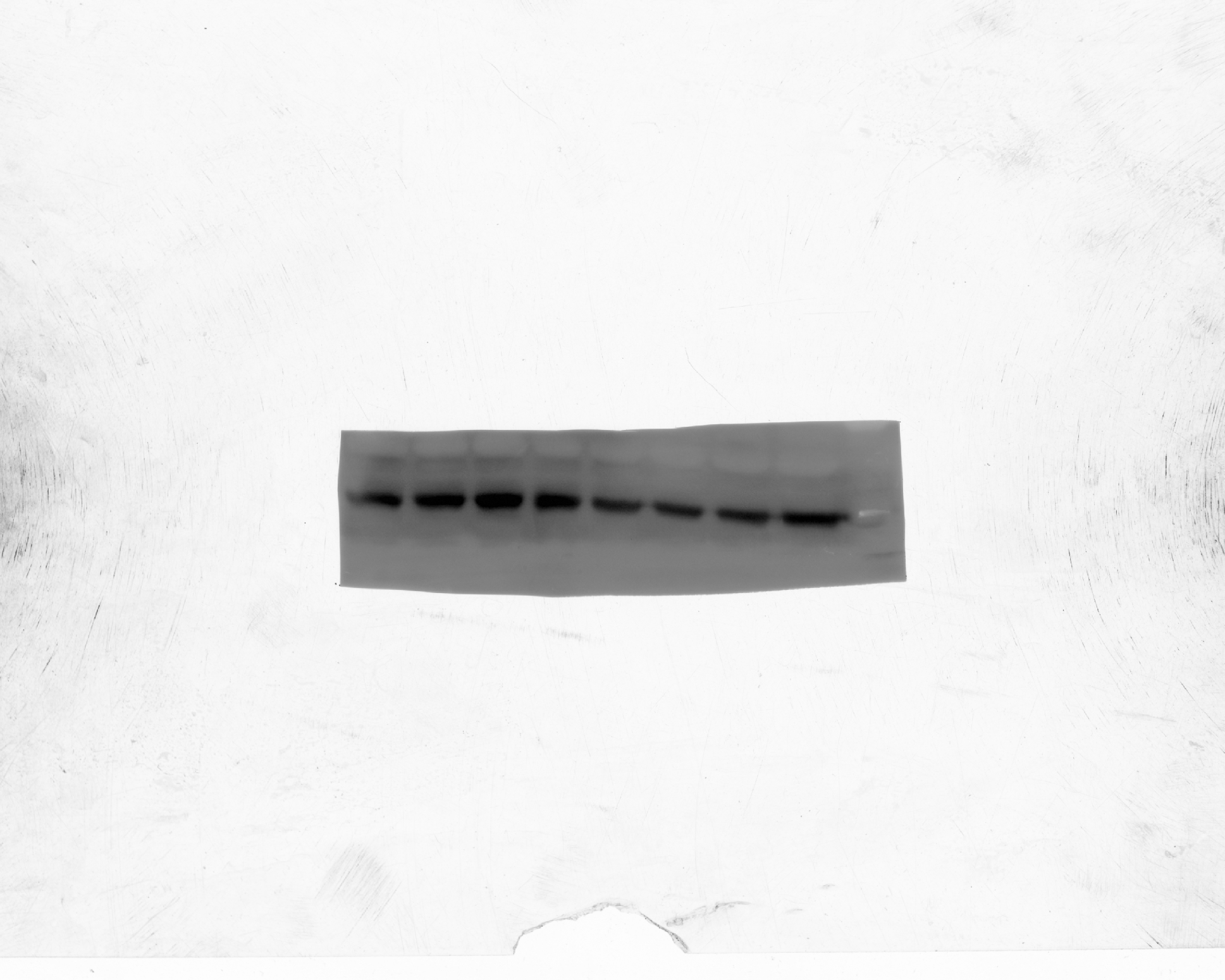

Supplement: Figure 2—figure supplement 1—source data 2. [file elife-104011-fig2-figsupp1-data2.zip › Figure 2. figure supplement 1-Source data 2/Related to Source data Figure 2. figure supplement 1A Tpi1.tif]

**Figure 2. figure supplement 3A**

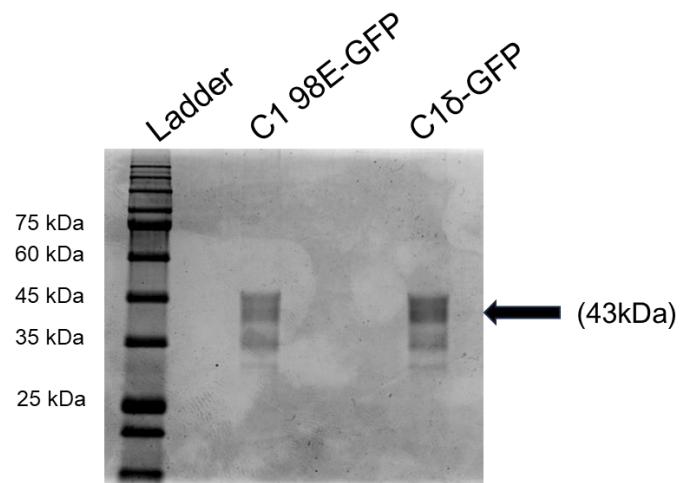

**Figure 2. figure supplement 3B**

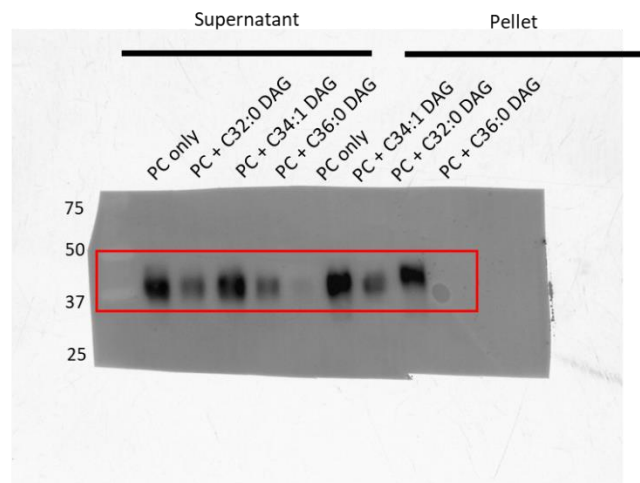

Figure 2. figure supplement 3C

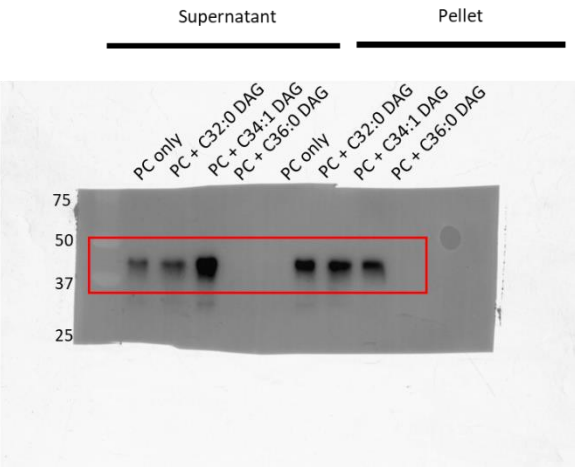

Supplement: Figure 2—figure supplement 3—source data 1. [file elife-104011-fig2-figsupp3-data1.zip › Figure 2—figure supplement 3—source data 1.pdf]

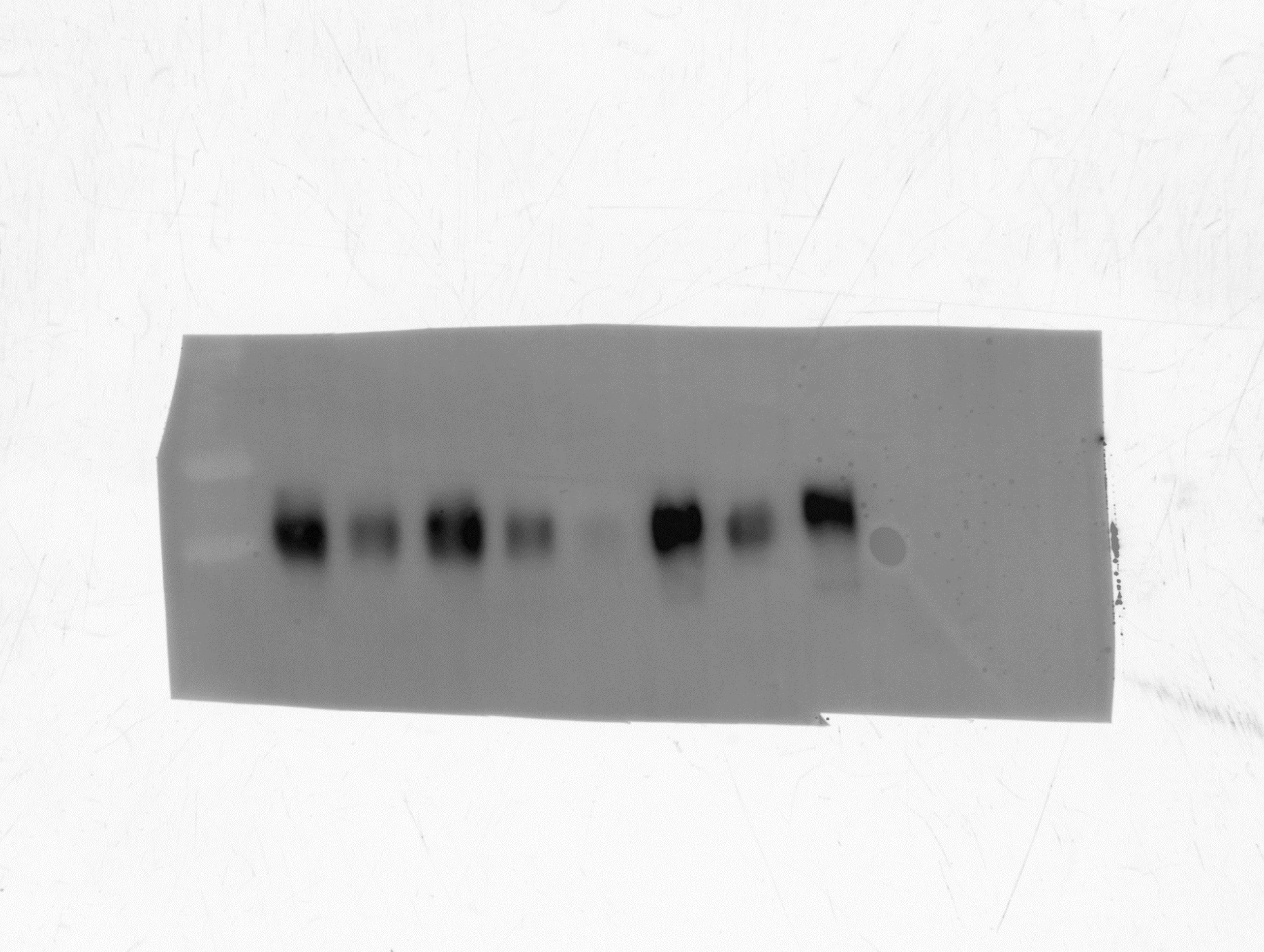

Supplement: Figure 2—figure supplement 3—source data 2. [file elife-104011-fig2-figsupp3-data2.zip › Figure 2. figure supplement 3 - Source data 2/Figure 2. figure supplement 3B.tif]

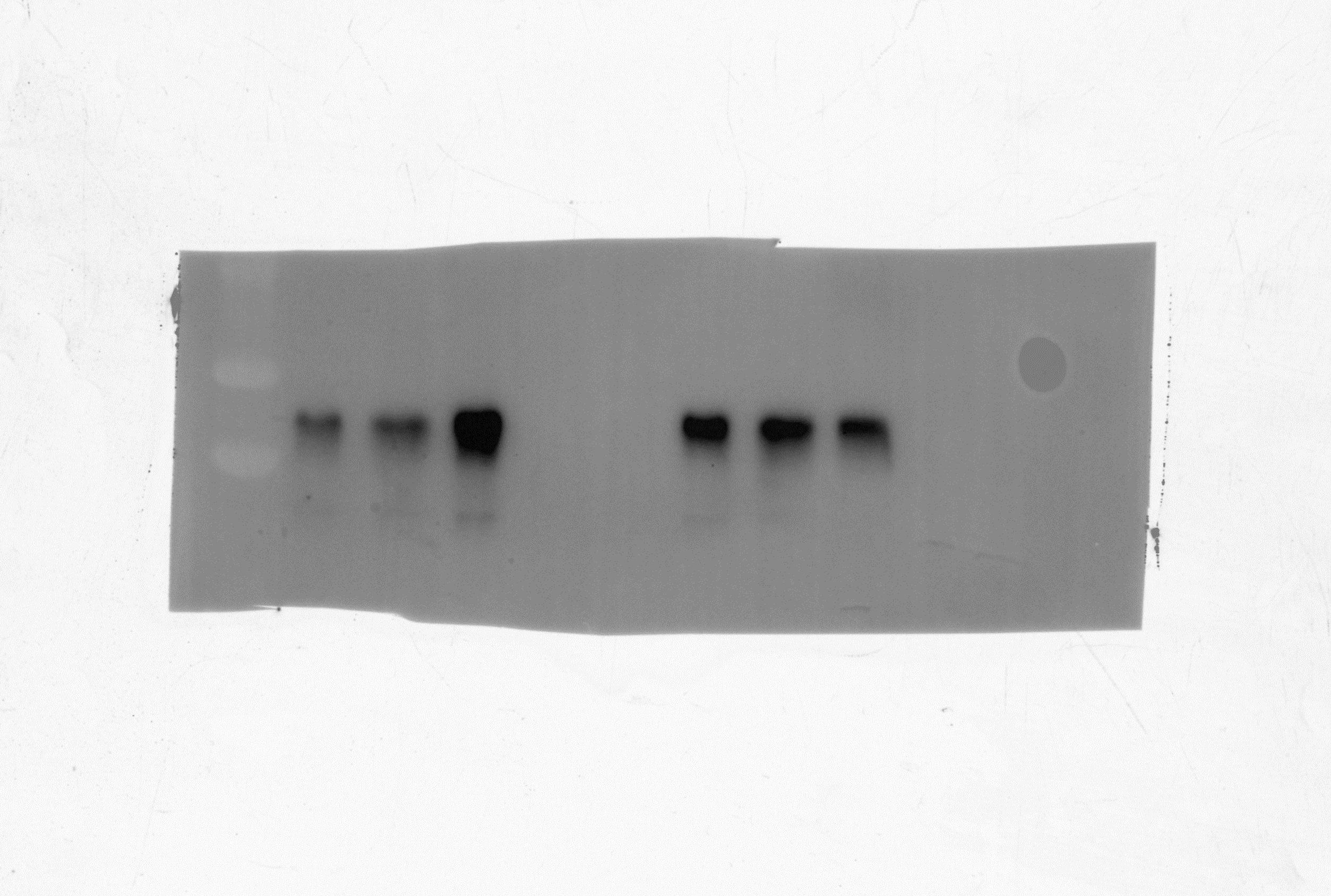

Supplement: Figure 2—figure supplement 3—source data 2. [file elife-104011-fig2-figsupp3-data2.zip › Figure 2. figure supplement 3 - Source data 2/Figure 2. figure supplement 3C.tif]

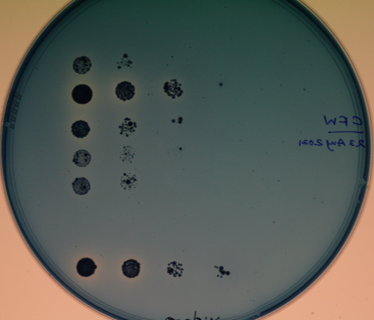

Supplement: Figure 3—source data 3. — Original files for spot assay plate images displayed in Figure 3D. [file elife-104011-fig3-data3.zip › Figure 3- source data 3/Related to 3D CFW 1.tif]

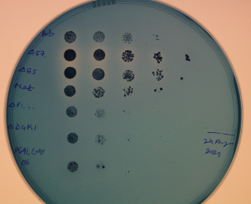

Supplement: Figure 3—source data 3. — Original files for spot assay plate images displayed in Figure 3D. [file elife-104011-fig3-data3.zip › Figure 3- source data 3/Related to 3D CFW 2.tif]

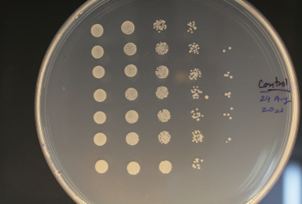

Supplement: Figure 3—source data 3. — Original files for spot assay plate images displayed in Figure 3D. [file elife-104011-fig3-data3.zip › Figure 3- source data 3/Related to 3D control 2.tif]

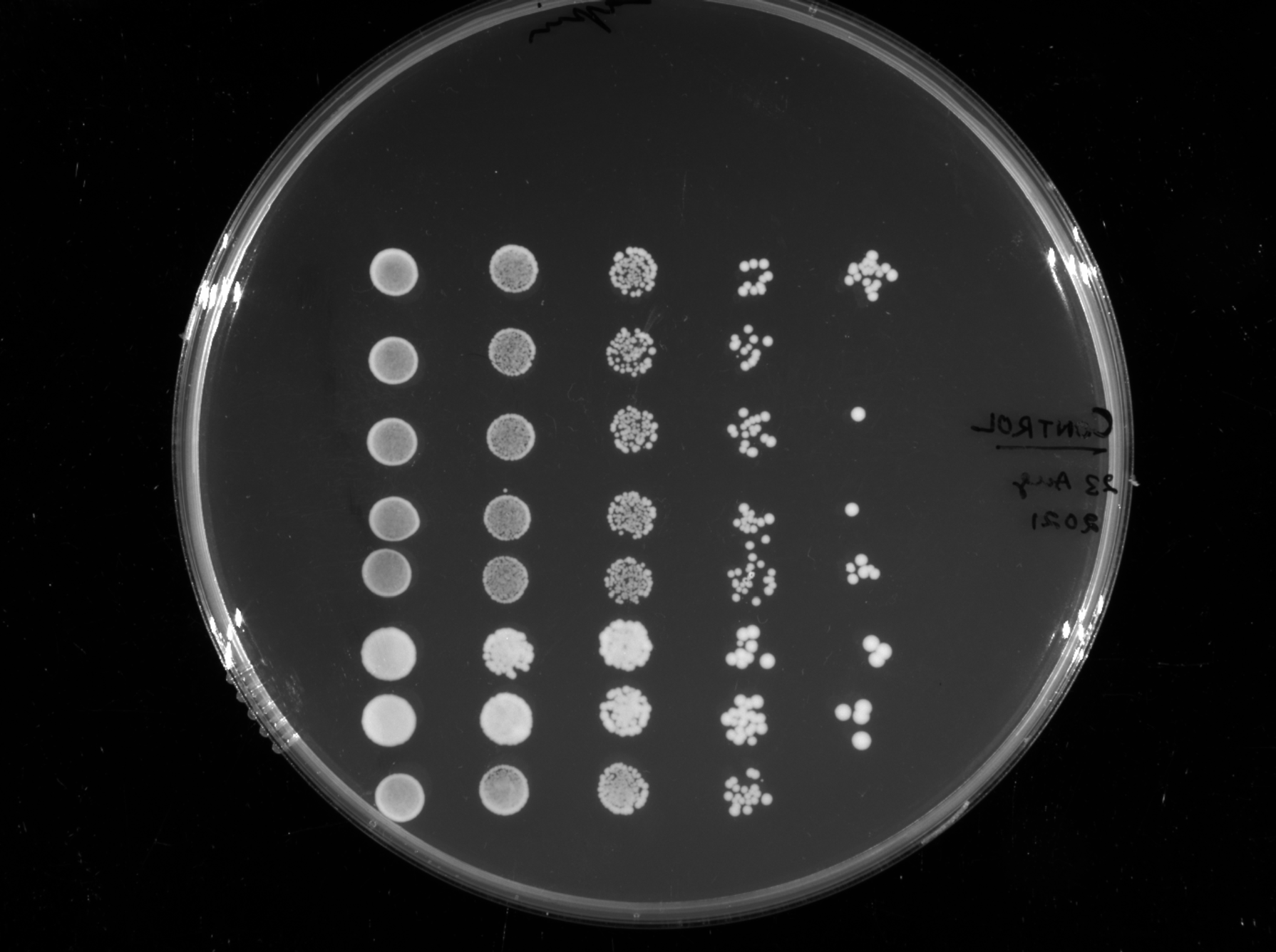

Supplement: Figure 3—source data 3. — Original files for spot assay plate images displayed in Figure 3D. [file elife-104011-fig3-data3.zip › Figure 3- source data 3/Related to 3D control.tif]

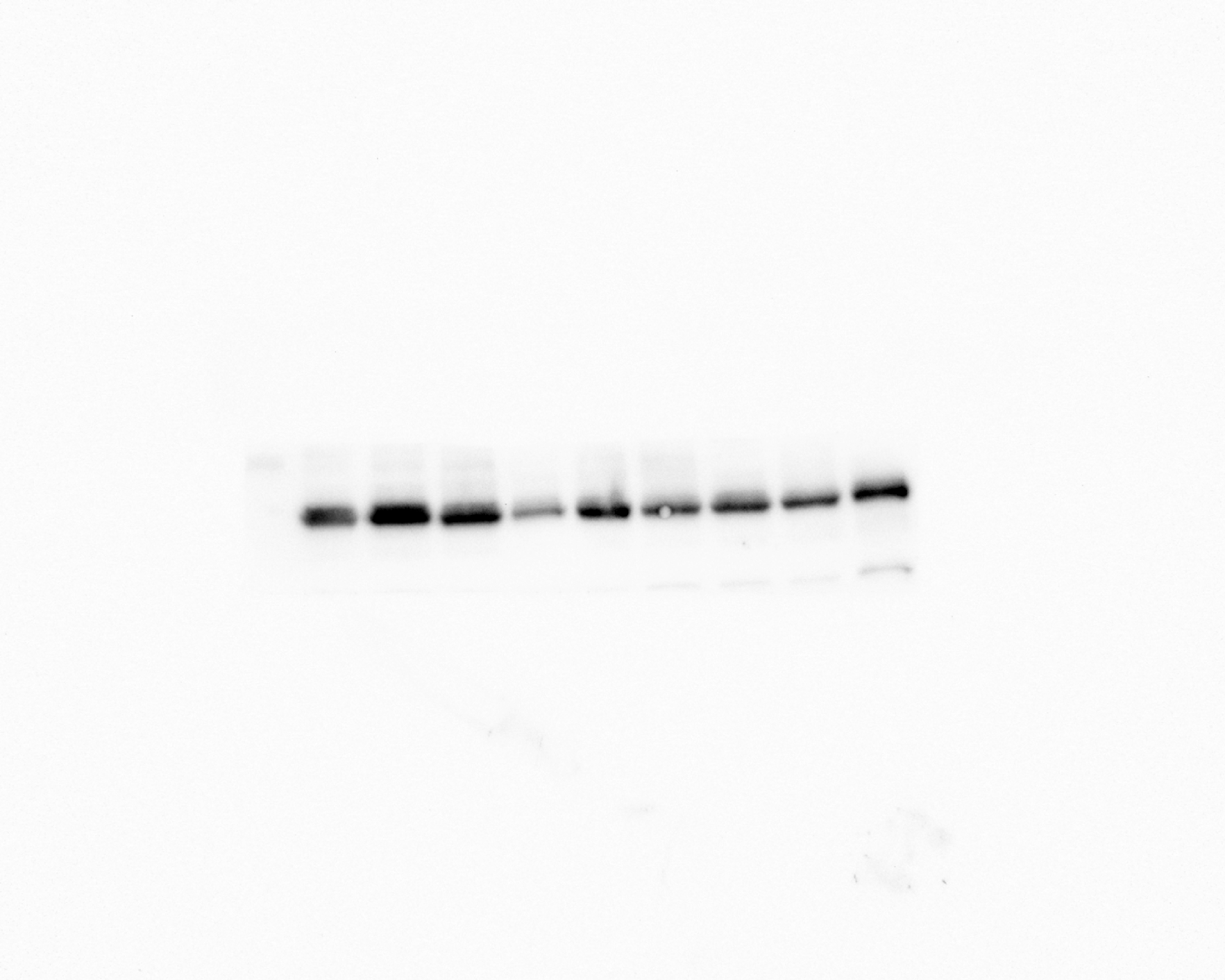

Supplement: Figure 3—source data 5. [file elife-104011-fig3-data5.zip › Figure 3- source data 5/Related to Fig 3E pSlt2.jpg]

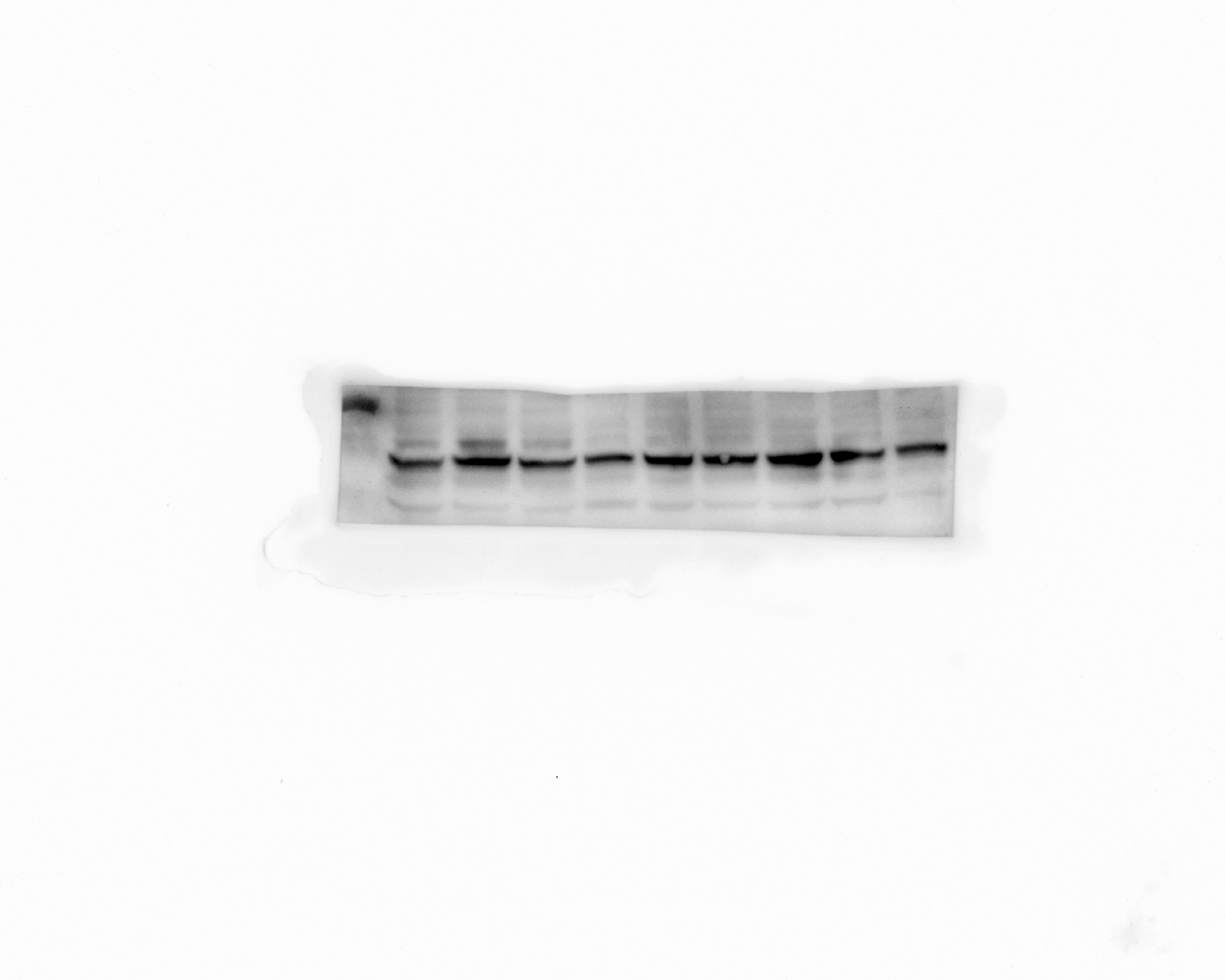

Supplement: Figure 3—source data 5. [file elife-104011-fig3-data5.zip › Figure 3- source data 5/Related to Fig 3E Slt2.tif]

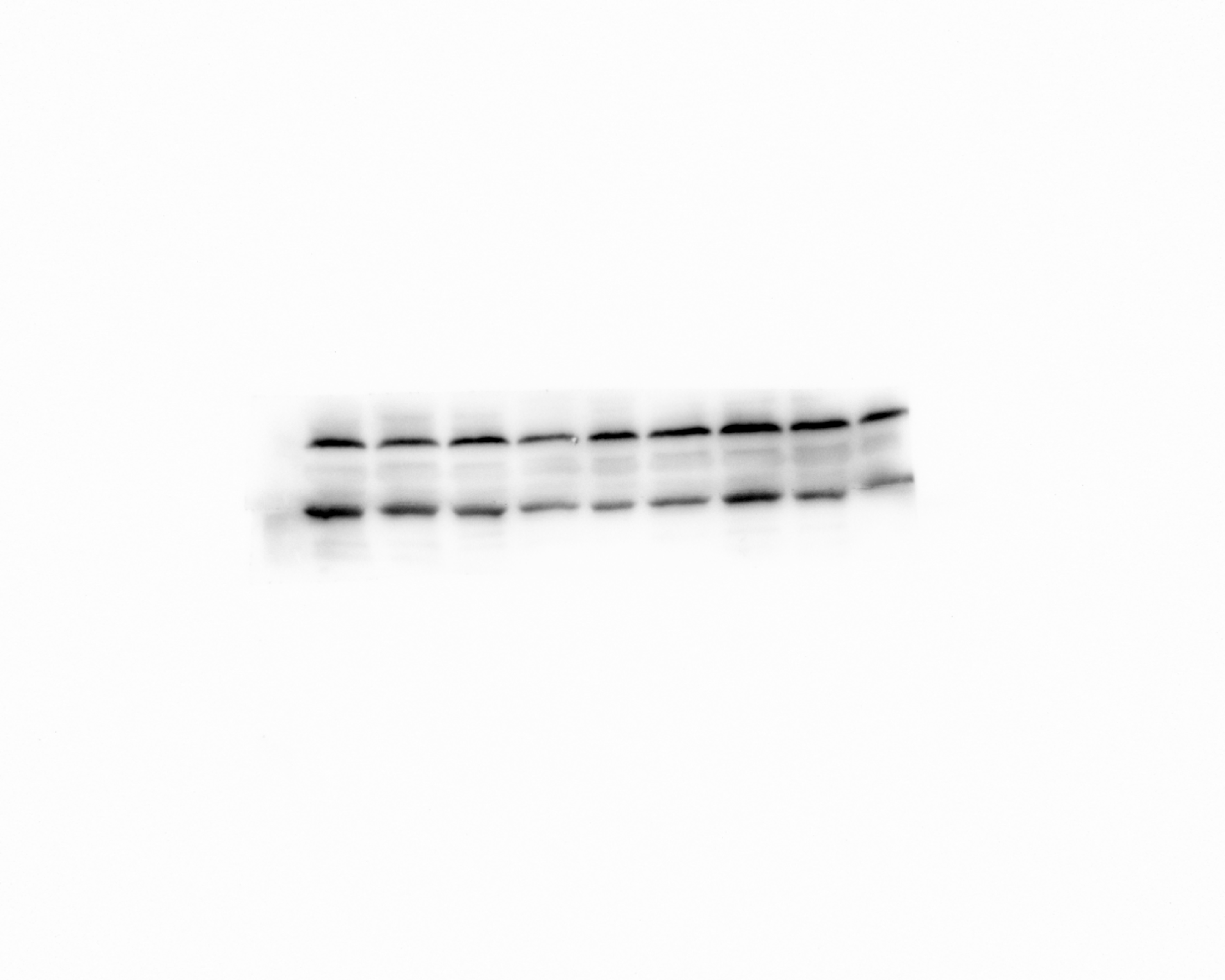

Supplement: Figure 3—source data 5. [file elife-104011-fig3-data5.zip › Figure 3- source data 5/Related to Fig 3E.tif]

Figure 3- source data 6

Spot assay for *LRO1 DGA1* DKO

Control

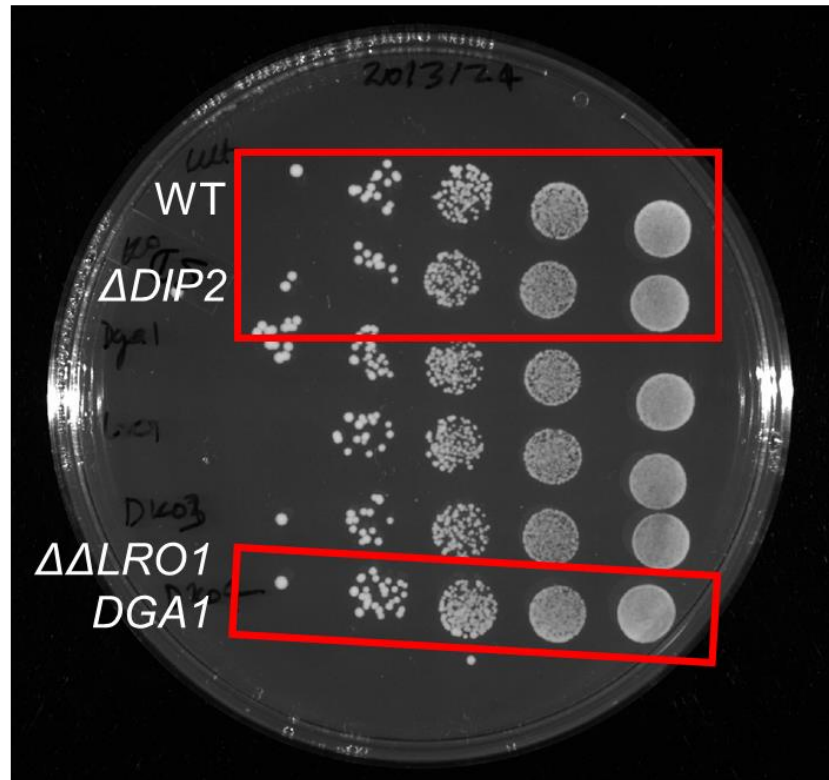

CW stress (CFW)

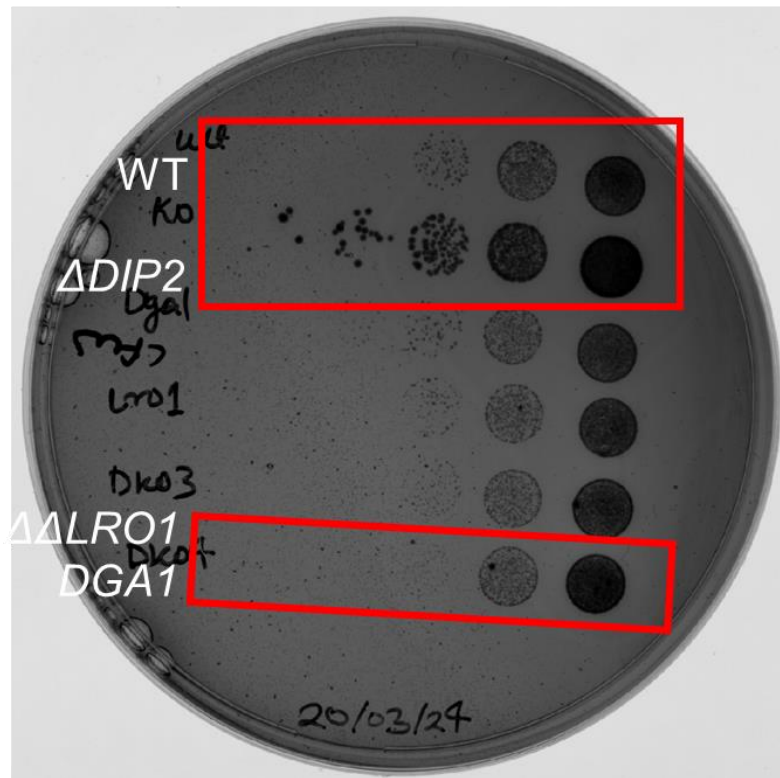

Supplement: Figure 3—source data 6. — PDF file containing original spot assay plate images for Figure 3G, indicating the relevant spots and treatments. [file elife-104011-fig3-data6.zip › Figure 3- source data 6/Figure 3- source data 6.pdf]

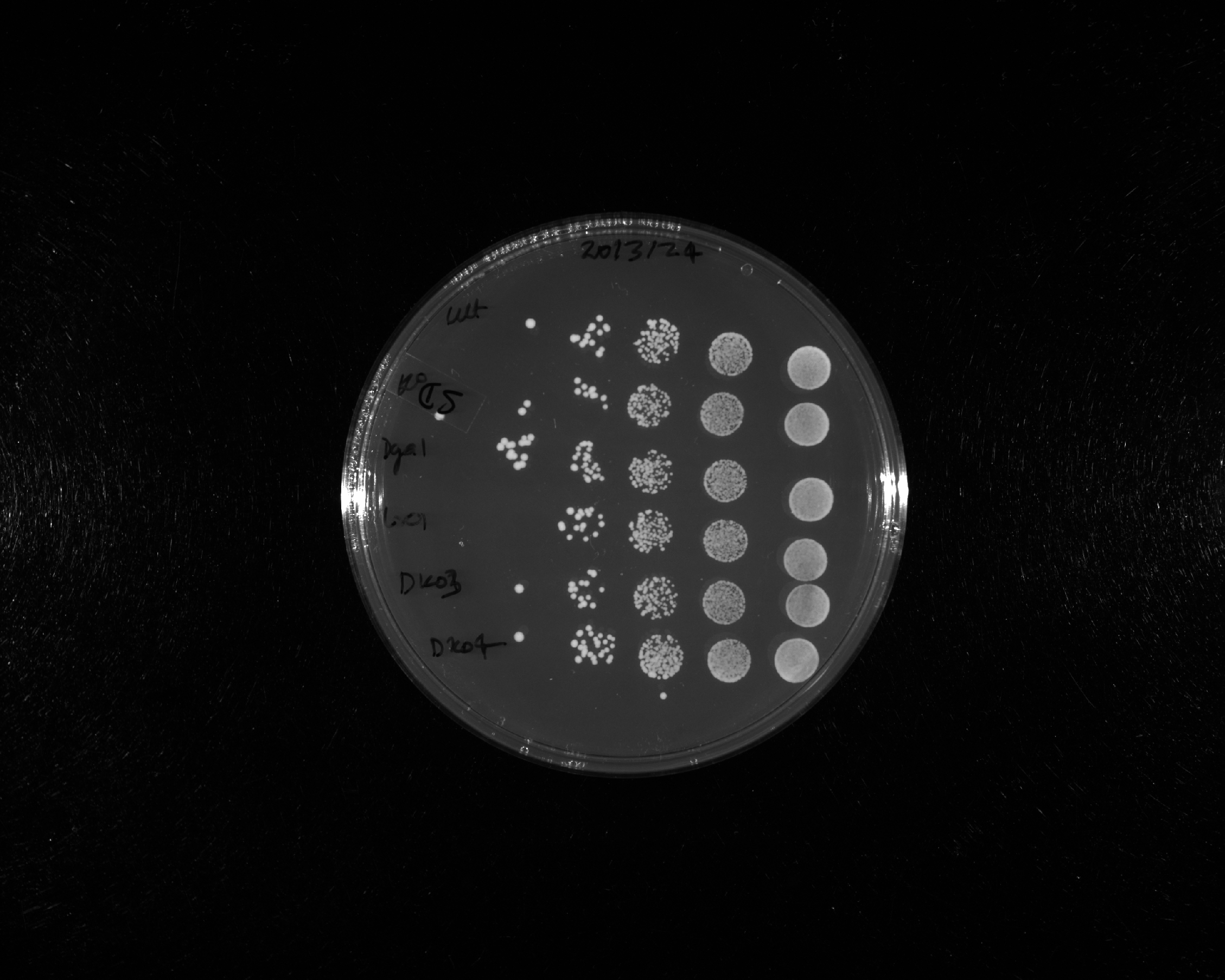

Supplement: Figure 3—source data 7. — Original files for spot assay plate images displayed in Figure 3G. [file elife-104011-fig3-data7.zip › Figure 3- source data 7/Related to Fig 3G control.tif]

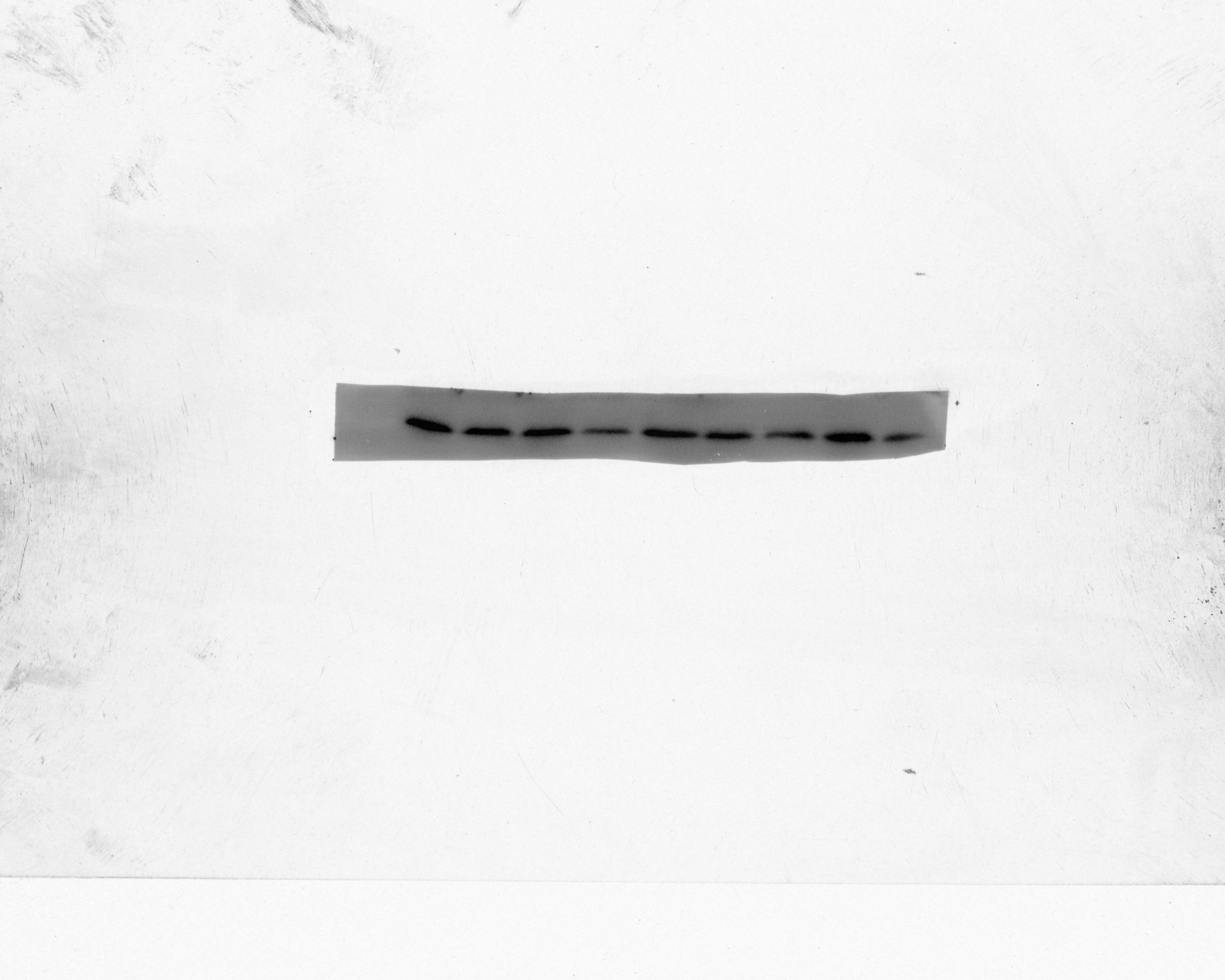

Supplement: Figure 3—source data 9. — Original files for western blot analysis displayed in Figure 3H. [file elife-104011-fig3-data9.zip › Figure 3- source data 9/Related to Fig 3H Gapdh.tif]

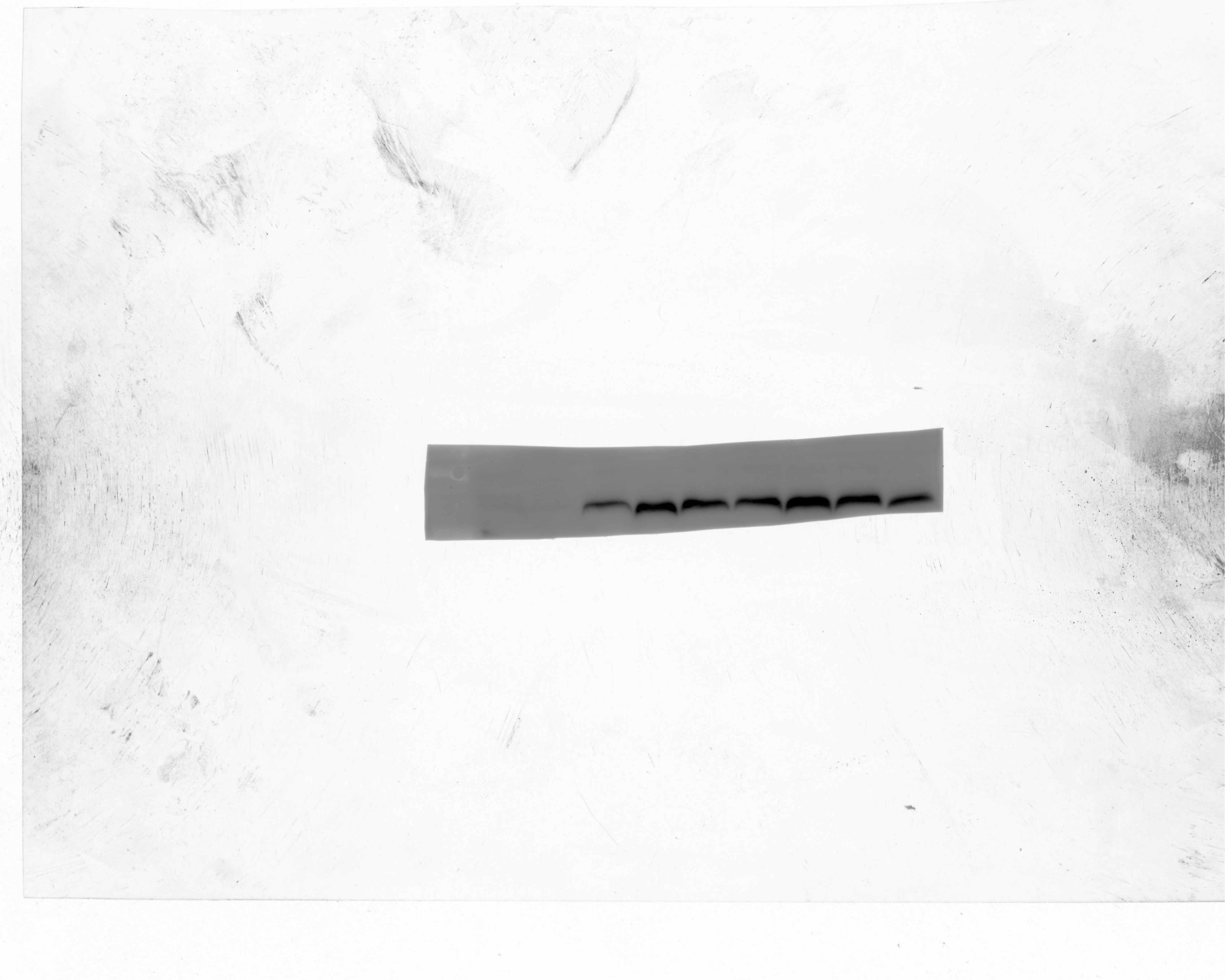

Supplement: Figure 3—source data 9. — Original files for western blot analysis displayed in Figure 3H. [file elife-104011-fig3-data9.zip › Figure 3- source data 9/Related to Fig 3H pSlt2.tif]

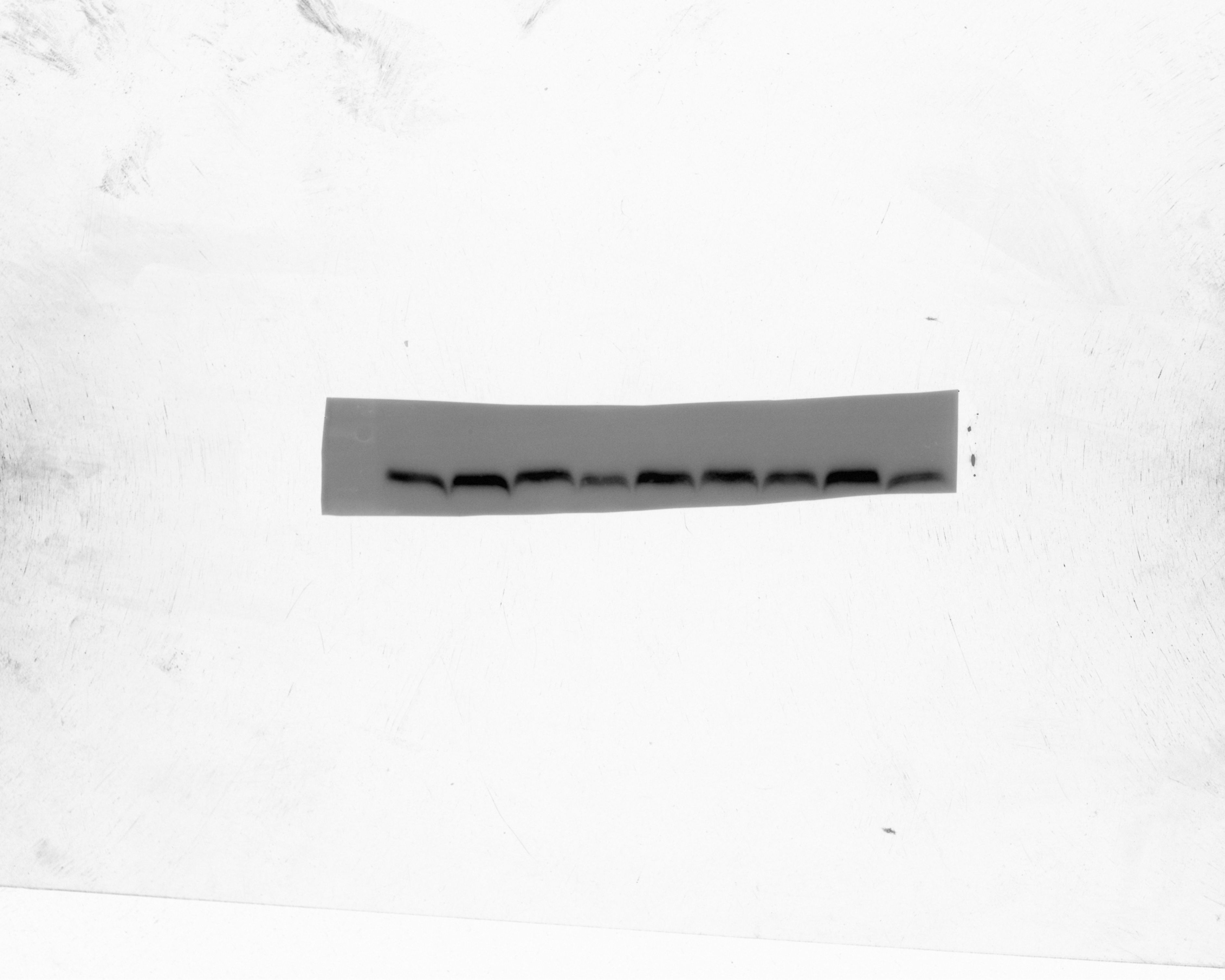

Supplement: Figure 3—source data 9. — Original files for western blot analysis displayed in Figure 3H. [file elife-104011-fig3-data9.zip › Figure 3- source data 9/Related to Fig 3H Slt2.tif]

Figure 3. figure supplement 1A

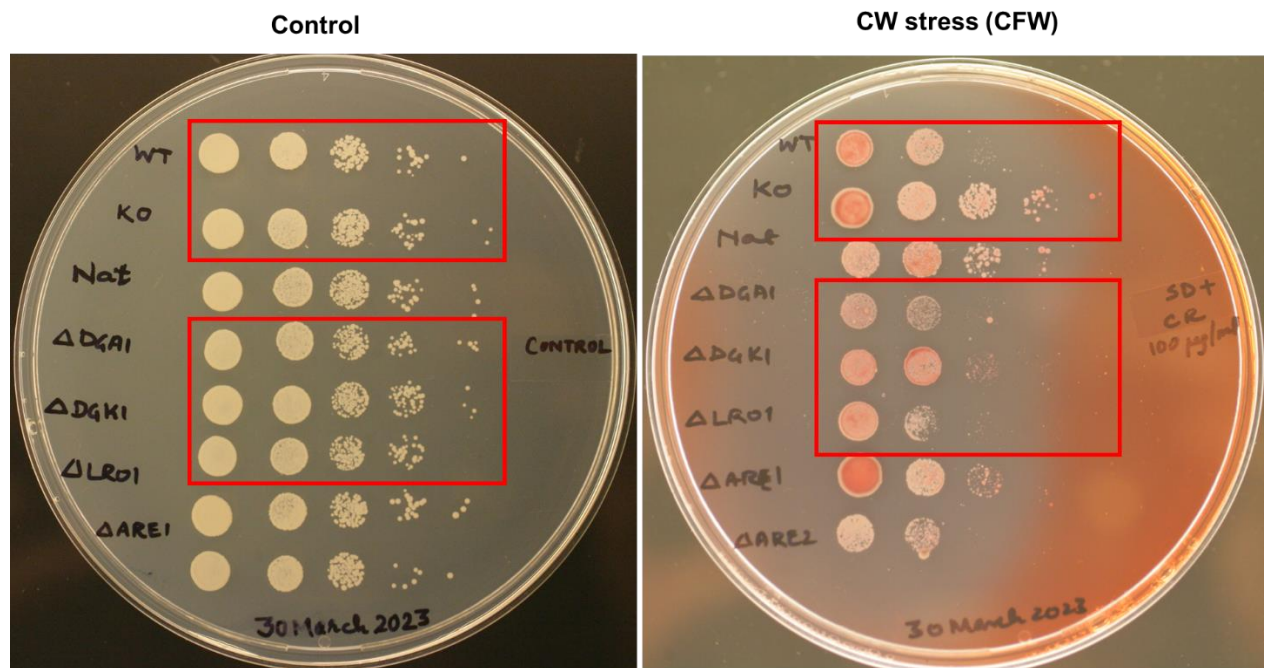

Supplement: Figure 3—figure supplement 1—source data 1. [file elife-104011-fig3-figsupp1-data1.zip › Figure 3 -figure supplement 1-source data 1/Figure 3. figure supplement 1A.pdf]

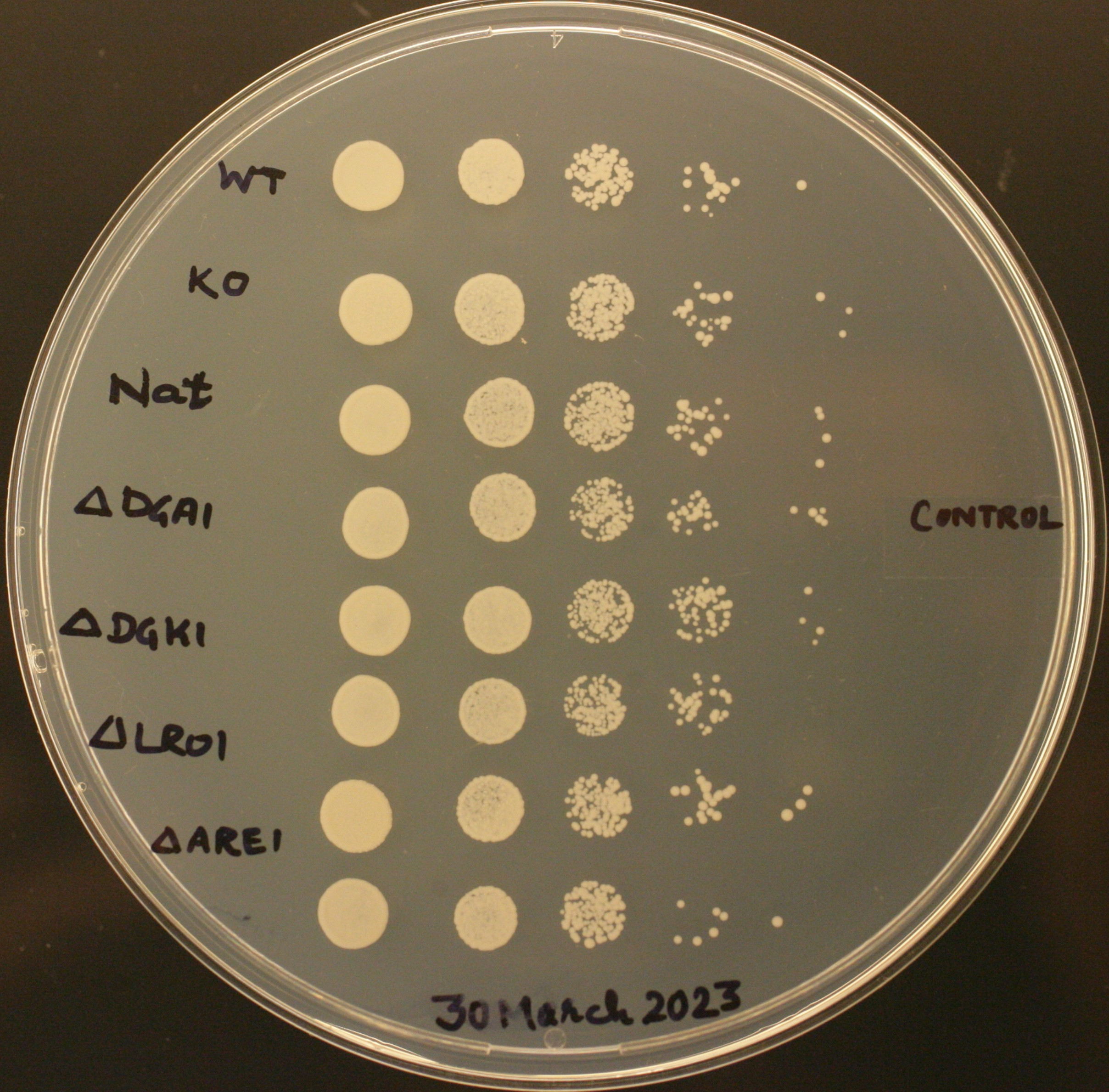

Supplement: Figure 3—figure supplement 1—source data 2. [file elife-104011-fig3-figsupp1-data2.zip › Figure 3. figure supplement 1- Source data 2/Figure 3. figure supplement 1A Control.tif]

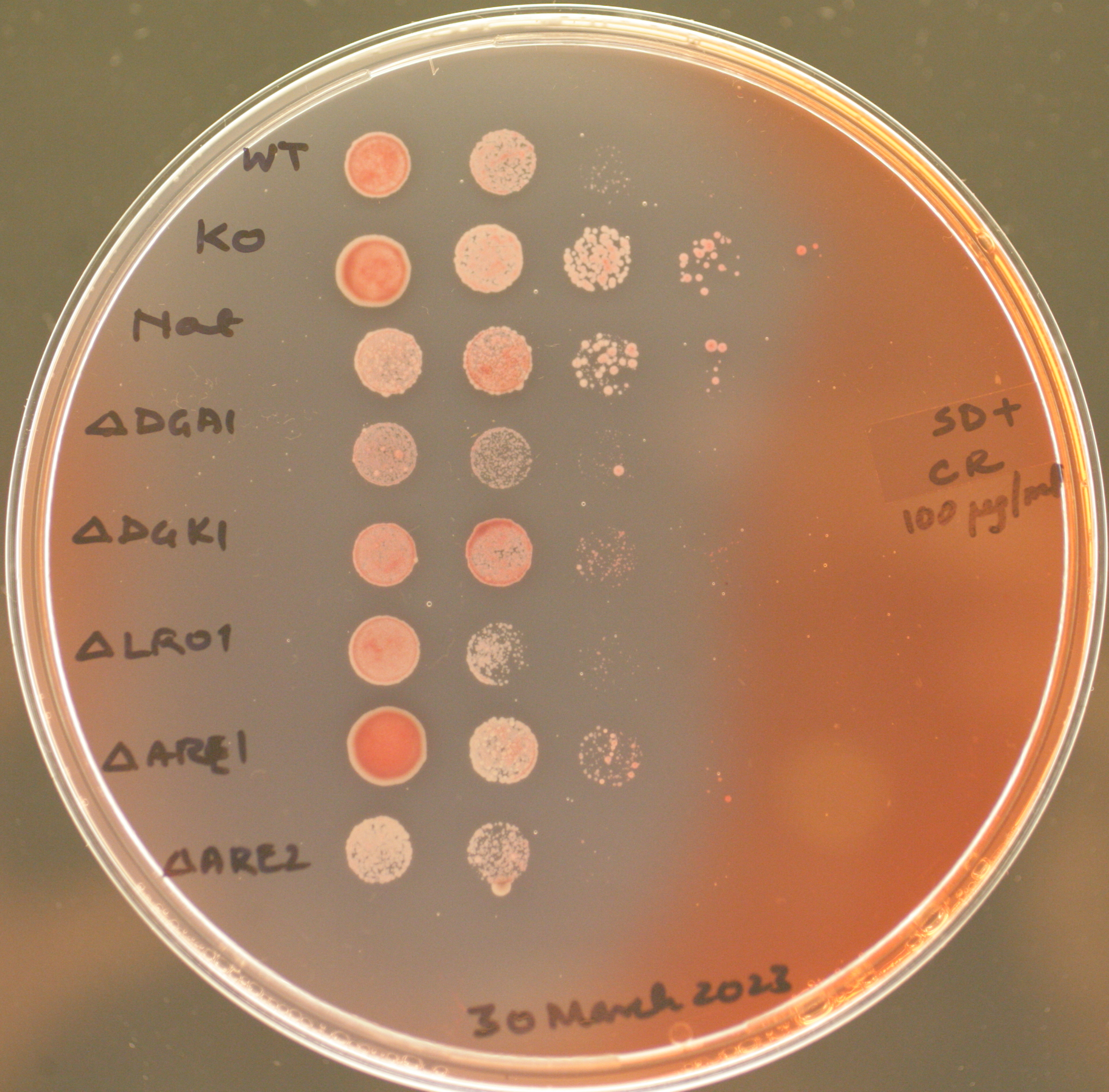

Supplement: Figure 3—figure supplement 1—source data 2. [file elife-104011-fig3-figsupp1-data2.zip › Figure 3. figure supplement 1- Source data 2/Figure 3. figure supplement 1A CR.tif]

Figure 3. figure supplement 1B

Control

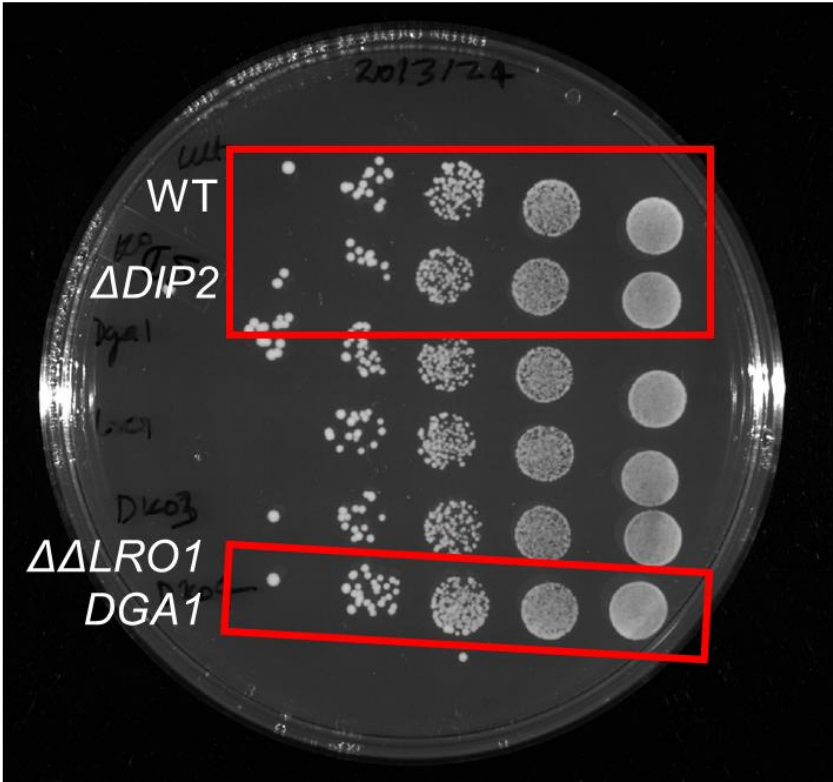

CW stress (CFW)

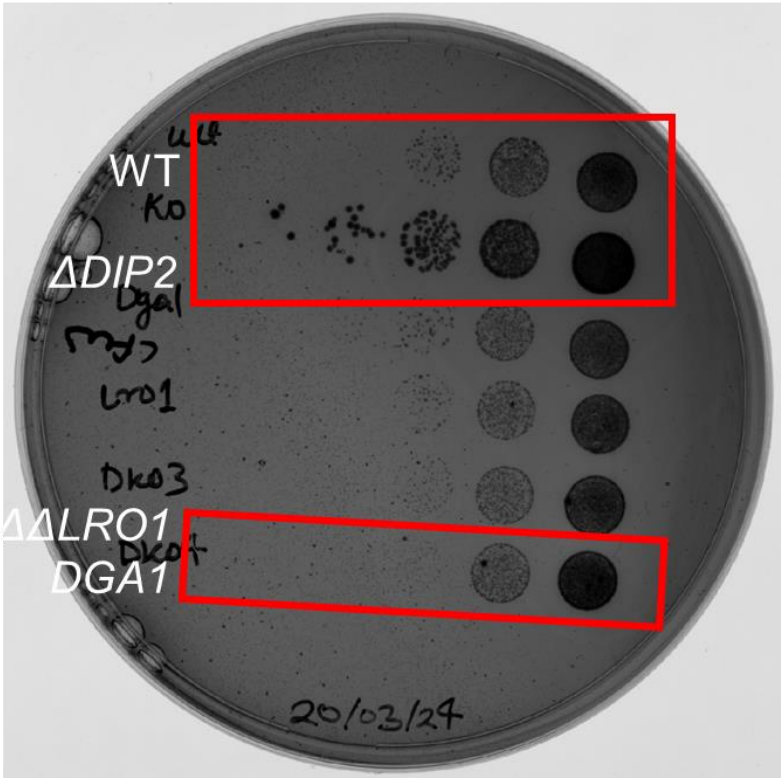

# CW stress (CR)

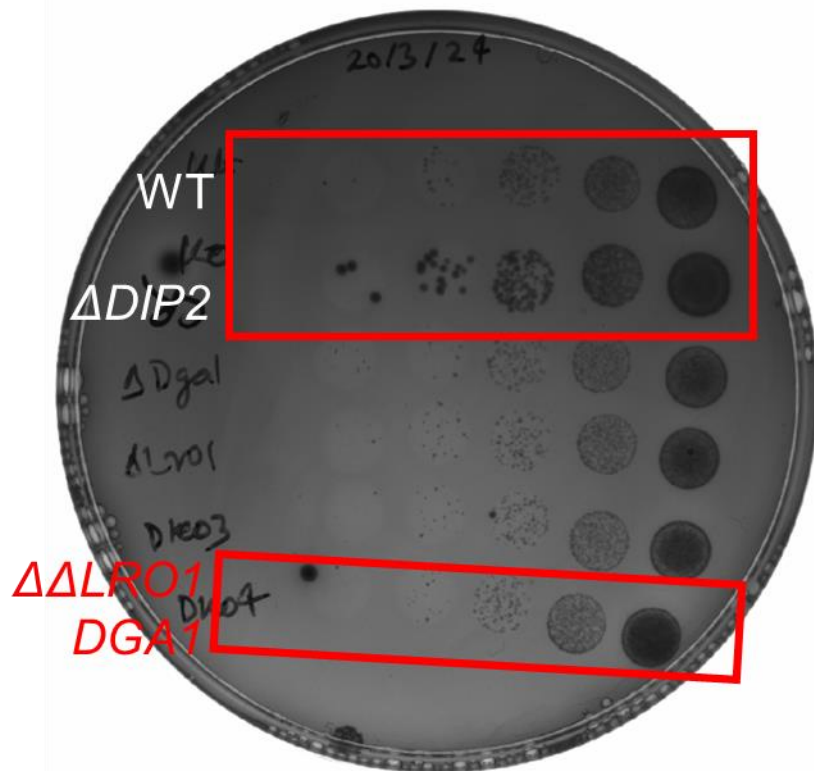

Supplement: Figure 3—figure supplement 1—source data 3. [file elife-104011-fig3-figsupp1-data3.zip › Figure 3. figure supplement 1- Source data 3/Figure 3. figure supplement 1B.pdf]

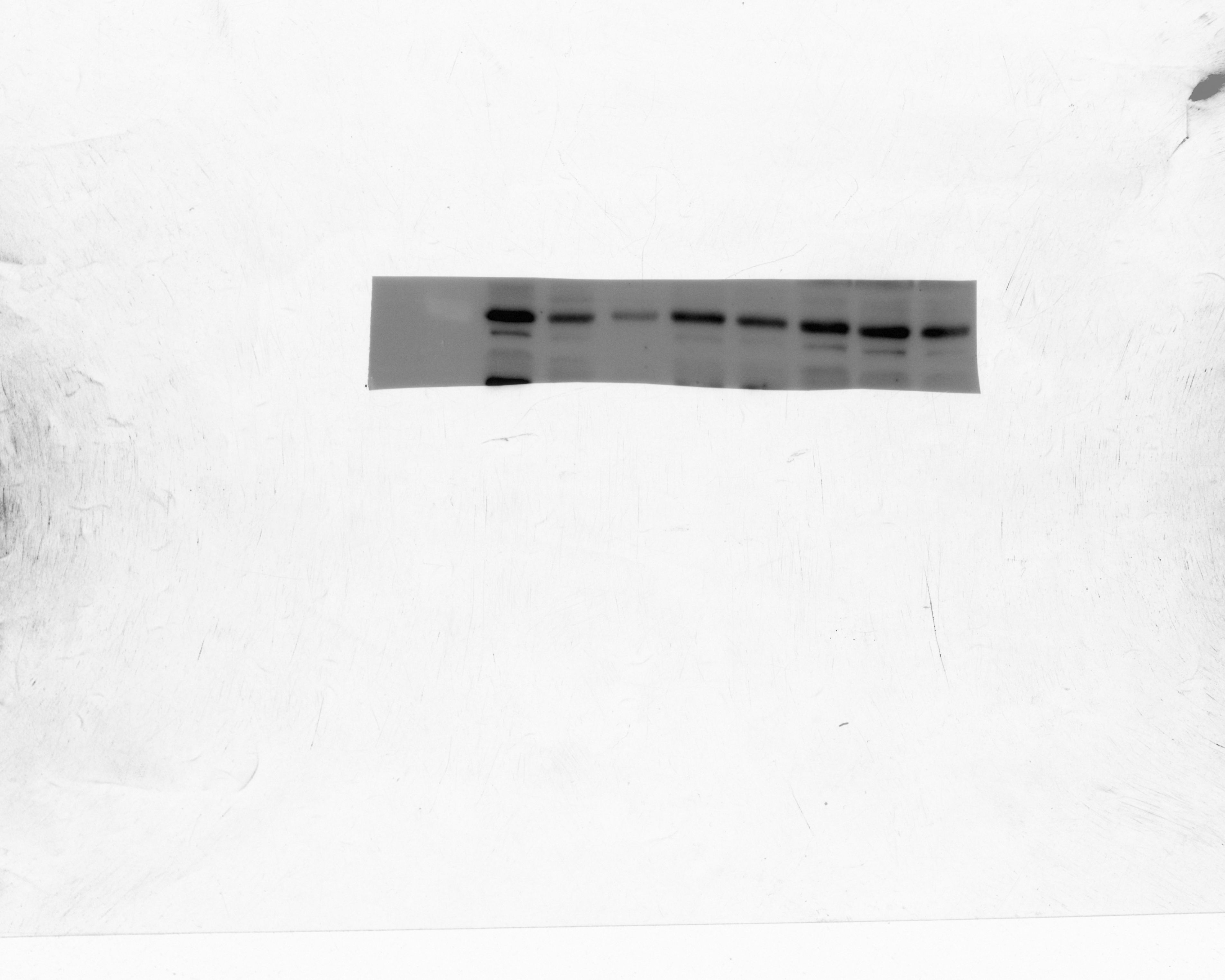

Supplement: Figure 4—source data 4. — Original files for western blot analysis displayed in Figure 4D. [file elife-104011-fig4-data4.zip › Figure 4- source data 4/Related to Fig 4D Gapdh.tif]

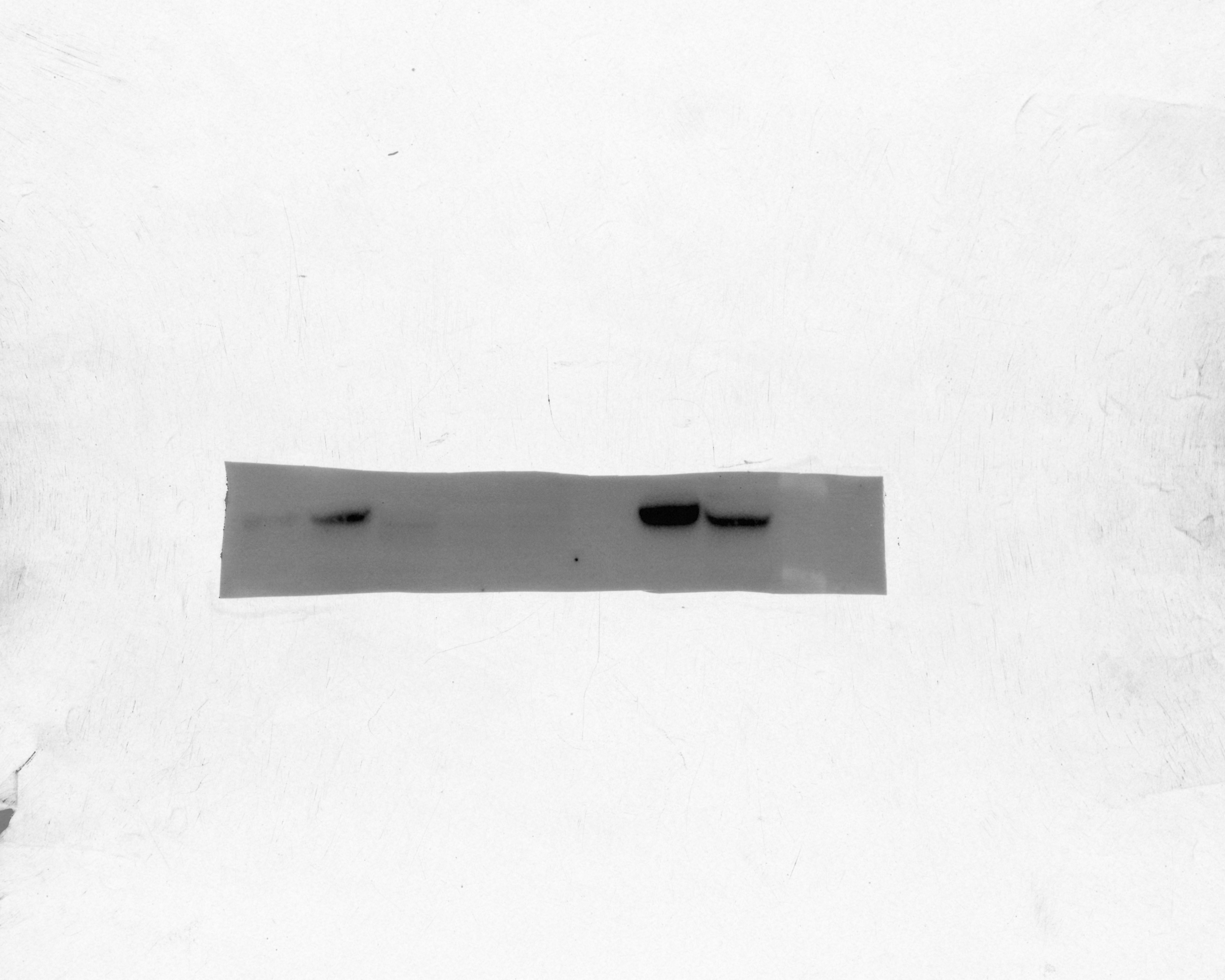

Supplement: Figure 4—source data 4. — Original files for western blot analysis displayed in Figure 4D. [file elife-104011-fig4-data4.zip › Figure 4- source data 4/Related to Fig 4D pSlt2.tif]

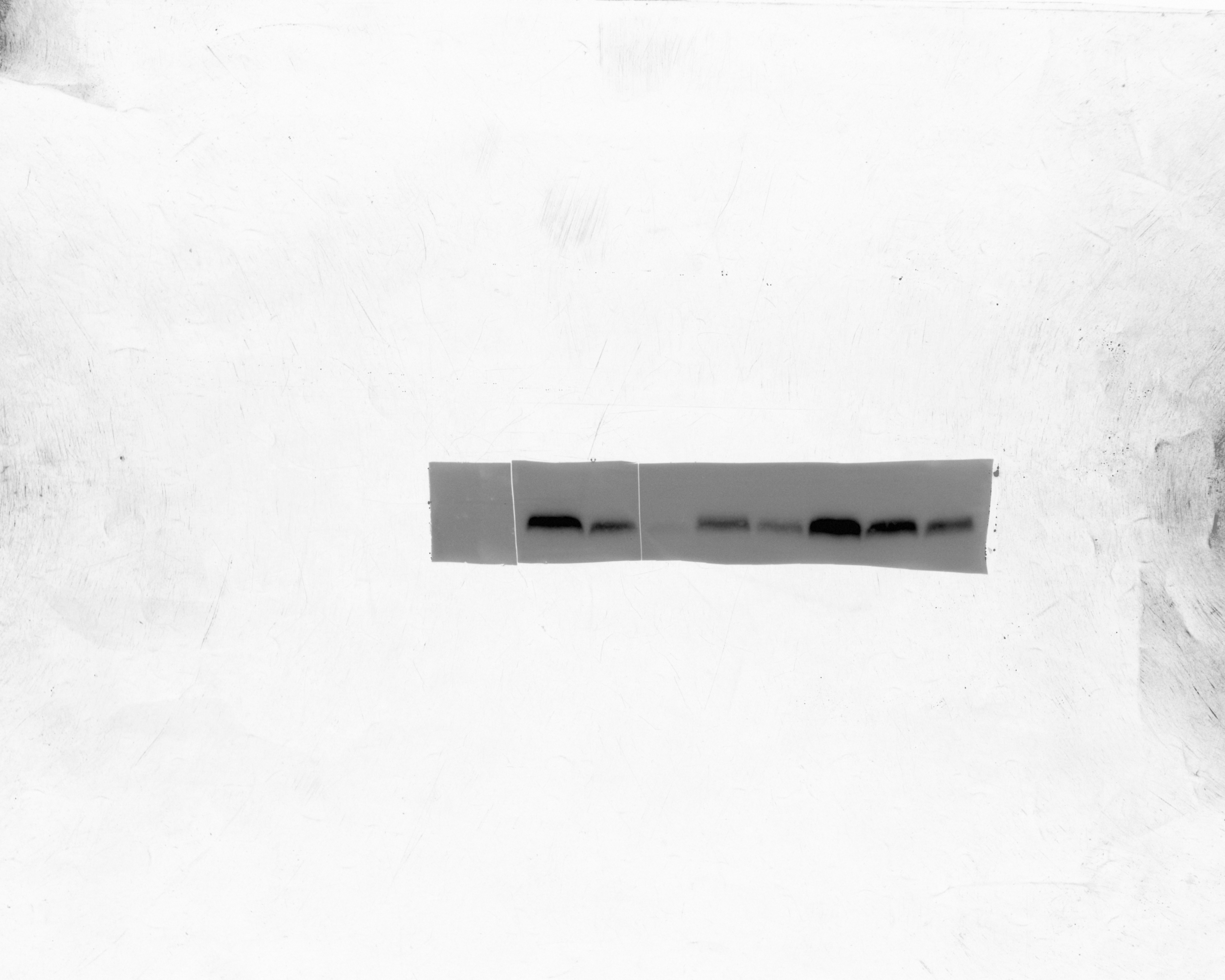

Supplement: Figure 4—source data 4. — Original files for western blot analysis displayed in Figure 4D. [file elife-104011-fig4-data4.zip › Figure 4- source data 4/Related to Fig 4D Slt2.tif]

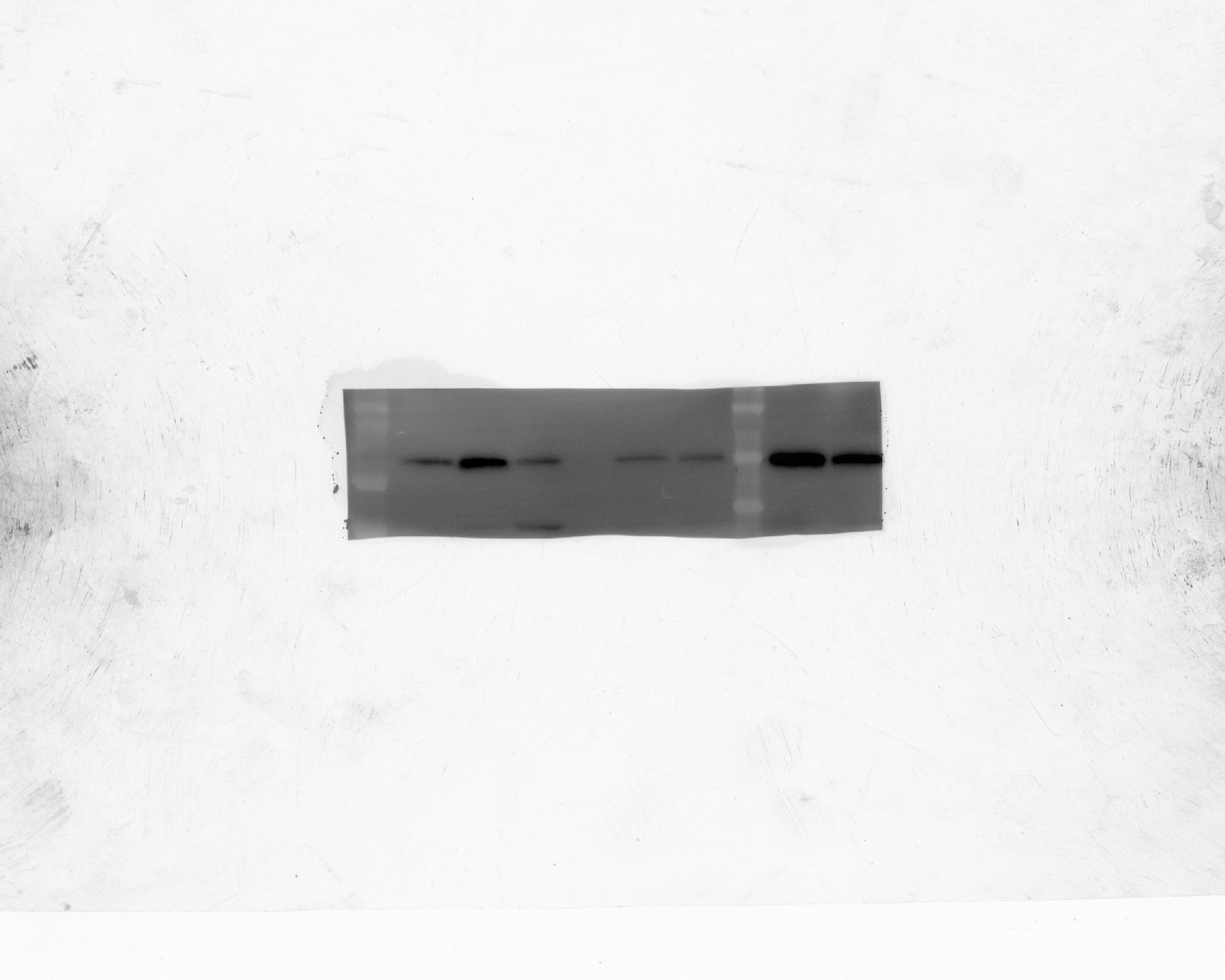

Supplement: Figure 4—source data 7. — Original files for western blot analysis displayed in Figure 4F. [file elife-104011-fig4-data7.zip › Figure 4- source data 7/Related to Fig 4F pSlt2.tif]

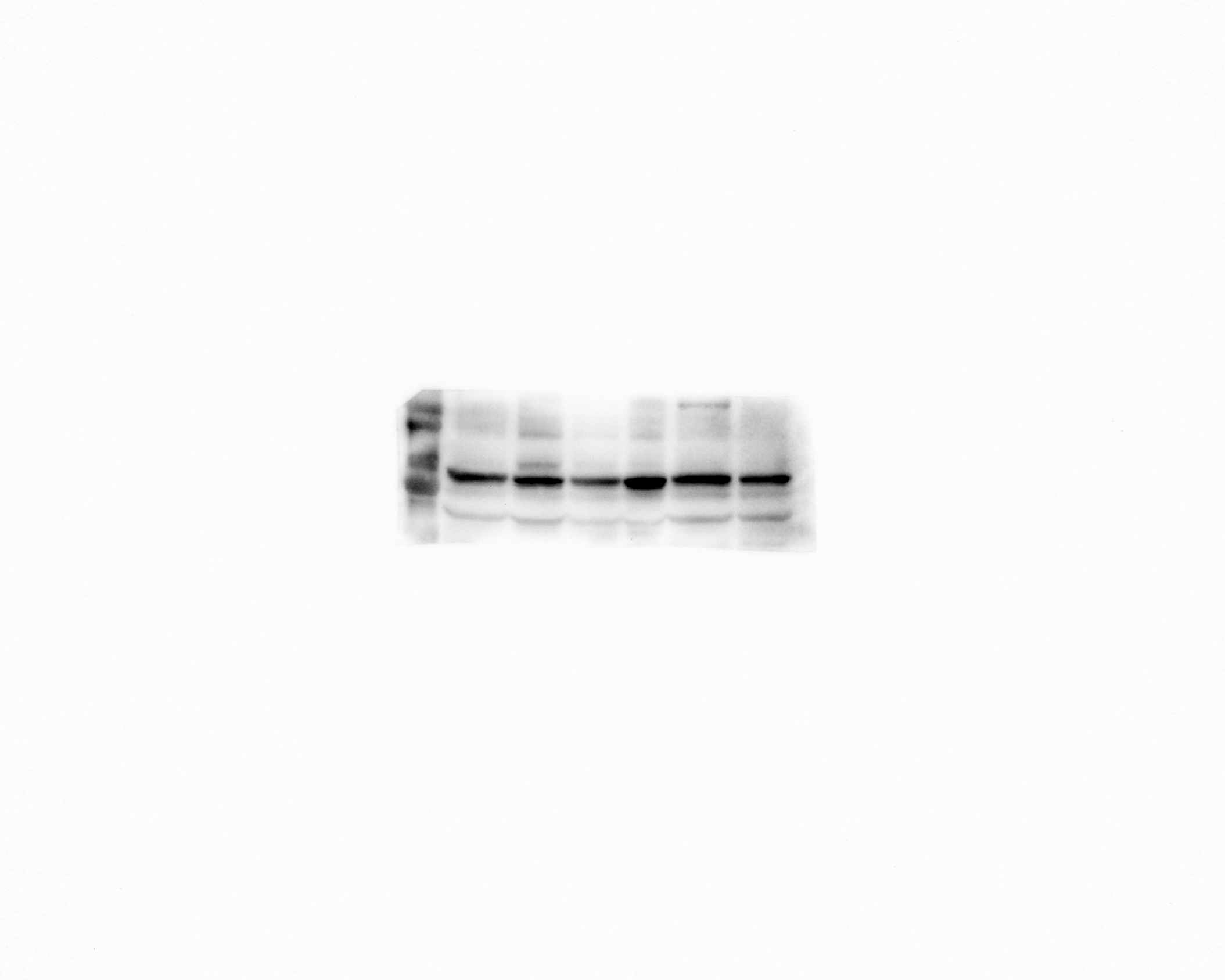

Supplement: Figure 4—source data 7. — Original files for western blot analysis displayed in Figure 4F. [file elife-104011-fig4-data7.zip › Figure 4- source data 7/Related to Fig 4F Slt2.tif]

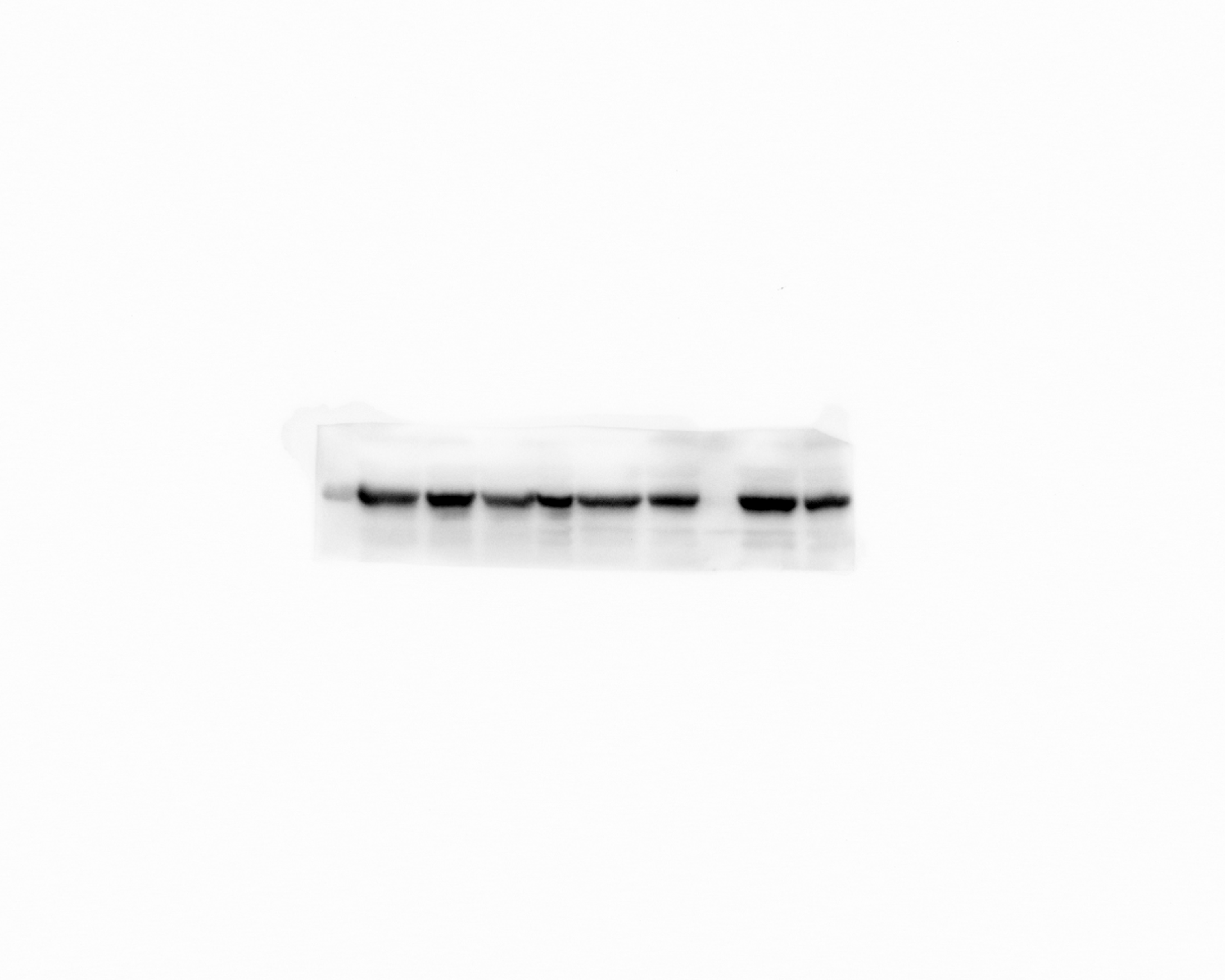

Supplement: Figure 4—source data 7. — Original files for western blot analysis displayed in Figure 4F. [file elife-104011-fig4-data7.zip › Figure 4- source data 7/Related to Fig 4F Tpi1.tif]

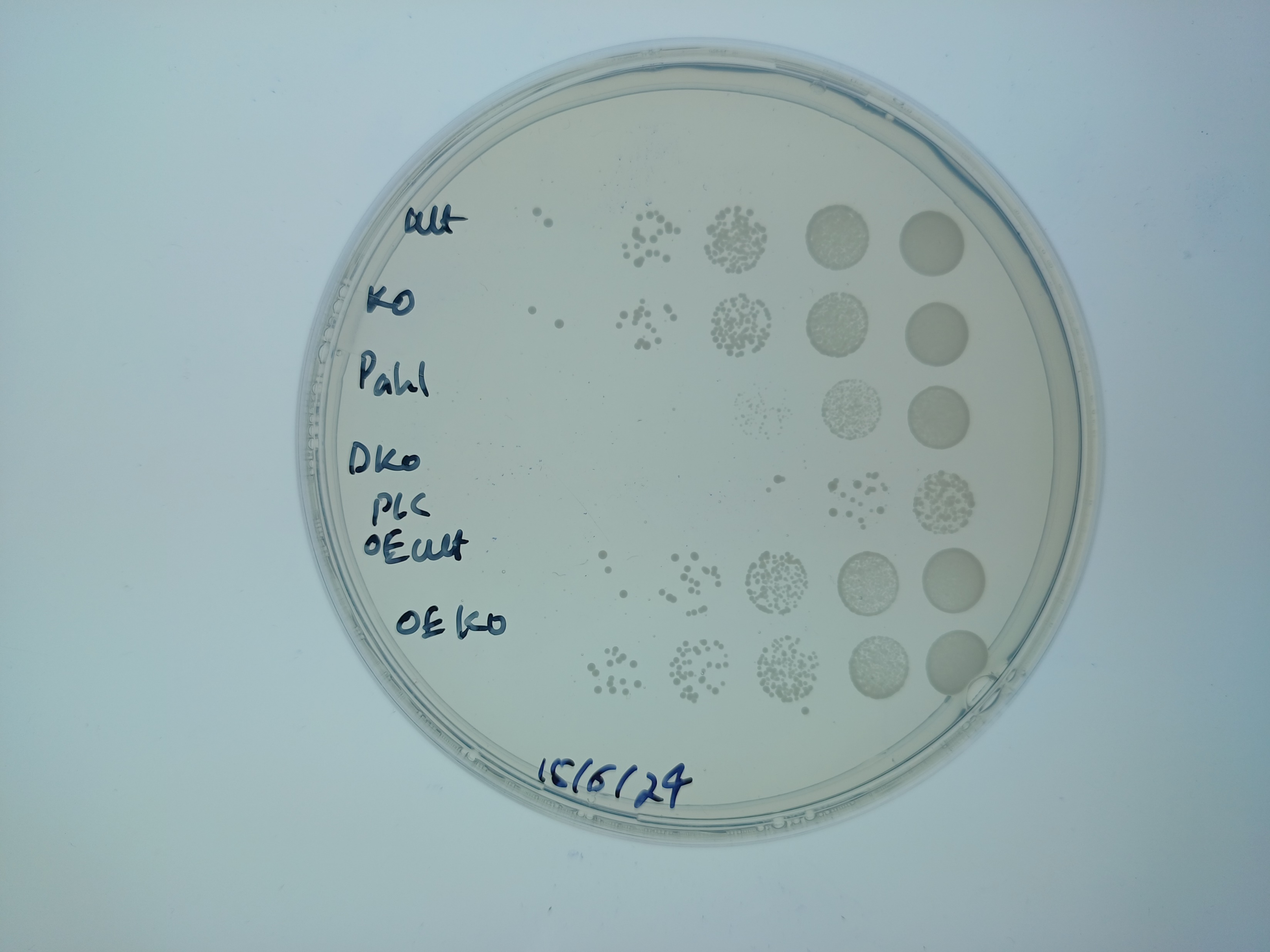

Supplement: Figure 4—figure supplement 2—source data 2. [file elife-104011-fig4-figsupp2-data2.zip › Figure 4. figure supplement 2- Source data 2/Related to Figure 4. figure supplement 2B Control.tif]

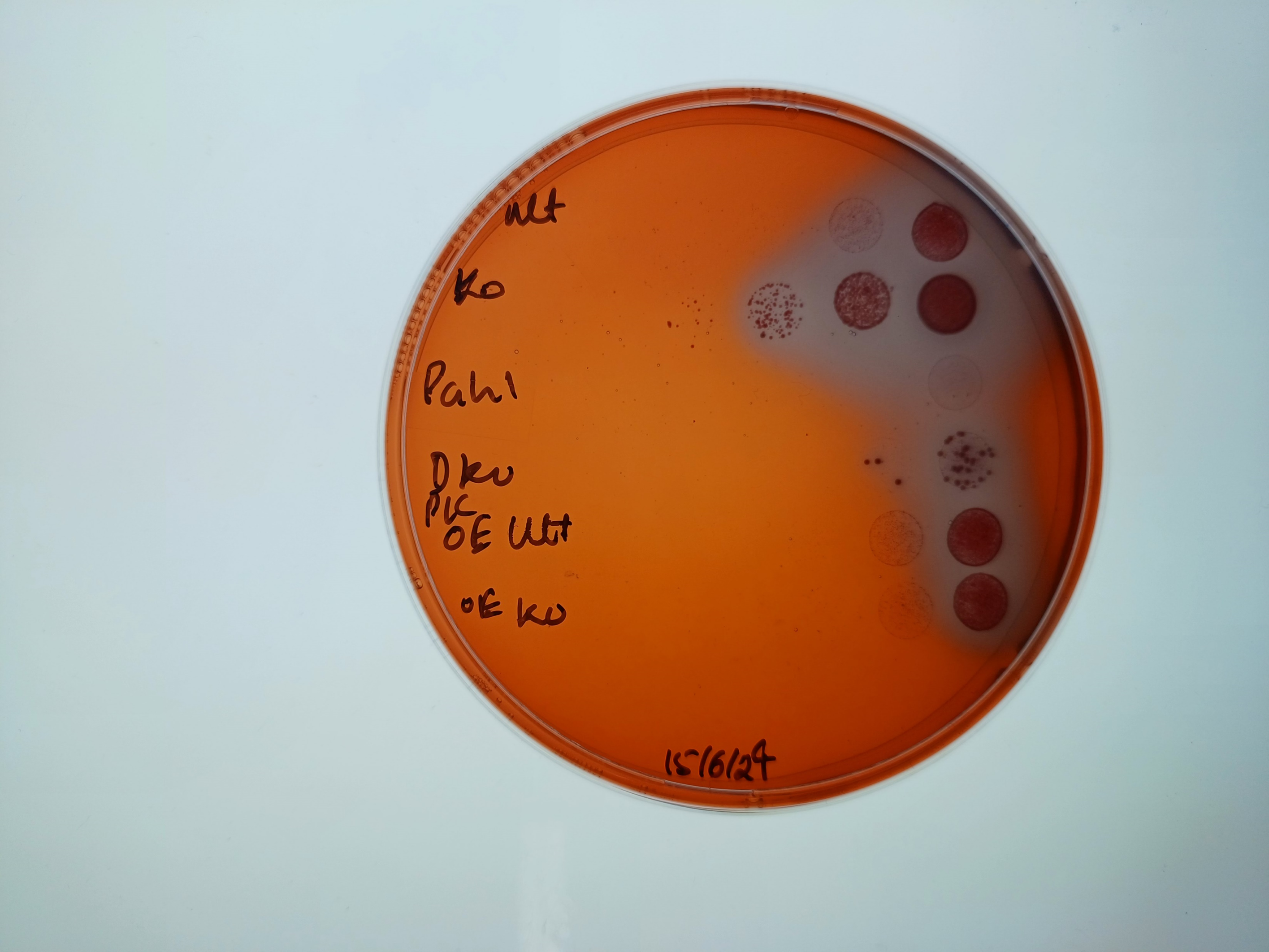

Supplement: Figure 4—figure supplement 2—source data 2. [file elife-104011-fig4-figsupp2-data2.zip › Figure 4. figure supplement 2- Source data 2/Related to Figure 4. figure supplement 2B CR.tif]

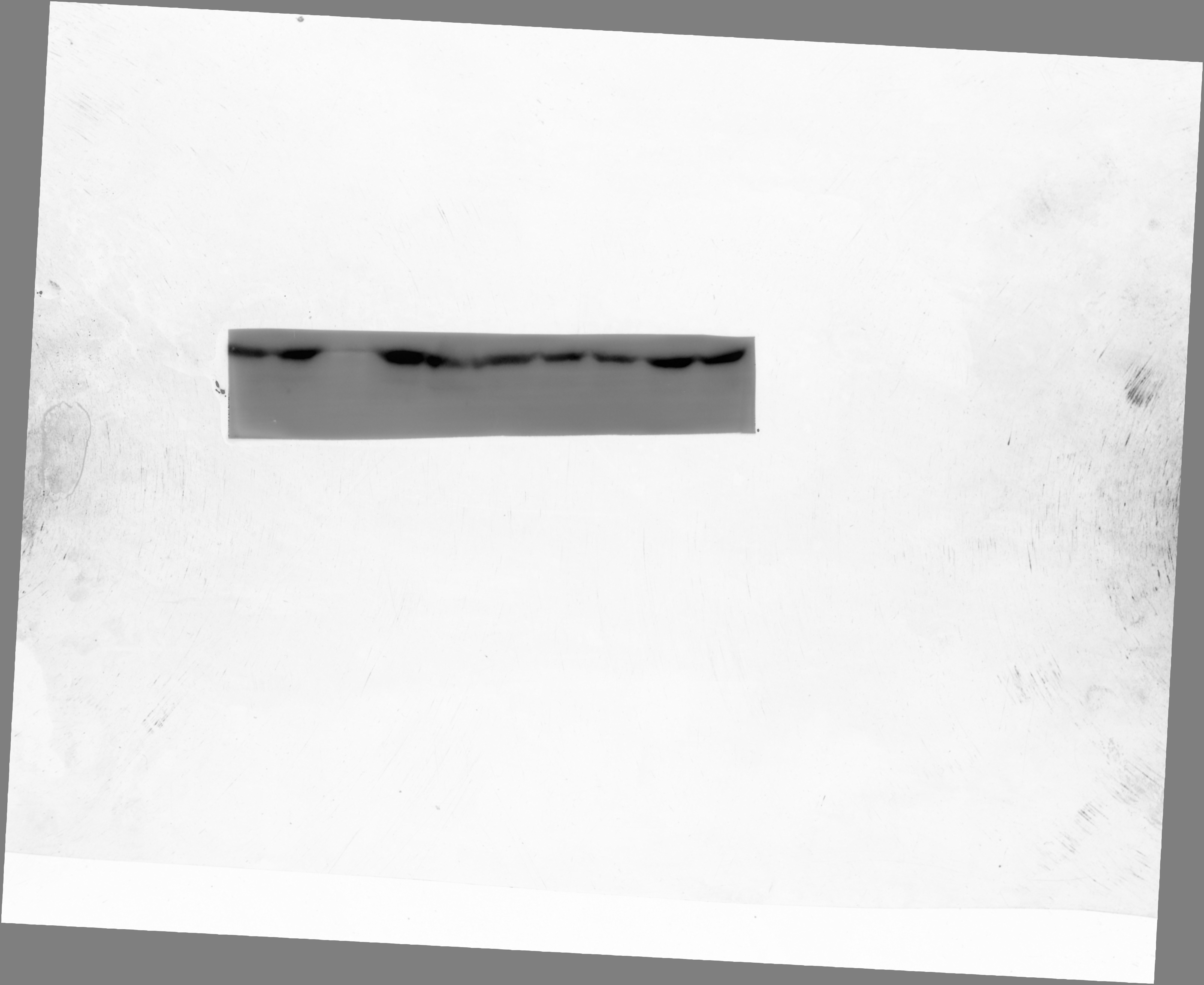

Supplement: Figure 4—figure supplement 2—source data 4. [file elife-104011-fig4-figsupp2-data4.zip › Figure 4. figure supplement 2- Source data 4/Related to Figure 4. figure supplement 2C Pgk1.tif]

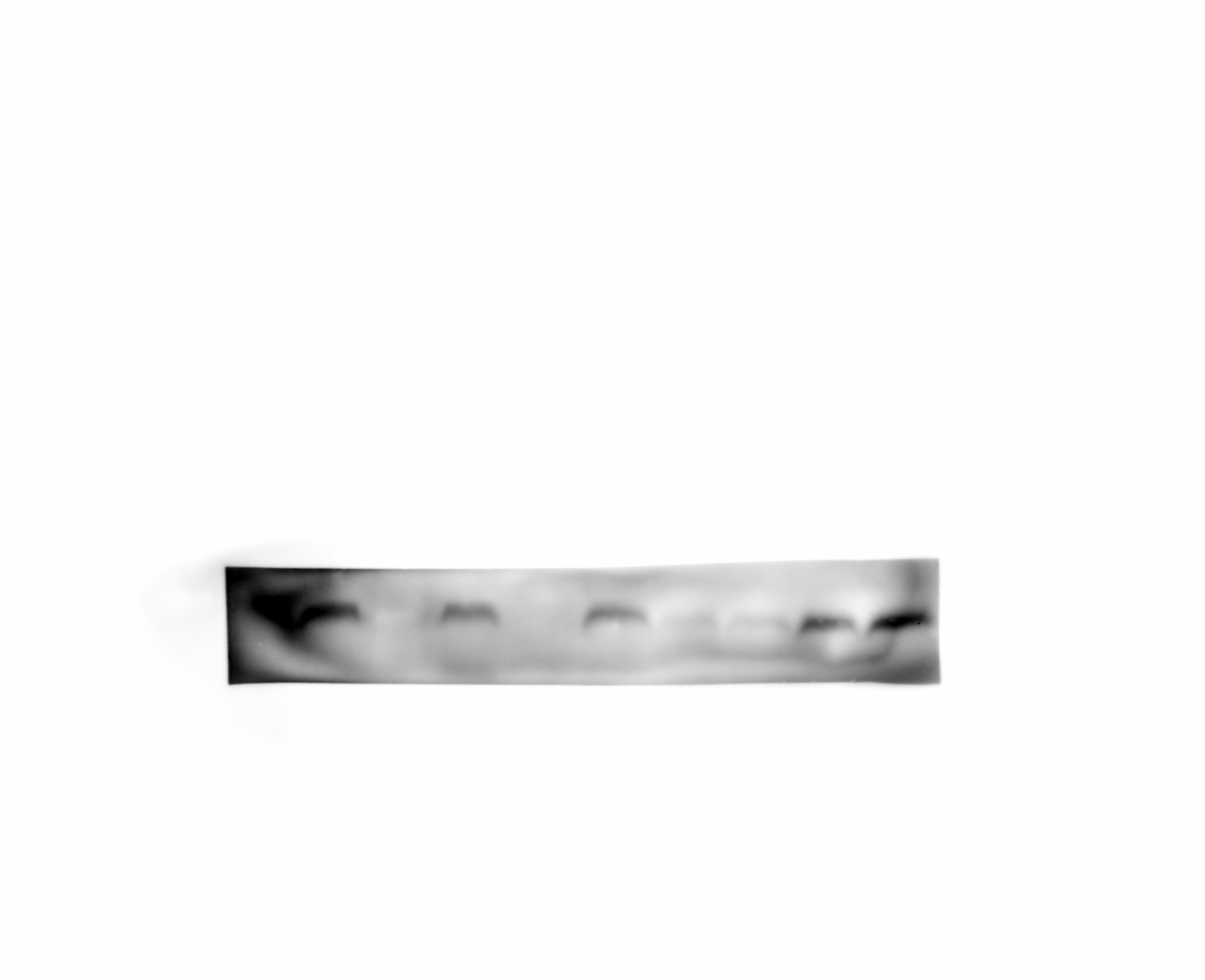

Supplement: Figure 4—figure supplement 2—source data 4. [file elife-104011-fig4-figsupp2-data4.zip › Figure 4. figure supplement 2- Source data 4/Related to Figure 4. figure supplement 2C pSlt2.tif]

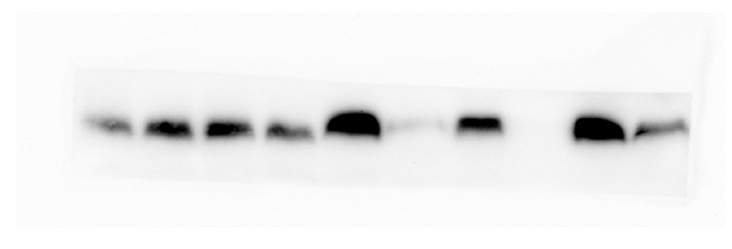

Supplement: Figure 4—figure supplement 2—source data 4. [file elife-104011-fig4-figsupp2-data4.zip › Figure 4. figure supplement 2- Source data 4/Related to Figure 4. figure supplement 2C Slt2.tif]
