# Supplementary material for: Emergence of Dip2-mediated specific DAG-based PKC signalling axis in eukaryotes
Source: eLife. 2025 May 6;14:RP104011. doi: 10.7554/eLife.104011 (PMC12055004; doi:10.7554/eLife.104011)
Supplement: Figure 3—source data 2. — PDF file containing original spot assay plate images for Figure 3D, indicating the relevant spots and treatments. [file elife-104011-fig3-data2.zip › Figure 3- source data 2/Related to 3D.pdf]

Figure 3- source data 2

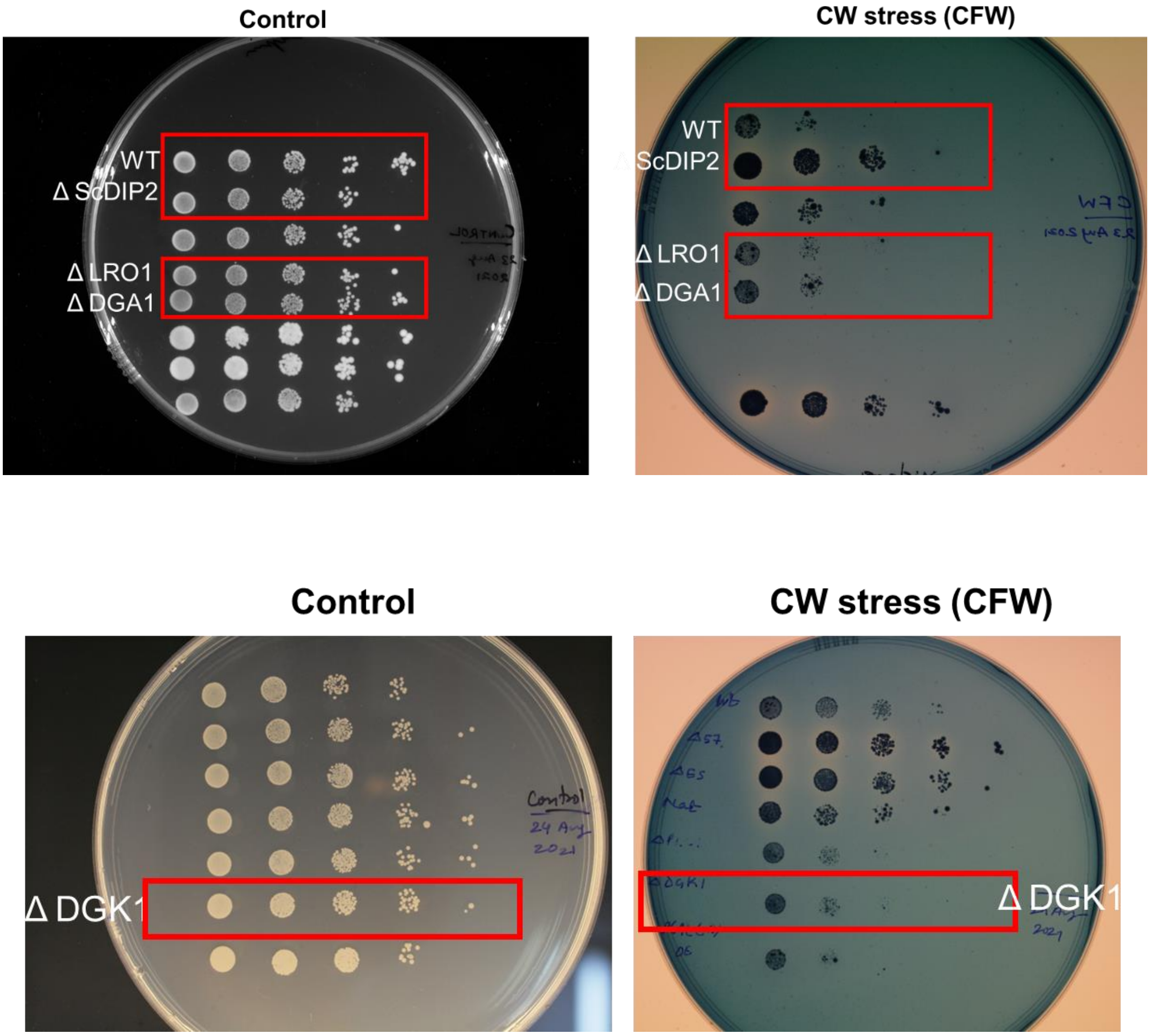

Note: ΔDGK1 control and calcofluor white image has been taken from a different plate
