# Supplementary material for: Emergence of Dip2-mediated specific DAG-based PKC signalling axis in eukaryotes
Source: eLife. 2025 May 6;14:RP104011. doi: 10.7554/eLife.104011 (PMC12055004; doi:10.7554/eLife.104011)
Supplement: Figure 3—source data 8. — PDF file containing original western blots for Figure 3H, indicating the relevant bands. [file elife-104011-fig3-data8.zip › Figure 3- source data 8/Related to Fig 3H.pdf]

Figure 3- source data 8

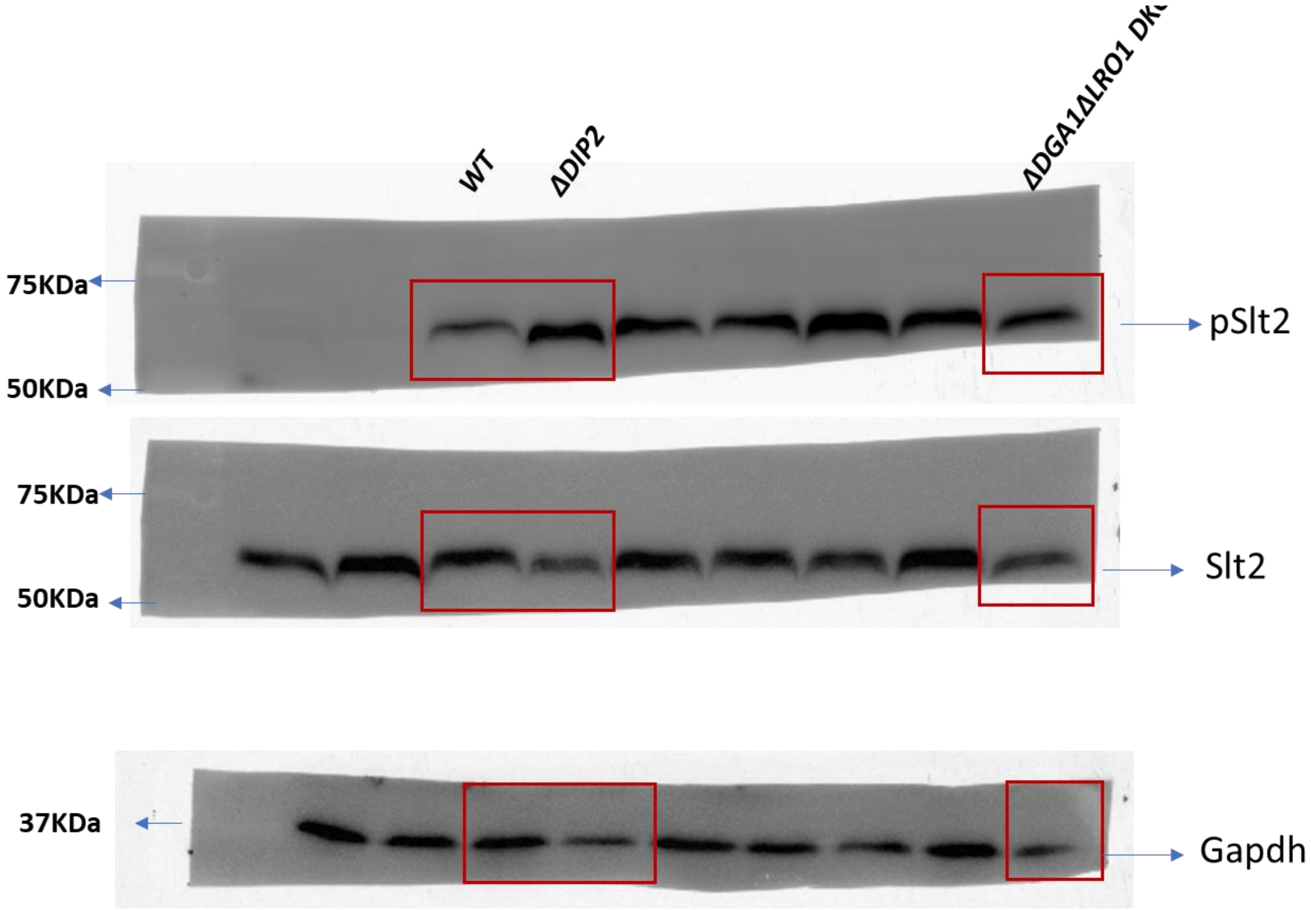

Whole blot was cut into 2 parts and probed for pSlt2 (M.W 56KDa) and Gapdh (M.W. 36 KDa).

pSlt2 blot is stripped and probed again for total Slt2 levels.
