# Supplementary material for: Emergence of Dip2-mediated specific DAG-based PKC signalling axis in eukaryotes
Source: eLife. 2025 May 6;14:RP104011. doi: 10.7554/eLife.104011 (PMC12055004; doi:10.7554/eLife.104011)
Supplement: Figure 4—source data 3. — PDF file containing original western blots for Figure 4D, indicating the relevant bands. [file elife-104011-fig4-data3.zip › Figure 4- source data 3/Figure 4- source data 3.pdf]

Figure 4- source data 3

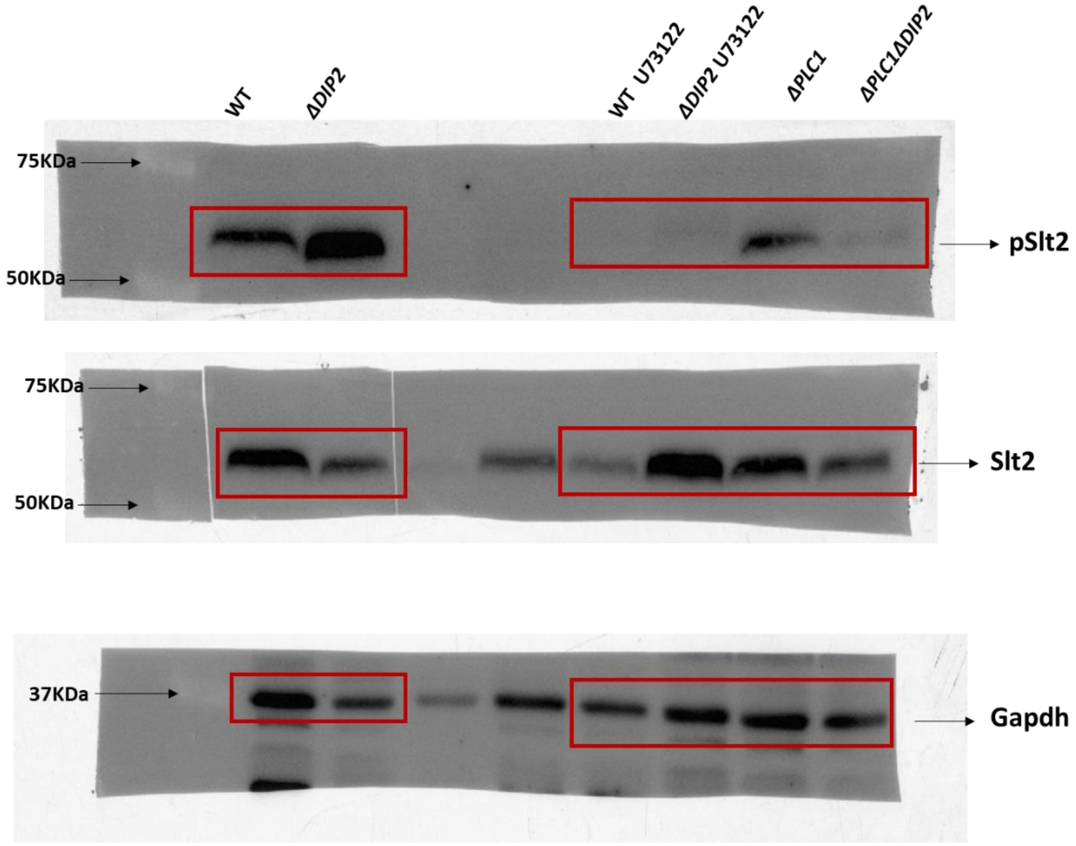

Whole blot was cut into 2 parts and probed for pSlt2 (M.W 56KDa) and Gapdh (36KDa).

The pSlt2 blot was stripped and probed again for total Slt2.

Since pSlt2 signal was missing in consecutive wells beside ΔDIP2, we decided to cut and reprobe for pSlt2 levels. Following this, we stripped and probed the cut blots together in the same box for total Slt2 levels and developed the same together.
