# Supplementary material for: Emergence of Dip2-mediated specific DAG-based PKC signalling axis in eukaryotes
Source: eLife. 2025 May 6;14:RP104011. doi: 10.7554/eLife.104011 (PMC12055004; doi:10.7554/eLife.104011)
Supplement: Figure 4—source data 6. — PDF file containing original western blots for Figure 4F, indicating the relevant bands. [file elife-104011-fig4-data6.zip › Figure 4- source data 6/Related to Fig 4F.pdf]

Figure 4- source data 6

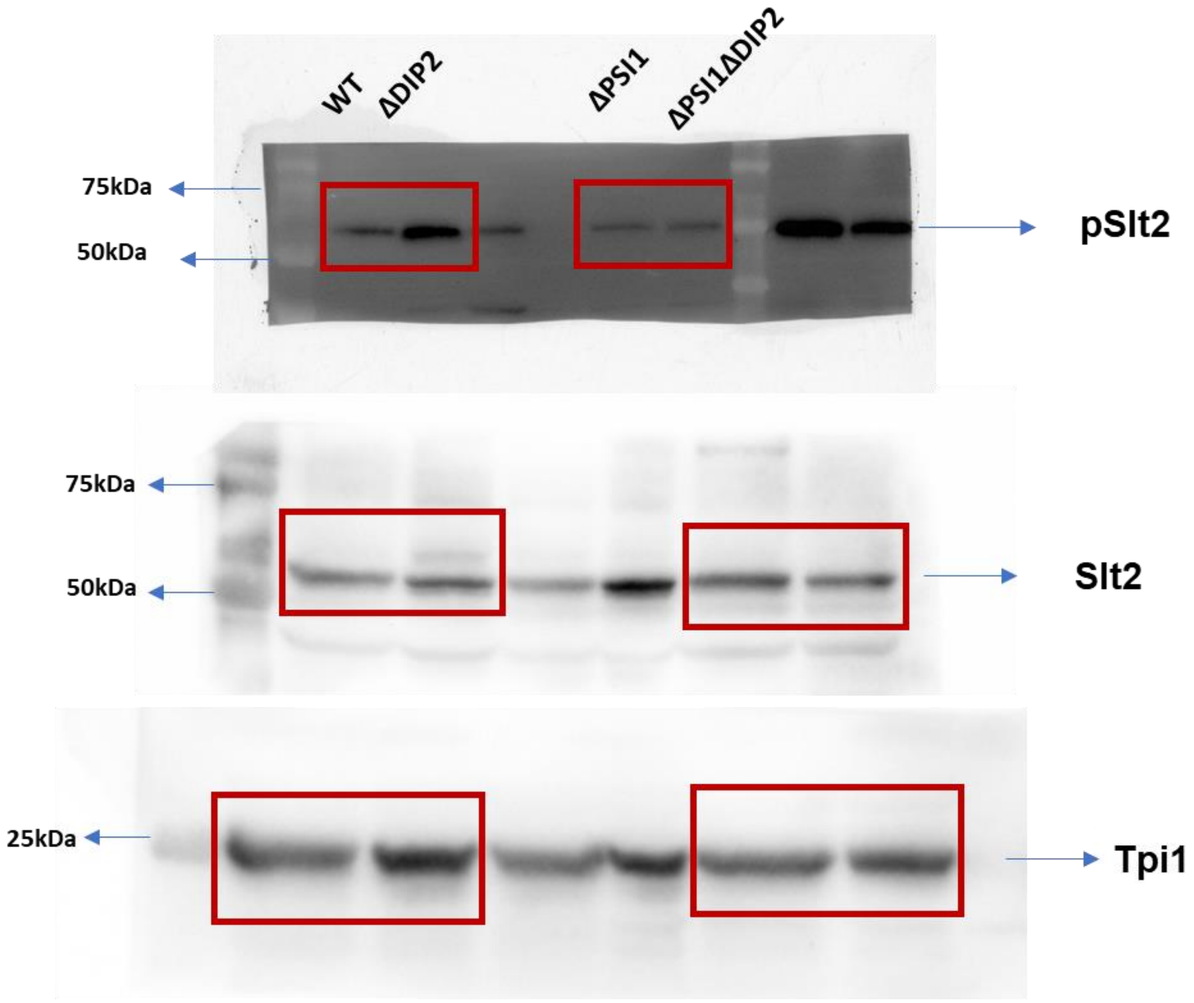

Whole blot was cut into 2 parts and probed for pSlt2 (M.W 56KDa) and Tpi1 (27KDa).

pSlt2 blot is stripped and probed again for total Slt2 levels.
