# Supplementary material for: Emergence of Dip2-mediated specific DAG-based PKC signalling axis in eukaryotes
Source: eLife. 2025 May 6;14:RP104011. doi: 10.7554/eLife.104011 (PMC12055004; doi:10.7554/eLife.104011)
Supplement: Figure 4—figure supplement 2—source data 3. [file elife-104011-fig4-figsupp2-data3.zip › Figure 4. figure supplement 2- Source data 3/Related to Figure 4. figure supplement 2C.pdf]

Figure 4. figure supplement 2

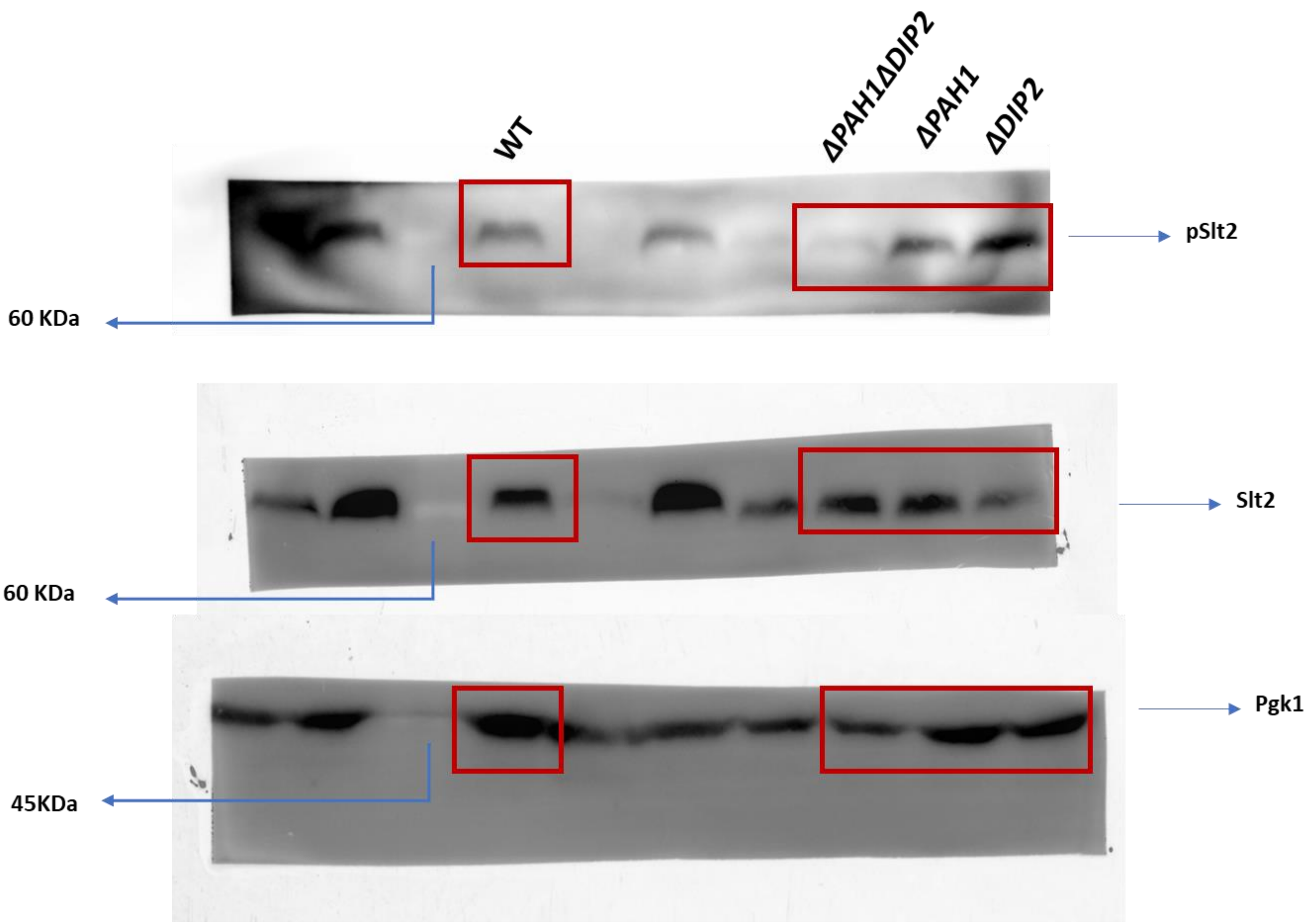

Whole blot was cut into 2 parts and probed for pSlt2 (M.W 56KDa) and Pgk1 (45 KDa).

pSlt2 blot is stripped and probed again for total Slt2 levels.
