# Supplementary material for: Emergence of Dip2-mediated specific DAG-based PKC signalling axis in eukaryotes
Source: eLife. 2025 May 6;14:RP104011. doi: 10.7554/eLife.104011 (PMC12055004; doi:10.7554/eLife.104011)
Supplement: Supplementary file 1. [file elife-104011-supp1.docx]

**Supplementary file 1: List of yeast strains**

| **Strain ID** | **Genotype** | **Source** |
| --- | --- | --- |
| *BY4741* | MATa his3∆1 leu2∆0 met15∆0 ura3∆0 | (Baker Brachmann *et al.*, 1998) |
| *YSM57 (∆dip2)* | MATa; ura3Δ0; leu2Δ0; his3Δ1; met15Δ0; YOR093c::kanMX4 | (Mondal et al., 2022) |
| *Y02501 (∆dga1)* | BY4741; MATa; ura3Δ0; leu2Δ0; his3Δ1; met15Δ0; YOR245c::kanMX4 | Euroscarf |
| *Y05383 (∆lro1)* | BY4741; MATa; ura3Δ0; leu2Δ0; his3Δ1; met15Δ0; YNR008w::kanMX4 | Euroscarf |
| *Y01608 (∆dgk1)* | BY4741; MATa; ura3Δ0; leu2Δ0; his3Δ1; met15Δ0; YOR311c::kanMX4 | Euroscarf |
| *YBR042C (∆psi1)* | BY4741; MATa; his3Δ1; leu2Δ0; met15Δ0;  ura3Δ0; YBR042c::kanMX4 | Euroscarf |
| *YPL268W (∆plc1)* | BY4741; MATa; his3Δ1; leu2Δ0; met15Δ0;  ura3Δ0; YPL268w::kanMX4 | Euroscarf |
| *(∆psi1∆dip2)* | BY4741; MATa; his3Δ1; leu2Δ0; met15Δ0;  ura3Δ0; YBR042c::kanMX4; YOR093c::hphMX6 | This study |
| *(∆plc1∆dip2)* | BY4741; MATa; his3Δ1; leu2Δ0; met15Δ0;  ura3Δ0; YPL268w::kanMX4; YOR093c::hphMX6 | This study |
| *YMR165C (∆pah1)* | BY4741; MATa; his3Δ1; leu2Δ0; met15Δ0;  ura3Δ0; YMR165c:: his3 | This study |
| *(∆pah1∆dip2)* | BY4741; MATa; his3Δ1; leu2Δ0; met15Δ0;  ura3Δ0; YMR165c:: his3  YOR093c:: kanMX4 | This study |
| *(∆dga1∆lro1)* | BY4741; MATa; his3Δ1; leu2Δ0; met15Δ0;  ura3Δ0; YMR165c:: his3  YOR093c:: kanMX4 | This study |
