# Supplementary material for: Emergence of Dip2-mediated specific DAG-based PKC signalling axis in eukaryotes
Source: eLife. 2025 May 6;14:RP104011. doi: 10.7554/eLife.104011 (PMC12055004; doi:10.7554/eLife.104011)
Supplement: Supplementary file 2. [file elife-104011-supp2.docx]

**Supplementary file 2: List of Primers**

| **Name** | **Primer sequences** |
| --- | --- |
| *DIP2* KO pFA6 FP | AGTTCTGTGTAAAAGCGTGTGGCATTGAGTTACTCCAATGCGGA TCCCCGGGTTAATTAA |
| *DIP2* KO pFA6 RP | TCCAATAGTCATGACAAATTTACTGTACTTGGATGTGTTAGAATT CGAGCTCGTTTAAAC |
| *DIP2* KO 5'UTR CHK FP | TATCTTCCCAATGTCAAAGC |
| pFA6a KO CHK RP | GAATTCGAGCTCGTTTAAAC |
| *ScDIP2-* D523A-FP | CCCATGTTAACGTTATTGGCTTTTGGTGGTATCTTTATATCTATAA GAGATCA |
| *ScDIP2*- D523A-RP | CTTATAGATATAAAGATACCACCAAAAGCCAATAACGTTAACATG GGAGAATA |
| *ScDIP2*- L687A-FP | CAAATACCTACTTTATGAGAACCAAGGCTATGGGGTTTGTTCATA ACGGAAAGAT |
| *ScDIP2*- L687A-RP | CCGTTATGAACAAACCCCATAGCCTTGGTTCTCATAAAGTAGGTA TTTGCAGGAC |
| *ScPKC1* C1a-C1b FP | ATGCTTAATTAAATGCATGGCCACCACTTTGTAC |
| *ScPKC1* C1a-C1b RP | AGTCCTCGAGCGACATGCCACAGAAATCGG |

| *PAH1* 5'UTR CHK | FCCTAACACTGAGCGTTCTTG |
| --- | --- |
| *PAH1* KO pFA6 FP | FGGGAAGAAATTACTGAAGATAGACACATCGGTCGATTATGCGG ATCCCCGGGTTAATTAA |
| *PAH1* KO pFA6 RP | ATGGATCGTTATAAATAATATTCGGCTACAAGAATCTTTAGAATTC GAGCTCGTTTAAAC |
| *LRO1* 5'UTR CHK | CACCTGCTTGTACAAACTCC |
| *LRO1* KO pFA6 FP | TTCTCTACCAACGAATTCGGCGACAATCGAGTAAAAAATGCGGATCCCCG GGTTAATTAA |
| *LRO1* KO pFA6 RP | ATAATACACGGATGGATAGTGAGTCAATGTCGGTCATTTAGAATTCGAGCT CGTTTAAAC |
| pETite C1 98E  GB nFP | TTTAACTATAAGAAGGAGATATACATATGGTCCATCAGGTGAATG GG |
| pETite C1 98E  GB nRP | TTGGAAGTACAGGTTTTCGCTAGCGTTGATGCCGCACGTG |
| pETite C1 delta  GB nFP | TTTAACTATAAGAAGGAGATATACATATGACCATGGGGGCCCAC |
| pETite C1 delta  GB nRP | TTGGAAGTACAGGTTTTCGCTAGCCCTTGCGCCGGCAC |
