# Supplementary material for: Emergence of Dip2-mediated specific DAG-based PKC signalling axis in eukaryotes
Source: eLife. 2025 May 6;14:RP104011. doi: 10.7554/eLife.104011 (PMC12055004; doi:10.7554/eLife.104011)
Supplement: Supplementary file 3. [file elife-104011-supp3.docx]

**Supplementary file 3: List of plasmids.**

| **Plasmid ID** | **Description** | **Reference** |
| --- | --- | --- |
| pYSM5 | *pGAL1-MCS-TEV-GFP-8XHis-2XHA* | Mondal et al, 2022 |
| pYSM7 | *pPPM90-PROMOTER-ScDIP2- TERMINATOR* | Mondal et al, 2022 |
| pYSM10 | *pGAL1-ScDIP2-TEV-GFP-8XHis-2XHA* | Mondal et al, 2022 |
| pYSS01 | *pDIP2-ScDIP2-TEV-GFP-8XHis-2XHA* | This study |
| pYSS02 | *pDIP2-ScDIP2 D523A-TEV-GFP-8XHis-2XHA* | This study |
| pYSS03 | *pDIP2-ScDIP2 L687A-TEV-GFP-8XHis-2XHA* | This study |
|  | *pFA6A-hphMX6* | Gift from Dr. Palani Murgan, CSIR-CCMB |
| (Addgene plasmid #41596) | *pFA6a-His3MX6* | Addgene |
|  | *Petite_THisHA* |  |
