# Supplementary material for: Emergence of Dip2-mediated specific DAG-based PKC signalling axis in eukaryotes
Source: eLife. 2025 May 6;14:RP104011. doi: 10.7554/eLife.104011 (PMC12055004; doi:10.7554/eLife.104011)
Supplement: Supplementary file 4. [file elife-104011-supp4.docx]

**Supplementary file 4: Software and algorithms**

| **Softwares** | **Source** | **Identifier** |
| --- | --- | --- |
| SnapGene | SnapGene | [www.snapgene.com](http://www.snapgene.com/) |
| Adobe Photoshop | Adobe Systems Incorporated | [www.adobe.com/de/Photoshop](http://www.adobe.com/de/Photoshop) |
| Adobe Illustrator | Adobe Systems Incorporated | [www.adobe.com/de/Illustrator](http://www.adobe.com/de/Illustrator) |
| GraphPad Prism |  | https://[www.graphpad.com/](http://www.graphpad.com/)  scientific-software/prism/ |
| Image Lab | BioRad | https://[www.bio-rad.com/en-us/product/](http://www.bio-rad.com/en-us/product/)  image-lab-software?ID=KRE6P5E8Z |
| Saccharomyces Genome Database (SGD) |  | https://[www.yeastgenome.org/](http://www.yeastgenome.org/) |
| Primers-4- Yeast |  | https://[www.weizmann.ac.il/Primers-4-Yeast/](http://www.weizmann.ac.il/Primers-4-Yeast/) |
| BlastP |  | https://blast.ncbi.nlm.nih.gov/Blast.cgi?PAGE=Proteins |
| MAFFT |  | https://[www.ebi.ac.uk/Tools/msa/mafft/](http://www.ebi.ac.uk/Tools/msa/mafft/) |
| MEGA |  | https://[www.megasoftware.net/](http://www.megasoftware.net/) |
| IQ-TREE |  | <http://www.iqtree.org/> |
| iTOL |  | https://itol.embl.de/itol.cgi |
| Conserved Domains Database |  | https://[www.ncbi.nlm.nih.gov/Structure/cdd/wrpsb.cgi](http://www.ncbi.nlm.nih.gov/Structure/cdd/wrpsb.cgi) |
| BioRender |  | https://[www.biorender.com/](http://www.biorender.com/) |
