# Supplementary material for: Emergence of Dip2-mediated specific DAG-based PKC signalling axis in eukaryotes
Source: eLife. 2025 May 6;14:RP104011. doi: 10.7554/eLife.104011 (PMC12055004; doi:10.7554/eLife.104011)
Supplement: Supplementary file 5. [file elife-104011-supp5.docx]

**Supplementary file 5: Reagents or Resources**

| **Reagent or Resources** | **Source** | **Identifier** |
| --- | --- | --- |
| G418 | HIMEDIA | Cat#TC05 |
| Hygromycin B | Sigma | Cat#10843555001 |
| Congo Red | Sigma | Cat#C6767 |
| Calcofluor White | Sigma | Cat#18909 |
| D-(+)-Glucose | Sigma | Cat#G5767 |
| Peptone | HIMEDIA | Cat#RM001 |
| Yeast extract | HIMEDIA | Cat#RM027 |
| Bacto agar | BD | Cat#1265201 |
| YNB w/o amino acids | BD Difco | Cat#291940 |
| Adenine | Sigma | Cat#A5665 |
| Uracil | Sigma | Cat#U0750 |
| U73122 | Sigma | Cat#662035 |
| Aureobasidin A | Takara | Cat#630499 |
| Propranolol | Sigma | Cat#318989 |
| NaCl | Sigma | Cat#S1679 |
| PMSF | Sigma | Cat#P7626 |
| Protease Inhibitor Cocktail | Sigma | Cat# P2714 |
| Sodium Orthovanadate | Sigma | Cat#S6508 |
| Sodium Pyrophosphate | Sigma | Cat#P8010 |
| Sodium Fluoride | Sigma | Cat#71519 |
| Chloroform | Spectrochem | Cat#050304 |
| Methanol | Biosolve | Cat#136841G5 |
| Formic acid | Sigma | Cat#5438040100 |

| Glass beads (acid washed) | Sigma | Cat#G8772 |
| --- | --- | --- |
| Bradford reagent | Sigma | Cat#B6916 |
| Anti-Phospho-p44/42 MAPK (Erk1/2) (Thr202/Tyr204) | Cell Signaling Technologies | Cat#9101 |
| Anti-p44/42 MAPK (Erk1/2) Antibody | Cell Signaling Technologies | Cat#9102 |
| Mpk1 Antibody (D-1) | Santa cruz | sc-374434 |
| Anti-rabbit IgG, HRP-linked Antibody (1:10000) | Cell Signaling Technologies | Cat# 7074 |
| Anti-TPI1 Antibody |  | Gift from Dr. Palani Murugan Rangasamy |
| SuperSignal™ West Pico PLUS Chemiluminescent Substrate | Thermo Scientific | Cat#34580 |
| 1-palmitoyl-2-oleoyl-glycero-3- phosphocholine | Avanti Polar lipids | 850457P-200MG |
| 1,2-dipalmitoyl-sn- glycerol | Sigma | 800816P-25MG |
| 1-palmitoyl-2-oleoyl-sn-glycerol | Sigma | 800815O-10MG |
| 1-octadecanoyl-2-hexadecanoyl-sn- glycerol | Sigma | 800821C-5MG |
| rac1,2 distearoyl glycerol | Sigma | 43697-250MG |
